# Cyclopropenes for the Stepwise Synthesis of 1,2,4,5-Tetraarylbenzenes via 1,4-Cyclohexadienes

**DOI:** 10.1021/acs.joc.2c01261

**Published:** 2022-10-06

**Authors:** Satoshi Kishida, Misaki Takano, Takuya Sekiya, Yutaka Ukaji, Kohei Endo

**Affiliations:** † Department of Chemistry, Faculty of Science, Tokyo University of Science, Tokyo 162-8601, Japan; ‡ Division of Material Chemistry, Graduate School of Natural Science and Technology, Kanazawa University, Kakuma, Kanazawa, Ishikawa 920-1192, Japan

## Abstract

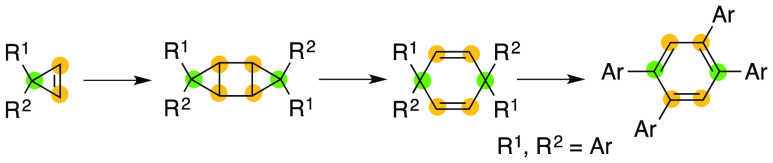

This paper describes a synthetic approach to the synthesis of 1,2,4,5-tetraarylbenzene derivatives from cyclopropenes. The Lewis acid-mediated dimerization of cyclopropenes gives tricyclo[3.1.0.0^2,4^]hexane derivatives. The subsequent thermal ring-opening reaction under solvent-free conditions gives 1,4-cyclohexadienes bearing quaternary carbons. The novel Br_2_-mediated oxidative rearrangement of 1,4-cyclohexadienes takes place to give 1,2,4,5-tetraarylbenzene derivatives in high to excellent yields.

Cyclohexadienes are promising precursors for transition-metal complexes as well as for the synthesis of a benzene core via aromatization.^1,2^ We attempted to synthesize 1,4-cyclohexadienes bearing quaternary carbons for use in the synthesis of ligands, 1,2,4,5-tetraarylbenzene derivatives **4**, and the corresponding π-extended molecules via the oxidative rearrangement reaction of 1,4-cyclohexadienes **3**, as described in Figure 1.^2^ A few effective synthetic methods are available for the preparation of 1,4-cyclohexadienes bearing quaternary carbons. The Birch reduction of benzene derivatives or nucleophilic addition to quinone derivatives are typical approaches to the synthesis of 1,4-cyclohexadienes bearing quaternary carbons.^3^ In this context, we wondered if 1,4-cyclohexadienes **3** would be obtained via the ring-opening reaction of tricyclo[3.1.0.0^2,4^]hexane derivatives **2**, which could be obtained as side products in a several reactions performed with cyclopropenes.^4,5^ In addition, the photodimerization of cyclopropenes gives tricyclo[3.1.0.0^2,4^]hexane derivatives in low to moderate yields and selectivities, respectively.^6^ The present paper describes easy access to 1,2,4,5-tetraarylbenzenes **4** via the Lewis acid-mediated dimerization of cyclopropenes **1**, the thermal ring-opening of tricyclo[3.1.0.0^2,4^]hexane derivatives **2**, and the oxidative rearrangement of 1,4-cyclohexadienes **3**.

**Figure 1:**
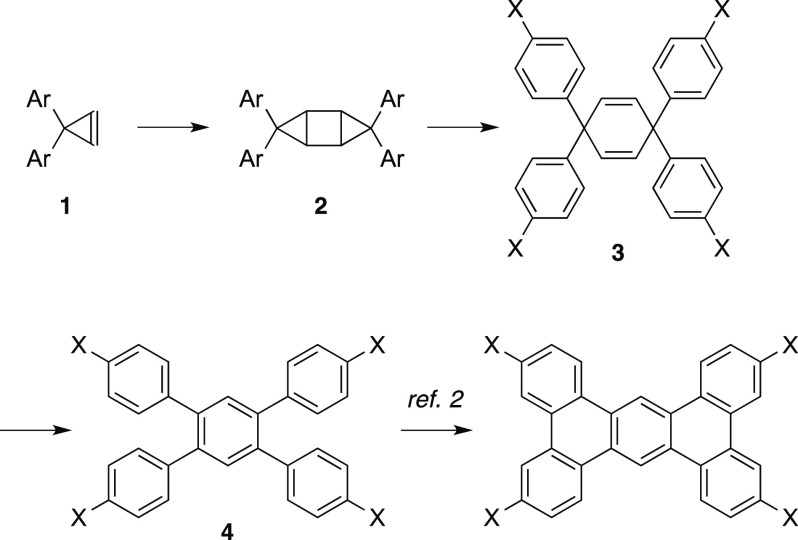
Synthetic approach to 1,2,4,5-tetraarylbenzenes **4**.

The screening of Lewis acids for the [2 + 2]-type dimerization of cyclopropenes is shown in Table 1. The reaction of cyclopropene **1a** (0.2 mmol) in THF proceeded at room temperature and under reflux conditions in the presence of Me_3_Al (1 equiv) (entries 1 and 2, respectively). A catalytic amount of Me_3_Al gave low yields, even with a long reaction time (entries 3 and 4). The decomposition of Me_3_Al in THF at reflux conditions seems to be inevitable; thus, the present reaction must finish in the shortest time possible. Other Lewis acids or catalysts did not give improved yields (entries 5–16). Therefore, the use of Me_3_Al (1 equiv) in THF under reflux conditions is suitable for the present [2 + 2]-type dimerization reaction of cyclopropenes. The present reaction might proceed via the activation of a cyclopropene moiety with Me_3_Al, which would generate a cyclopropylaluminum intermediate (Figure 2).^7^ Although there is no clear evidence for the mechanism, further reaction with another cyclopropene molecule and the subsequent elimination of Me_3_Al would generate the desired product. The speculated mechanistic rationale suggests that a catalytic amount of Lewis acid can promote the present reaction, but the experimental results showed that a catalytic amount of Lewis acid was not effective due to the loss of activity during the long reaction time. The dimerization reaction of other alkenes with Me_3_Al, such as stilbene or acenaphthylene, did not proceed.

**Table 1:**
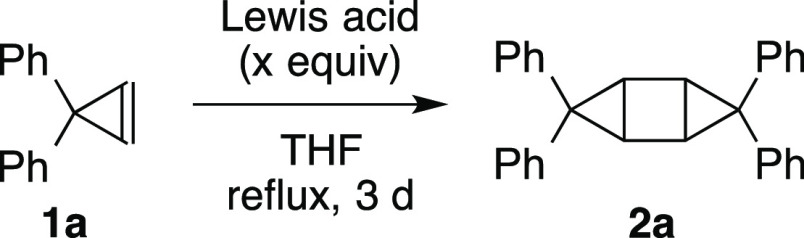
Screening of Lewis Acids

entry	Lewis acid (*x*)	NMR yield (%)
1^a^	Me_3_Al (1)	15
2	Me_3_Al (1)	92 (87)^b^
3^c^	Me_3_Al (0.5)	35
4	Me_3_Al (0.05)	26
5	Et_3_Al (1)	47
6	Me_2_AlCl (1)	35
7	BF_3_·OEt_2_ (1)	messy
8	Et_2_Zn (1)	not detected
9	AlCl_3_ (0.1)	no reaction
10	TiCl_4_ (0.1)	no reaction
11	Sc(OTf)_3_ (0.1)	no reaction
12	Yb(OTf)_3_ (0.1)	no reaction
13	NiCl_2_(dppe) (0.1)	messy
14	[RhCl(cod)]_2_ (0.05)	messy
15	Pd_2_(dba)_3_ (0.05)	messy
16	Al_2_O_3_ (0.05)	no reaction

a The reaction was conducted at room temperature for 40 h.

b Isolated yield is described in parentheses.

c The reaction time was 11 days.

**Figure 2:**
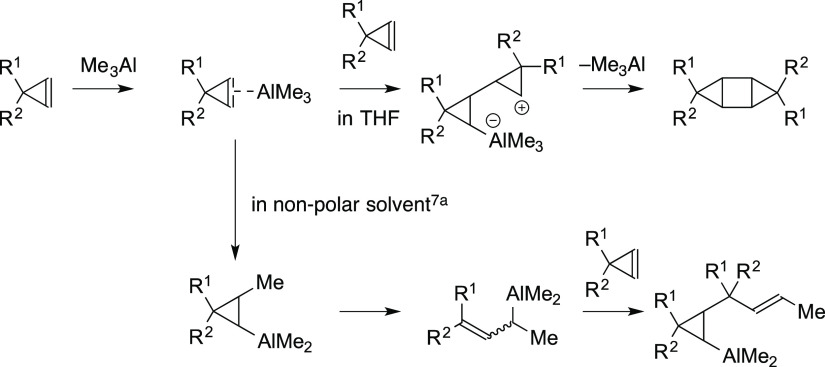
Speculated mechanism of the [2 + 2]-type reaction.

The optimized reaction conditions for various cyclopropenes gave tricyclo[3.1.0.0^2,4^]hexane derivatives (Table 2). The reaction of **1a** (2 mmol scale) gave the desired product **2a** in a 92% yield (entry 1). The reaction of diaryl-substituted cyclopropenes **1b**–**f** gave the desired products **2b**–**f** in moderate to high yields (entries 2–6, respectively). In some cases, the use of an excess amount of Me_3_Al was effective. Cyclopropenes bearing 2*-*substituted aryl groups inhibited the Me_3_Al-mediated reaction. The use of cyclopropenes bearing 3-substituted aryl groups gave the desired products, but a lack of reproducibility was observed. The reaction of dialkyl-substituted cyclopropenes **1g** and **1h** took place, but the yields were moderate (entries 7 and 8, respectively). The alkyl- and aryl-substituted cyclopropenes **1i**–**k** gave the desired products **2i**–**k** in low yields (entries 9–11, respectively). The stereochemistry of isolated **2i**–**k** would be *trans* according to the NMR spectra; *cis*-adducts might be removed during the purification (see the Supporting Information for details). The low yields of **2g**–**k** in the present dimerization reaction might be attributed to the Me_3_Al-mediated ring opening reaction of cyclopropenes in nonpolar solvents.^4,7^ We wondered if the ring-opening reaction of the cyclobutane moiety in the tricyclo[3.1.0.0^2,4^]hexane architecture would take place via the simple heating of tricyclo[3.1.0.0^2,4^]hexane derivatives. Typically, the ring-opening reaction of cyclobutane is thermochemically forbidden but photochemically allowed.^8^ The thermal ring-opening reaction of cyclobutane derivatives takes place via biradical intermediates, where simple cyclobutane requires ca. 1200 K to generate ethylene. In contrast, the ring-opening reaction of vinyl cyclobutanes takes place at ca. 400 K via allylic radical intermediates.^9^ We found that the thermal reactions of strained molecules **2a**–**k** at their respective temperatures gave the desired 1,4-cyclohexadiene derivatives **3a**–**k**. The thermal ring opening of *trans*-**2i**–**k** would give *trans*-**3i**–**k**, respectively, and the stereocenters at the benzylic positions were untouched during the reaction. The report concerning the Birch reduction of *p*-terphenyl shows that at room temperature *trans-*1,4-cyclohexadienes are typically solids and *cis*-1,4-cyclohexadienes are typically oils.^10^ The melting points of **3i**–**k** are higher than 140 °C, suggesting the generation of *trans*-**2** and *trans*-**3**.

**Table 2:**
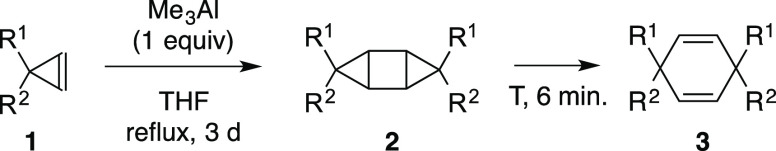
Synthesis of 1,4-Cyclohexadienes via Tricyclo[3.1.0.0^2,4^]hexanes

entry	R,^1^ R^2^	product **2** (%)	*T* (°C)	product **3** (%)
1	Ph (**1a**)	**2a**, 92	270	**3a**, 99
2	*p*-MeC_6_H_4_– (**1b**)	**2b**, 57 (66)^a^	245	**3b**, 98
3	*p*-MeOC_6_H_4_– (**1c**)	**2c**, 76	245	**3c**, 99
4	*p*-FC_6_H_4_– (**1d**)	**2d**, 90	258^c^	**3d**, 99
5	*p*-ClC_6_H_4_– (**1e**)	**2e**, 80	267	**3e**, 99
6	*p*-BrC_6_H_4_– (**1f**)	**2f**, 77	265	**3f**, 98
7	C_6_H_12_–, C_6_H_12_– (**1g**)	**2g**, 58	250^c^	**3g**, 98
8	–(CH_2_)_11_– (**1h**)	**2h**, 37	273	**3h**, 99
9	*p*-MeOC_6_H_4_–, Me (**1i**)	**2i**, 6 (14)^b^	221	**3i**, 99
10	*p*-ClC_6_H_4_–, Me (**1j**)	**2j**, 28 (39)^b^	230^d^	**3j**, 98
11	2-naphthyl, Me (**1k**)	**2k**, 40	225^c^	**3k**, 99

a Me_3_Al (1.1 equiv) was used.

b Me_3_Al (1.4 equiv) was used.

c The reaction time was 10 min.

d The reaction time was 30 min.

The oxidative rearrangement of cyclohexadienes would be a promising approach to the selective synthesis of 1,2,4,5-tetraarylbenzene derivatives. We considered that the Br_2_-mediated 1,2-rearrangement of an aryl group in alkenes may be a trigger to achieve the synthesis of 1,2,4,5-tetraarylbenzene derivatives.^11,12^ In a similar approach, the Br_2_-mediated oxidation of 1,4-cyclohexadienes was reported by DeBoer.^6^ We found that treating cyclohexadiene **3a** with Br_2_ (2 equiv) in CCl_4_ afforded the desired product **4a** in a 96% yield. The synthesis of 1,2,4,5-tetraarylbenzene derivatives **4** is shown (Table 3). The reactions of tetraarylcyclohexadienes **4b**, **4d**, **4e**, and **4f** using Br_2_ (2 equiv) in CCl_4_ from 0 °C to rt gave the desired products in excellent yields (entries 2–5, respectively). However, the reactions of alkyl-substituted cyclohexadienes **3g**, **3h**, and **3i** under similar conditions gave complicated unidentified products (entries 6–8, respectively). In the case of electron-rich cyclohexadiene **3c**, oxidative rearrangement and bromination occurred to give tetraarylbenzene **4c** in a 62% yield (Scheme 1). Several experiments performed using a stoichiometric amount of Br_2_ for the present oxidative rearrangement did not exhibit reproducibility. In contrast, the long reaction time for the reaction of **3a** in an excess amount of Br_2_ generated **4f**. We wondered if the bromination reaction of tetraarylbenzene derivatives would take place. The reaction of 1,2,4,5-tetra(*p*-methoxyphenyl)benzene and Br_2_ in CCl_4_ did not occur at all, but the simple bromination of tetraphenylbenzene using Br_2_ (15 equiv) without solvent under dark conditions was described previously.^2a^ The present conditions for oxidative rearrangement require an appropriate amount of Br_2_ and diluted conditions in CCl_4_; thus, the subsequent bromination of tetraarylbenzene derivatives under the present reaction conditions would be slow. The reason for the oxidative rearrangement of **3c** can give **4c** is unclear at the present stage.

**Table 3:**
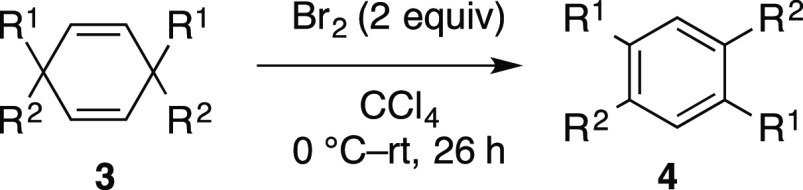
Oxidative Rearrangement of Cyclohexadienes

entry	R^1^, R^2^	product **4** (%)
1	Ph (**3a**)	**4a**, 98
2	*p*-MeC_6_H_4_– (**3b**)	**4b**, 98
3	*p*-FC_6_H_4_– (**3d**)	**4d**, 96
4	*p*-ClC_6_H_4_– (**3e**)	**4e**, 99
5	*p*-BrC_6_H_4_– (**3f**)	**4f**, 96
6	C_6_H_12_–, C_6_H_12_– (**3g**)	messy
7	*p*-ClC_6_H_4_–, Me (**3h**)	messy
8	2-naphthyl, Me (**3i**)	messy

**Scheme 1:**
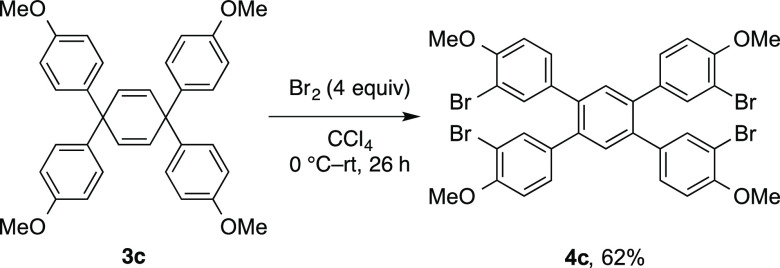
Oxidative Rearrangement of **3c**

The proposed mechanism for the oxidative rearrangement is shown in Figure 3. The generation of a bromonium intermediate **A** promotes the rearrangement of an aryl group to give intermediate **B**, followed by the rearrangement of a second aryl group to give intermediate **C**. The subsequent dehydrobromination of intermediates **C** and **D** resulted in the generation of the desired 1,2,4,5-tetraarylbenzene derivatives. We performed the reaction of 1,4-cyclohexadiene **3a** using TfOH or other Brønsted acids, but a complicated mixture was obtained.^13^

**Figure 3:**
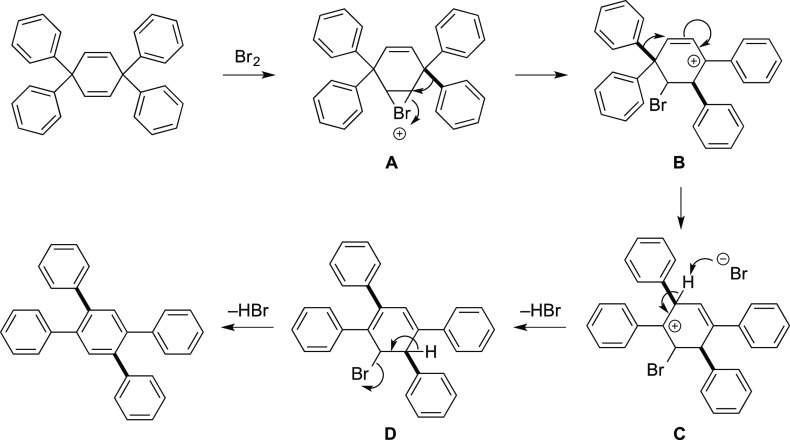
Proposed mechanism for dehydrogenative rearrangement.

We achieved the sequential transformation of cyclopropenes to 1,2,4,5-tetraarylbenzenes via a simple operation. The [2 + 2]-type dimerization of cyclopropenes took place in the presence of Me_3_Al to give tricyclo[3.1.0.0^2,4^]hexane derivatives, which were readily converted into 1,4-cyclohexadienes in almost quantitative yields via simple heating. Further novel oxidative rearrangement using Br_2_ led to the generation of 1,2,4,5-tetraarylbenzenes. The present approach to the synthesis of 1,2,4,5-tetraarylbenzenes contributes to the further synthesis of π-extended molecules.

## Experimental Section

### General Method

All reactions dealing with air-, moisture-, and light-sensitive compounds were carried out in a dry reaction vessel covered with foil under a positive pressure of argon. All liquids and solutions were transferred via a syringe. The reaction of compounds **1** under heating conditions was carried out using an oil bath. The reaction of compounds **2** was carried out in a crucible using an electric furnace. Analytical thin-layer chromatography was performed on an aluminum plate coated with silica gel containing a fluorescent indicator (Silica Gel 60 F254, Merck). The plates were visualized by exposure to ultraviolet light (254 nm) or by immersion in a basic staining solution of KMnO_4_, followed by heating with a heat gun. Organic solutions were concentrated using a rotary evaporator. Flash column chromatography was performed using Kanto Silica gel 60N (spherical, neutral). Proton nuclear magnetic resonance (^1^H NMR, 400 MHz), carbon nuclear magnetic resonance (^13^C{^1^H} NMR, 100 MHz), and fluorine nuclear magnetic resonance (^19^F NMR, 375 MHz) spectra were recorded with JEOL JNM-ECZ400S spectrometers. ^1^H NMR spectra in CDCl_3_ were referenced internally to tetramethylsilane (δ 0.00 ppm) as a standard, and ^13^C{^1^H} NMR spectra were referenced to the solvent resonance (CDCl_3_ δ 77.0 ppm). ^19^F NMR spectra in CDCl_3_ were referenced externally to trifluoroacetic acid (δ −76.5 ppm) as a standard. Data are presented as follows: chemical shift, spin multiplicity (s = singlet, d = doublet, t = triplet, q = quartet, and m = multiplet), coupling constant in hertz, and signal area integration in natural numbers. Cyclopropenes **1a**,^14a,14b^**1b**,^14a,14b^**1c**,^14b^**1d**,^14b^**1e**,^14a^**1f**,^14b^**1g**,^14c^**1h**,^14d^**1i**,^14e^**1j**,^14d^ and **1k**(14b,14d) were prepared according to reported procedures. The slow decomposition of some cyclopropenes was observed even in a refrigerator; thus, cyclopropenes should be used up within a few days.

### 3,3,6,6-Tetraphenyltricyclo[3.1.0.0^2,4^]hexane (**2a**)^5c^

To a solution of **1a** (38.0 mg 0.198 mmol) in THF (0.5 mL) was added 1.4 M Me_3_Al in hexane (0.14 mL, 0.196 mmol, 1 equiv) at 0 °C. The mixture was stirred at 70 °C for 3 d. The reaction was stopped with 1 M HCl aq. The aqueous phase was extracted with CHCl_3_. The combined organic layer was concentrated under vacuum. The residue was treated with hexane to give a white solid, which was collected by vacuum filtration in an 87% yield (33.0 mg, 0.086 mmol). For the 2 mmol scale reaction, to a solution of **1a** (385 mg, 2 mmol) in THF (10 mL) was added 1.4 M Me_3_Al in hexane (1.4 mL, 1.96 mmol, 1 equiv) at 0 °C. The mixture was stirred at 70 °C for 3 d. The reaction was stopped with 1 M HCl aq. The aqueous phase was extracted with CHCl_3_. The combined organic layer was concentrated under vacuum. The residue was treated with hexane to give a white solid, which was collected by vacuum filtration in a 92% yield (354 mg, 0.92 mmol): white solid; mp 264–265 °C; ^1^H NMR (400 MHz, CDCl_3_) δ 7.52–7.50 (m, 4H), 7.40–7.36 (m, 4H), 7.27–7.26 (m, 2H), 7.12–7.02 (m, 6H), 6.93–6.91 (m, 4H), 1.99 (s, 4H); ^13^C{^1^H} NMR (100 MHz, CDCl_3_) δ 143.6, 140.6, 130.7, 128.5, 128.2, 127.3, 126.5, 126.2, 55.8, 32.7; HRMS-APCI-TOF (*m*/*z*, positive) calcd for C_30_H_25_^+^ [M + H]^+^ 385.1951, found 385.1923.

### 3,3,6,6-Tetra-*p*-tolyltricyclo[3.1.0.0^2,4^]hexane (**2b**)

To a solution of **1b** (660 mg, 3.0 mmol) in THF (7.5 mL) was added 1.4 M Me_3_Al in hexane (2.4 mL, 3.3 mmol, 1.1 equiv) at 0 °C. The mixture was stirred at 70 °C for 3 d. The reaction was stopped with 1 M HCl aq. The aqueous phase was extracted with CHCl_3_. The combined organic layer was concentrated under vacuum. The residue was treated with hexane to give a white solid, which was collected by vacuum filtration in a 66% yield (434 mg, 0.983 mmol): white solid; mp 239–240 °C; ^1^H NMR (400 MHz, CDCl_3_) δ 7.36–7.34 (m, 4H), 7.12–7.10 (m, 4H), 6.89–6.83 (m, 8H), 2.31 (s, 6H), 2.17 (s, 6H), 1.91 (s, 4H); ^13^C{^1^H} NMR (100 MHz, CDCl_3_) δ 141.1, 137.9, 135.7, 135.6, 130.4, 129.1, 128.8, 127.4, 55.2, 32.3, 21.2, 20.9; HRMS-APCI-TOF (*m*/*z*, positive) calcd for C_34_H_33_^+^ [M + H]^+^ 441.2577, found 441.2580.

### 3,3,6,6-Tetrakis(4-methoxyphenyl)tricyclo[3.1.0.0^2,4^]hexane (**2c**)

To a solution of **1c** (86.0 mg, 0.341 mmol) in THF (0.85 mL) was added 1.4 M Me_3_Al in hexane (0.25 mL, 0.350 mmol, 1 equiv) at 0 °C. The mixture was stirred at 70 °C for 3 d. The reaction was stopped with 1 M HCl aq. The aqueous phase was extracted with CHCl_3_. The combined organic layer was concentrated under vacuum. The residue was treated with hexane to give a white solid, which was collected by vacuum filtration in a 76% yield (65.0 mg, 0.129 mmol): white solid; mp 234–235 °C; ^1^H NMR (400 MHz, CDCl_3_) δ 7.39–7.36 (m, 4H), 6.89–6.82 (m, 8H), 6.64–6.62 (m, 4H), 3.80 (s, 6H), 3.67 (s, 6H), 1.87 (s, 4H); ^13^C{^1^H} NMR (100 MHz, CDCl_3_) δ 158.1, 157.8, 136.4, 133.3, 131.4, 128.2, 113.9, 113.5, 55.30, 55.27, 54.3, 32.5; HRMS-APCI-TOF (*m*/*z*, positive) calcd for C_34_H_33_O_4_^+^ [M + H]^+^ 505.2373, found 505.2402.

### 3,3,6,6-Tetrakis(4-fluorophenyl)tricyclo[3.1.0.0^2,4^]hexane (**2d**)

To a solution of **1d** (256 mg, 1.12 mmol) in THF (2.8 mL) was added 1.4 M Me_3_Al in hexane (0.8 mL, 1.12 mmol, 1 equiv) at 0 °C. The mixture was stirred at 70 °C for 3 d. The reaction was stopped with 1 M HCl aq. The aqueous phase was extracted with CHCl_3_. The combined organic layer was concentrated under vacuum. The residue was treated with hexane to give a white solid, which was collected by vacuum filtration in a 90% yield (230 mg, 0.50 mmol): white solid; mp 240–241 °C; ^1^H NMR (400 MHz, CDCl_3_) δ 7.45–7.41 (m, 4H), 7.09–7.03 (m, 4H), 6.83–6.79 (m, 8H), 1.89 (s, 4H); ^13^C{^1^H} NMR (100 MHz, CDCl_3_) δ 161.5 (*J*_C–F_ = 245 Hz), 161.3 (*J*_C–F_ = 244 Hz), 138.8 (*J*_C–F_ = 29.0 Hz), 135.9 (*J*_C–F_ = 28.0 Hz), 131.8 (*J*_C–F_ = 7.6 Hz), 128.5 (*J*_C–F_ = 8.7 Hz), 115.6 (*J*_C–F_ = 21.1 Hz), 115.1 (*J*_C–F_ = 21.1 Hz), 54.4, 32.7; ^19^F NMR (375 MHz, CDCl_3_) δ −115.7, −116.3; HRMS-APCI-TOF (*m*/*z*, positive) calcd for C_30_H_21_F_4_^+^ [M + H]^+^ 457.1574, found 457.1597.

### 3,3,6,6-Tetrakis(4-chlorophenyl)tricyclo[3.1.0.0^2,4^]hexane (**2e**)

To a solution of **1e** (52.0 mg, 0.2 mmol) in THF (0.5 mL) was added 1.4 M Me_3_Al in hexane (0.14 mL, 0.196 mmol, 1 equiv) at 0 °C. The mixture was stirred at 70 °C for 3 d. The reaction was stopped with 1 M HCl aq. The aqueous phase was extracted with CHCl_3_. The combined organic layer was concentrated under vacuum. The residue was treated with hexane to give a white solid, which was collected by vacuum filtration in an 80% yield (42.0 mg, 0.08 mmol): white solid; mp 262–263 °C; ^1^H NMR (400 MHz, CDCl_3_) δ 7.39–7.33 (m, 8H), 7.08–7.06 (m, 4H), 6.77–6.75 (m, 4H) 1.91 (s, 4H); ^13^C{^1^H} NMR (100 MHz, CDCl_3_) δ 141.1, 138.3, 132.7, 132.3, 131.9, 129.0, 128.5, 128.3, 54.7, 32.8; HRMS-APCI-TOF (*m*/*z*, positive) calcd for C_30_H_20_Cl_4_^+^ [M]^+^ 520.0314, found 520.0321.

### 3,3,6,6-Tetrakis(4-bromophenyl)tricyclo[3.1.0.0^2,4^]hexane (**2f**)

To a solution of **1f** (273 mg, 0.78 mmol) in THF (2 mL) was added 1.4 M Me_3_Al in hexane (0.56 mL, 0.78 mmol, 1 equiv) at 0 °C. The mixture was stirred at 70 °C for 3 d. The reaction was stopped with 1 M HCl aq. The aqueous phase was extracted with CHCl_3_. The combined organic layer was concentrated under vacuum. The residue was treated with hexane to give a white solid, which was collected by vacuum filtration in a 77% yield (210 mg, 0.30 mmol): white solid; mp 259–260 °C; ^1^H NMR (400 MHz, CDCl_3_) δ 7.52–7.50 (m, 4H), 7.34–7.32 (m, 4H), 7.25–7.23 (m, 4H), 6.72–6.71 (m, 4H), 1.92 (s, 4H); ^13^C{^1^H} NMR (100 MHz, CDCl_3_) δ 141.5, 138.7, 132.2, 131.9, 131.4, 128.7, 120.9, 120.4, 54.9, 32.8; HRMS-APCI-TOF (*m*/*z*, positive) calcd for C_30_H_20_Br_4_^+^ [M]^+^ 695.8293, found 695.8299.

### 3,3,6,6-Tetracyclohexyltricyclo[3.1.0.0^2,4^]hexane (**2g**)

To a solution of **1g** (447 mg, 2.19 mmol) in THF (5.5 mL) was added 1.4 M Me_3_Al in hexane (1.56 mL, 2.19 mmol, 1 equiv) at 0 °C. The mixture was stirred at 70 °C for 3 d. The reaction was stopped with 1 M HCl aq. The aqueous phase was extracted with CHCl_3_. The combined organic layer was concentrated under vacuum. Recrystallization in CHCl_3_ and hexane gave a desired product in a 58% yield (260 mg, 0.64 mmol): white solid; mp 239–240 °C; ^1^H NMR (400 MHz, CDCl_3_); δ 1.79–0.80 (m, 48H); ^13^C{^1^H} NMR (100 MHz, CDCl_3_) δ 53.1, 37.9, 32.6, 31.7, 29.7, 27.7, 27.0, 26.8, 26.4 (1C overlapping); HRMS-APCI-TOF (*m*/*z*, positive) calcd for C_30_H_49_^+^ [M + H]^+^ 409.3829, found 409.3847.

### Dispiro[cyclododecane-1,3′-tricyclo[3.1.0.0^2,4^]hexane-6′,1″-cyclododecane] (**2h**)

To a solution of **1h** (414 mg, 2.15 mmol) in THF (5.4 mL) was added 1.4 M Me_3_Al in hexane (1.54 mL, 2.15 mmol, 1 equiv) at 0 °C. The mixture was stirred at 70 °C for 3 d. The reaction was stopped with 1 M HCl aq. The aqueous phase was extracted with CHCl_3_. The combined organic layer was concentrated under vacuum. Recrystallization in CHCl_3_ and hexane gave a desired product in a 37% yield (151 mg, 0.393 mmol): white solid; mp 222–223 °C; ^1^H NMR (400 MHz, CDCl_3_) δ 1.62–1.55 (m, 12H), 1.48–1.25 (m, 28H), 1.10 (s, 4H), 1.03–1.00 (m, 4H); ^13^C{^1^H} NMR (100 MHz, CDCl_3_) δ 43.8, 30.4, 26.8, 26.5, 26.45, 26.41, 22.3, 22.2, 21.8, 21.4 (3C overlapping); HRMS-APCI-TOF (*m*/*z*, positive) calcd for C_28_H_49_^+^ [M + H]^+^ 385.3829, Found 385.3821.

### 3,6-Bis(4-methoxyphenyl)-3,6-dimethyltricyclo[3.1.0.0^2,4^]hexane (**2i**)

To a solution of **1i** (152 mg, 0.95 mmol) in THF (3.3 mL) was added 1.4 M Me_3_Al in hexane (0.95 mL, 1.33 mmol, 1.4 equiv) at 0 °C. The mixture was stirred at 70 °C for 3 d. The reaction was stopped with 1 M HCl aq. The aqueous phase was extracted with CHCl_3_. The combined organic layer was concentrated under vacuum. Purification by silica gel column chromatography using CHCl_3_ as an eluent gave a desired product in a 14% yield (21.3 mg, 0.0666 mmol): white solid; mp 205–206 °C; ^1^H NMR (400 MHz, CDCl_3_) δ 7.20–7.17 (m, 4 H), 6.83–6.81 (m, 4 H), 3.78 (s, 6 H), 1.77 (s, 4H), 1.55 (s, 6 H); ^13^C{^1^H} NMR (100 MHz, CDCl_3_) δ 157.9, 137.9, 128.8, 113.7, 55.3, 44.3, 28.4, 17.0; HRMS-APCI-TOF (*m*/*z*, positive) calcd for C_22_H_25_O_2_^+^ [M + H]^+^ 321.1849, found 321.1841.

### 3,6-Bis(4-chlorophenyl)-3,6-dimethyltricyclo[3.1.0.0^2,4^]hexane (**2j**)

To a solution of **1j** (278 mg, 1.69 mmol) in THF (6.0 mL) was added 1.4 M Me_3_Al in hexane (1.7 mL, 2.38 mmol, 1.4 equiv) at 0 °C. The mixture was stirred at 70 °C for 3 d. The reaction was stopped with 1 M HCl aq. The aqueous phase was extracted with CHCl_3_. The combined organic layer was concentrated under vacuum. Purification by silica gel column chromatography using CHCl_3_ as an eluent gave a desired product in a 39% yield (109 mg, 0.33 mmol): white solid; mp 170–171 °C; ^1^H NMR (400 MHz CDCl_3_) δ 7.25–7.23 (m, 4H), 7.19–7.17 (m, 4H), 1.80 (s, 4H), 1.58 (s, 6H); ^13^C{^1^H} NMR (100 MHz, CDCl_3_) δ 143.9, 131.9, 128.9, 128.4, 44.3, 28.7, 16.5; HRMS-APCI-TOF (*m*/*z*, positive) calcd for C_20_H_19_Cl_2_^+^ [M + H]^+^ 329.0858, found 329.0872.

### 3,6-Dimethyl-3,6-di(napthalen-2-yl)tricyclo[3.1.0.0^2,4^]hexane (**2k**)

To a solution of **1k** (324 mg, 1.80 mmol) in THF (4.5 mL) was added 1.4 M Me_3_Al in hexane (1.28 mL, 1.80 mmol, 1 equiv) at 0 °C. The mixture was stirred at 70 °C for 3 d. The reaction was stopped with 1 M HCl aq. The aqueous phase was extracted with CHCl_3_. The combined organic layer was concentrated under vacuum. Recrystallization in CHCl_3_ and hexane gave a desired product in a 40% yield (128 mg, 0.36 mmol): white solid; mp 216–217 °C; ^1^H NMR (400 MHz, CDCl_3_) δ 7.81–7.74 (m, 8H), 7.47–7.41 (m, 6H), 2.01 (s, 4H), 1.72 (s, 6H); ^13^C{^1^H} NMR (100 MHz, CDCl_3_) δ 143.0, 133.5, 132.2, 127.9, 127.8, 127.6, 126.4, 126.0, 125.5, 45.1, 28.7, 16.7 (1C overlapping); HRMS-APCI-TOF (*m*/*z*, positive) calcd for C_28_H_25_^+^ [M + H] ^+^ 361.1951, found 361.1976.

### 1′,4′-Diphenyl-1′,4′-dihydro-1,1′:4′,1″-terphenyl (**3a**)

**2a** (59.0 mg, 0.154 mmol) was heated at 270 °C for 6 min to give the desired product in a 99% yield (58.7 mg, 0.152 mmol): yellow solid; mp 235–236 °C; ^1^H NMR (400 MHz, CDCl_3_) δ 7.31–7.19 (m, 20H), 6.15 (s, 4H); ^13^C{^1^H} NMR (100 MHz, CDCl_3_) δ 147.2, 131.0, 128.4, 128.2, 126.3, 50.1; HRMS-APCI-TOF (*m*/z, positive) calcd for C_30_H_25_^+^ [M + H]^+^ 385.1951, found 385.1928.

### 4,4″-Dimethyl-4′,4′-di-*p*-tolyl-4′H-1,1′:1′,1″-terphenyl (**3b**)

**2b** (99.8 mg, 0.226 mmol) was heated at 245 °C for 6 min to give the desired product in a 98% yield (97.8 mg, 0.222 mmol): yellow solid; mp 262–263 °C; ^1^H NMR (400 MHz, CDCl_3_) δ 7.02 (broad s, 16H), 6.00 (s, 4H), 2.25 (s, 12H); ^13^C{^1^H} NMR (100 MHz, CDCl_3_) δ 144.5, 135.8, 130.9, 129.0, 128.1, 49.4, 21.1; HRMS-APCI-TOF (*m*/*z*, positive) calcd for C_34_H_33_^+^ [M + H]^+^, 441.2577, found 441.2557.

### 4,4″-Dimethoxy-4′,4′-bis(4-methoxyphenyl)-4′H-1,1′:1′-1″-terphenyl (**3c**)

**2c** (98.7 mg, 0.196 mmol) was heated at 245 °C for 6 min to give the desired product in a 99% yield (98.0 mg, 0.194 mmol): yellow solid; mp 183–184 °C; ^1^H NMR (400 MHz, CDCl_3_) δ 7.14–7.12 (m, 8H), 6.83–6.79 (m, 8H), 6.05 (s, 4H), 3.79 (s, 12H); ^13^C{^1^H} NMR (100 MHz, CDCl_3_) δ 157.9, 139.6, 130.9, 129.1, 113.7, 55.3, 48.6; HRMS-APCI-TOF (*m*/*z*, positive) calcd for C_34_H_33_O_4_^+^ [M + H]^+^ 505.2373, found 505.2370.

### 4,4″-Difluoro-4′,4′-bis(4-fluorophenyl)-4′H-1,1′:1′,1″-terphenyl (**3d**)

**2d** (71.6 mg, 0.157 mmol) as heated at 258 °C for 10 min to give the desired product in a 99% yield (71.1 mg, 0.156 mmol): yellow solid; mp 220–221 °C; ^1^H NMR (400 MHz, CDCl_3_) δ 7.14–7.11 (m, 8H), 6.98–6.95 (m, 8H), 6.08 (s, 4H); ^13^C{^1^H} NMR (100 MHz, CDCl_3_) δ 161.5 (*J*_C–F_ = 245 Hz), 142.4 (*J*_C–F_ = 27 Hz), 130.9, 129.5 (*J*_C–F_ = 7.7 Hz), 115.3 (*J*_C–F_ = 21.2 Hz), 48.8; ^19^F NMR (375 MHz, CDCl_3_) δ −116.2; HRMS-APCI-TOF (*m*/*z*, positive) calcd for C_30_H_21_F_4_^+^ [M + H]^+^ 457.1574, found 457.1577.

### 4,4″-Dichloro-4′,4′-bis(4-chlorophenyl)-4′H-1,1′:1′,1″-terphenyl (**3e**)

**2e** (52.5 mg, 0.101 mmol) was heated at 267 °C for 6 min to give the desired product in a 99% yield (52.3 mg, 0.100 mmol): yellow solid; mp 267–268 °C; ^1^H NMR (400 MHz, CDCl_3_) δ 7.25–7.24 (m, 8H), 7.09–7.06 (m, 8H), 6.06 (s, 4H); ^13^C{^1^H} NMR (100 MHz, CDCl_3_) δ 144.8, 132.6, 130.8, 129.3, 128.8, 49.2; HRMS-APCI-TOF (*m*/*z*, positive) calcd for C_30_H_20_Cl_4_^+^ [M]^+^ 520.0314, found 520.0334.

### 4,4″-Dibromo-4′,4′-bis(4-bromophenyl)-4′H-1,1′:1′,1″-terphenyl (**3f**)

**2f** (70.3 mg, 0.101 mmol) was heated at 265 °C for 6 min to give the desired product in a 98% yield (69.6 mg, 0.099 mmol): yellow solid; mp 301–302 °C; ^1^H NMR (400 MHz, CDCl_3_) δ 7.42–7.39 (m, 8H), 7.02–7.00 (m, 8H), 6.05 (s, 4H); ^13^C{^1^H} NMR (100 MHz, CDCl_3_) δ 145.3, 131.8, 130.7, 129.7, 120.9, 49.3; HRMS-APCI-TOF (*m*/*z*, positive) calcd for C_30_H_20_Br_4_^+^ [M]^+^ 695.8293, found 695.8299.

### 1′,4′-Dicyclohexyl-[1,1′:4′,1″-tercyclohexane]-2′,5′-diene (**3g**)

**2g** (39.7 mg, 0.097 mmol) was heated at 250 °C for 10 min to give the desired product in a 98% yield (39.0 mg, 0.096 mmol): yellow solid; mp 153–154 °C; ^1^H NMR (400 MHz, CDCl_3_) δ 5.50 (s, 4H), 1.86–0.90 (m, 44H); ^13^C{^1^H} NMR (100 MHz, CDCl_3_) δ 131.7, 45.5, 44.7, 28.9, 27.5, 26.9; HRMS-APCI-TOF (*m*/*z*, positive) calcd for C_30_H_49_^+^ [M + H]^+^ 409.3829, found 409.3814.

### Dispiro[11.2.11^15^.2^12^]octacosa-13,27-diene (**3h**)

**2h** (12.1 mg, 0.031 mmol) was heated at 273 °C for 6 min to give the desired product in a 99% yield (11.9 mg, 0.031 mmol): white solid; mp 223–224 °C; ^1^H NMR (400 MHz, CDCl_3_) δ 5.50 (s, 4H), 1.49–1.23 (m, 44H); ^13^C{^1^H} NMR (100 MHz, CDCl_3_) δ 131.4, 38.6, 35.7, 26.9, 26.2, 22.8, 22.3, 18.9; HRMS-APCI-TOF (*m*/*z*, positive) calcd for C_28_H_49_^+^ [M + H]^+^ 385.3829, found 385.3851.

### 4,4″-Dimethoxy-1′,4′-dimethyl-1′,4′-dihydro-1,1′:4′,1″-terphenyl (**3i**)

**2i** (21.3 mg, 0.067 mmol) was heated at 221 °C for 6 min to give the desired product in a 99% yield (21.2 mg, 0.066 mmol): yellow solid; mp 163–164 °C; ^1^H NMR (400 MHz, CDCl_3_) δ 7.34–7.31 (m, 4H), 6.89–6.86 (m, 4H), 5.65 (s, 4H), 3.80 (s, 6 H), 1.54 (s, 6 H); ^13^C{^1^H} NMR (100 MHz, CDCl_3_) δ 157.9, 139.8, 131.8, 127.7, 113.7, 55.4, 39.8, 27.8; HRMS-APCI-TOF (*m*/*z*, positive) calcd for C_22_H_25_O_2_^+^ [M + H^+^] 321.1849, found 321.1831.

### 4,4″-Dichloro-1′,4′-dimethyl-1′,4′-dihydro-1,1′:4′,1″-terphenyl (**3j**)

**2j** (32.4 mg, 0.099 mmol) was heated at 230 °C for 30 min to give the desired products in a 98% yield (31.8 mg, 0.097 mmol): yellow solid; mp 144–145 °C; ^1^H NMR (400 MHz, CDCl_3_) δ 7.54–7.16 (m, 8H), 5.65 (s, 4H), 1.53 (s, 6 H); ^13^C{^1^H} NMR (100 MHz, CDCl_3_) δ 145.9, 132.1, 131.7, 128.5, 128.1, 40.2, 27.6; HRMS-APCI-TOF (*m*/*z*, negative) calcd for C_20_H_17_Cl_2_^–^ [M – H^–^] 327.0713, found 327.0700.

### 3,6-Dimethyl-3,6-di(napthalen-2-yl)cyclohexa-1,4-diene (**3k**)

**2k** (36.0 mg, 0.100 mmol) was heated at 225 °C for 10 min to give the desired product in a 99% yield (35.7 mg, 0.099 mmol): yellow solid; mp 201–202 °C; ^1^H NMR (400 MHz, CDCl_3_) δ 7.85–7.80 (m, 8H), 7.60–7.57 (m, 2H), 7.47–7.45 (m, 4H), 5.81 (s, 4H), 1.73 (s, 6H); ^13^C{^1^H} NMR (100 MHz, CDCl_3_) δ 144.8, 133.6, 132.1, 131.9, 128.1, 127.9, 127.6, 126.13, 126.07, 125.7, 124.3, 40.8, 27.7; HRMS-APCI-TOF (*m*/*z*, positive) calcd for C_28_H_25_^+^ [M + H] ^+^ 361.1951, found 361.1950.

### 4,5′-Diphenyl-1,1′:2′,1″-terphenyl (**4a**)^2b^

To a mixture of **3a** (38.9 mg, 0.101 mmol) in CCl_4_ (0.1 mL) was added Br_2_ (10 μL, 0.194 mmol, 2 equiv) in CCl_4_ (0.1 mL) at 0 °C. After 30 min of stirring at 0 °C, the reaction mixture was warmed to room temperature. The mixture was stirred at that temperature for 26 h. Then, to the mixture was added aq Na_2_S_2_O_3_. The aqueous phase was extracted with CH_2_Cl_2_. The combined organic layer was concentrated under vacuum. Purification by silica gel column chromatography using chloroform as an eluent gave a solid, which was washed with a minimum amount of EtOAc. The product was obtained in a 98% yield (37.7 mg, 0.098 mmol): ^1^H NMR (400 MHz, CDCl_3_) δ 7.51 (s, 2H), 7.25–7.21 (m, 20H); ^13^C{^1^H} NMR (100 MHz, CDCl_3_) δ 141.0, 139.7, 133.1, 129.9, 128.0, 126.7; HRMS-APCI-TOF (*m*/*z*, positive) calcd for C_30_H_23_^+^ [M + H] ^+^ 383.1794, found 383.1773.

### 4,4″-Dimethyl-4′,5′-bis(4-methylphenyl)-1,1′:2′,1″-terphenyl (**4b**)^2b^

To a mixture of **3b** (44.0 mg, 0.100 mmol) in CCl_4_ (0.1 mL) was added Br_2_ (10 μL, 0.194 mmol, 2 equiv) in CCl_4_ (0.1 mL) at 0 °C. After 30 min of stirring at 0 °C, the reaction mixture was warmed to room temperature. The mixture was stirred at that temperature for 26 h. Then, to the reaction mixture was added aq Na_2_S_2_O_3_. The aqueous phase was extracted with CH_2_Cl_2_. The combined organic layer was concentrated under vacuum. Purification by silica gel column chromatography using chloroform as an eluent gave a solid, which was washed with a minimum amount of EtOAc. The product was obtained in a 98% yield (43.1 mg, 0.098 mmol): ^1^H NMR (400 MHz, CDCl_3_) δ 7.39 (s, 2H), 7.05–6.95 (m, 16H), 2.24 (s, 12H); ^13^C{^1^H} NMR (100 MHz, CDCl_3_) δ 139.2, 138.2, 136.1, 133.0, 129.7, 128.7, 21.1; HRMS-APCI-TOF (*m*/*z*, positive) calcd for C_34_H_30_^+^ [M] ^+^ 438.2342, found 438.2335.

### 3,3″-Dibromo-4′,5′-bis(3-bromo-4-methoxyphenyl)-4,4″-dimethoxy-1,1′:2′,1″-terphenyl (**4c**)

To a mixture of **3c** (51.0 mg, 0.101 mmol) in CCl_4_ (0.1 mL) was added Br_2_ (20 μL, 0.388 mmol, 4 equiv) in CCl_4_ (0.1 mL) at 0 °C. After 30 min of stirring at 0 °C, the reaction mixture was warmed to room temperature. The mixture was stirred at that temperature for 26 h. Then, to the reaction mixture was added aq Na_2_S_2_O_3_. The aqueous phase was extracted with CH_2_Cl_2_. The combined organic layer was concentrated under vacuum. Purification by silica gel column chromatography using chloroform as an eluent gave a solid, which was washed with a minimum amount of EtOAc. The product was obtained in a 62% yield (51.0 mg, 0.063 mmol): white solid; mp 257–258 °C; ^1^H NMR (400 MHz, CDCl_3_) δ 7.49–7.48 (m, 4H), 7.37 (s, 2 H), 7.00–6.97 (m, 4H), 6.76–6.74 (m, 4H), 3.87 (s, 12H); ^13^C{^1^H} NMR (100 MHz, CDCl_3_) δ 154.9, 137.9, 134.19, 134.15, 132.6, 130.1, 111.4, 111.3, 56.2; HRMS-APCI-TOF (*m*/*z*, positive) calcd for C_34_H_26_Br_4_O_4_^+^ [M + H]^+^ 814.8637, found 814.8641.

### 4,4″-Difluoro-4′,5′-bis(4-fluorophenyl)-1,1′:2′,1″-terphenyl (**4d**)^2b^

To a mixture of **3d** (45.9 mg, 0.100 mmol) in CCl_4_ (0.1 mL) was added Br_2_ (10 μL, 0.194 mmol, 2 equiv) in CCl_4_ (0.1 mL) at 0 °C. After 30 min of stirring at 0 °C, the reaction mixture was warmed to room temperature. The mixture was stirred at that temperature for 26 h. Then, to the reaction mixture was added aq Na_2_S_2_O_3_. The aqueous phase was extracted with CH_2_Cl_2_. The combined organic layer was concentrated under vacuum. Purification by silica gel column chromatography using chloroform as an eluent gave a solid, which was washed with a minimum amount of EtOAc. The product was obtained in a 96% yield (43.7 mg, 0.096 mmol): ^1^H NMR (400 MHz, CDCl_3_) δ 7.42 (s, 2H), 7.16–7.12 (m, 8H), 6.96–6.92 (m, 8H); ^13^C{^1^H} NMR (100 MHz, CDCl_3_) δ 161.9 (d, *J*_C–F_ = 246 Hz), 138.8, 136.5 (d, *J*_C–F_ = 3.8 Hz), 132.8, 131.3 (d, *J*_C–F_ = 7.7 Hz), 115.1 (d, *J*_C–F_ = 21 Hz); ^19^F NMR (375 MHz, CDCl_3_) δ −115.4; HRMS-APCI-TOF (*m*/*z*, positive) calcd. for C_30_H_18_F_4_^+^ [M]^+^ 454.1339, found 454.1331.

### 4,4″-Dichloro-4′,5′-bis(4-chlorophenyl)-1,1′:2′,1″-terphenyl (**4e**)^2b^

To a mixture of **3e** (52.3 mg, 0.101 mmol) in CCl_4_ (0.1 mL) was added Br_2_ (10 μL, 0.194 mmol, 2 equiv) in CCl_4_ (0.1 mL) at 0 °C. After 30 min of stirring at 0 °C, the reaction mixture was warmed to room temperature. The mixture was stirred at that temperature for 26 h. Then, to the reaction mixture was added aq Na_2_S_2_O_3_. The aqueous phase was extracted with CH_2_Cl_2_. The combined organic layer was concentrated under vacuum. Purification by silica gel column chromatography using chloroform as an eluent gave a solid, which was washed with a minimum amount of EtOAc. The product was obtained in a 99% yield (51.9 mg, 0.100 mmol): ^1^H NMR (400 MHz, CDCl_3_) δ 7.42 (s, 2H), 7.25–7.22 (m, 8H), 7.12–7.10 (m, 8H); ^13^C{^1^H} NMR (100 MHz, CDCl_3_) δ 138.8, 138.7, 133.2, 132.7, 131.0, 128.5; HRMS-APCI-TOF (*m*/*z*, positive) calcd. for C_30_H_18_Cl_4_^+^ [M]^+^ 518.0157, found 518.0152.

### 4,4″-Dibromo-4′,5′-bis(4-bromophenyl)-1,1′:2′,1″-terphenyl (**4f**)^2a^

To a mixture of **3f** (70.0 mg, 0.100 mmol) in CCl_4_ (0.1 mL) was added Br_2_ (10 μL, 0.194 mmol, 2 equiv) in CCl_4_ (0.1 mL) at 0 °C. After 30 min of stirring at 0 °C, the reaction mixture was warmed to room temperature. The mixture was stirred at that temperature for 26 h. Then, to the reaction mixture was added aq Na_2_S_2_O_3_. The aqueous phase was extracted with CH_2_Cl_2_. The combined organic layer was concentrated under vacuum. Purification by silica gel column chromatography using chloroform as an eluent gave a solid, which was washed with a minimum amount of EtOAc. The product was obtained in a 96% yield (66.9 mg, 0.096 mmol): ^1^H NMR (400 MHz, CDCl_3_) δ 7.41–7.38 (m, 10H), 7.06–7.04 (m, 8H); ^13^C{^1^H} NMR (100 MHz, CDCl_3_) δ 139.2, 138.9, 132.8, 131.5, 131.4, 121.5; HRMS-APCI-TOF (*m*/*z*, positive) calcd. for C_30_H_19_Br_4_^+^ [M + H]^+^ 694.8215, found 694.8201.

## Supporting Information Available

NMR spectra and stereochemistry predictions for **2i**–**k** (PDF)
