# Supplementary material for: Cyclopropenes for the Stepwise Synthesis of 1,2,4,5-Tetraarylbenzenes via 1,4-Cyclohexadienes
Source: J Org Chem. 2022 Oct 6;87(21):14833–9. doi: 10.1021/acs.joc.2c01261 (PMC9639005; doi:10.1021/acs.joc.2c01261)
Supplement: Supplementary file 1 — jo2c01261_si_001.pdf [file jo2c01261_si_001.pdf]

Supporting Information

Cyclopropenes for the Stepwise Synthesis of 1,2,4,5-Tetraarylbenzenes via 1,4-Cyclohexadienes

Satoshi Kishida,[1] Misaki Takano,[1] Takuya Sekiya,[2] Yutaka Ukaji,[2] Kohei Endo[1]*

[1] Department of Chemistry, Faculty of Science, Tokyo University of Science, Tokyo 162-8601, Japan
[2] Division of Material Chemistry, Graduate School of Natural Science and Technology, Kanazawa University, Kakuma, Kanazawa, Ishikawa 920-1192, Japan

*Email: kendo@rs.tus.ac.jp

1	^{1}H, ^{13}C, ^{19}F NMR spectra of **2a**–**k**	S2
2	The prediction of stereochemistry for **2i**–**k**	S14
3	^{1}H, ^{13}C, ^{19}F NMR spectra of **3a**–**k**	S17
4	^{1}H, ^{13}C, ^{19}F NMR spectra of **4a**–**f**	S29
5	Reference	S36

2a: ^{1}H NMR (400 MHz, $CDCl_3$)

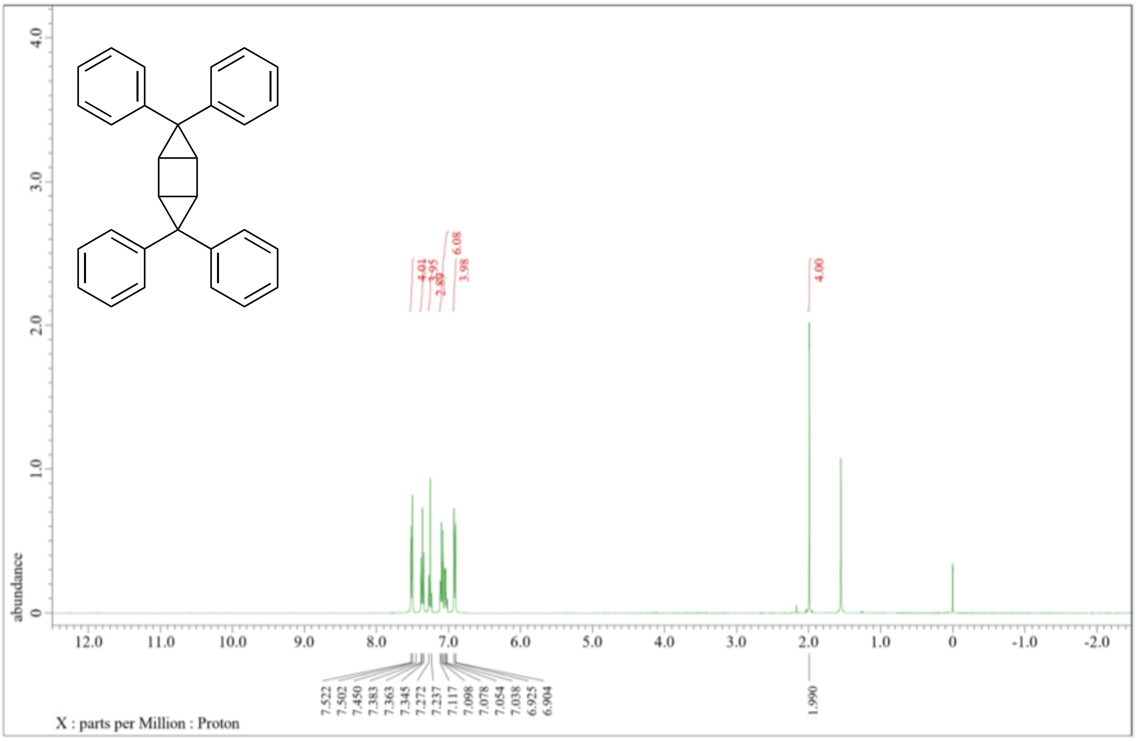

2a: ^{13}C{^{1}H} NMR (100 MHz, $CDCl_3$)

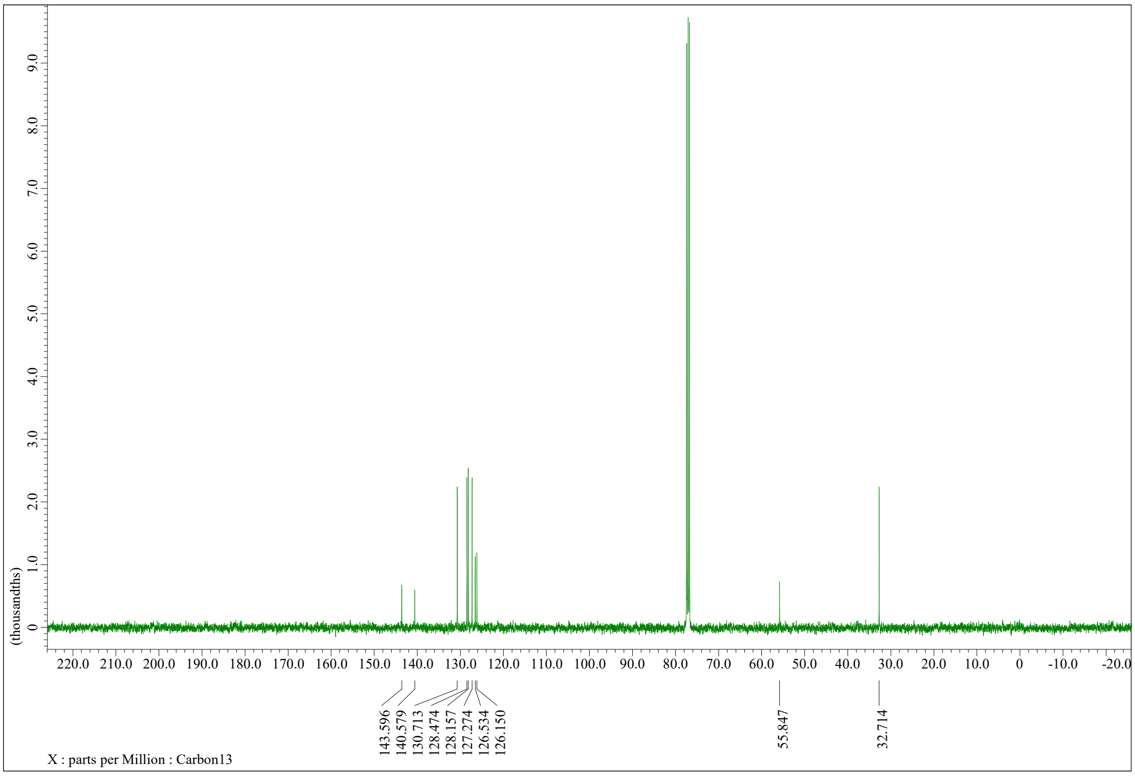

2b: ^{1}H NMR (400 MHz, $CDCl_3$)

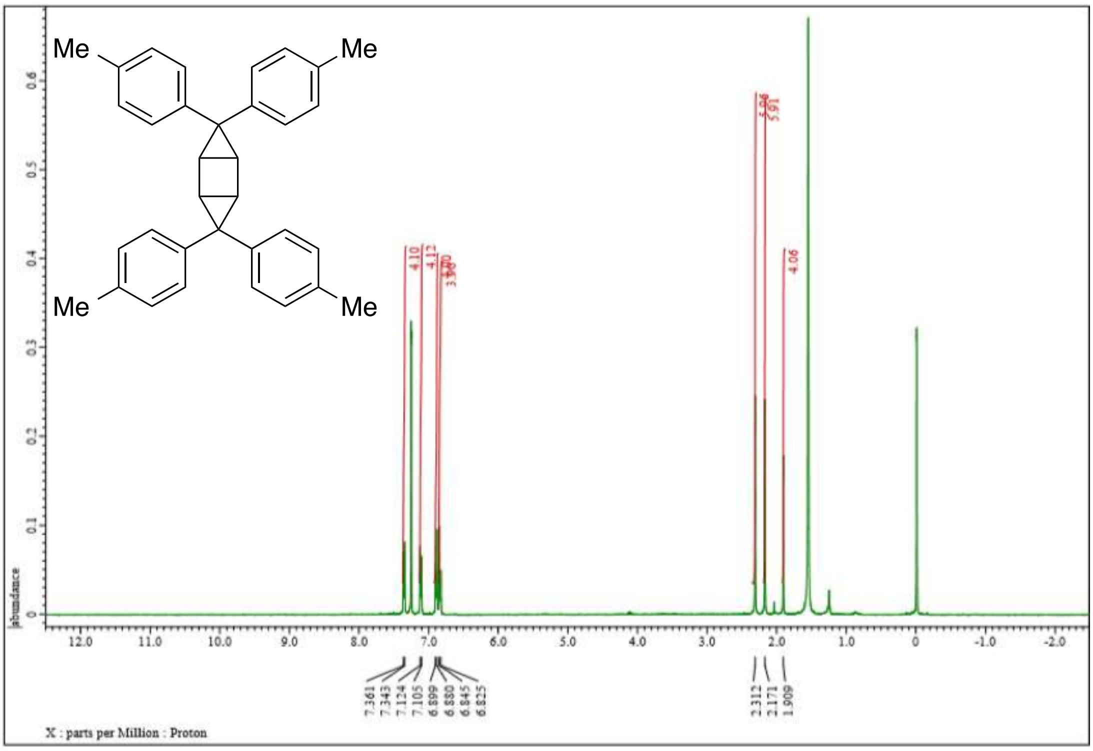

2b: $^{13}C\{^{1}H\}$ NMR (100 MHz, $CDCl_3$)

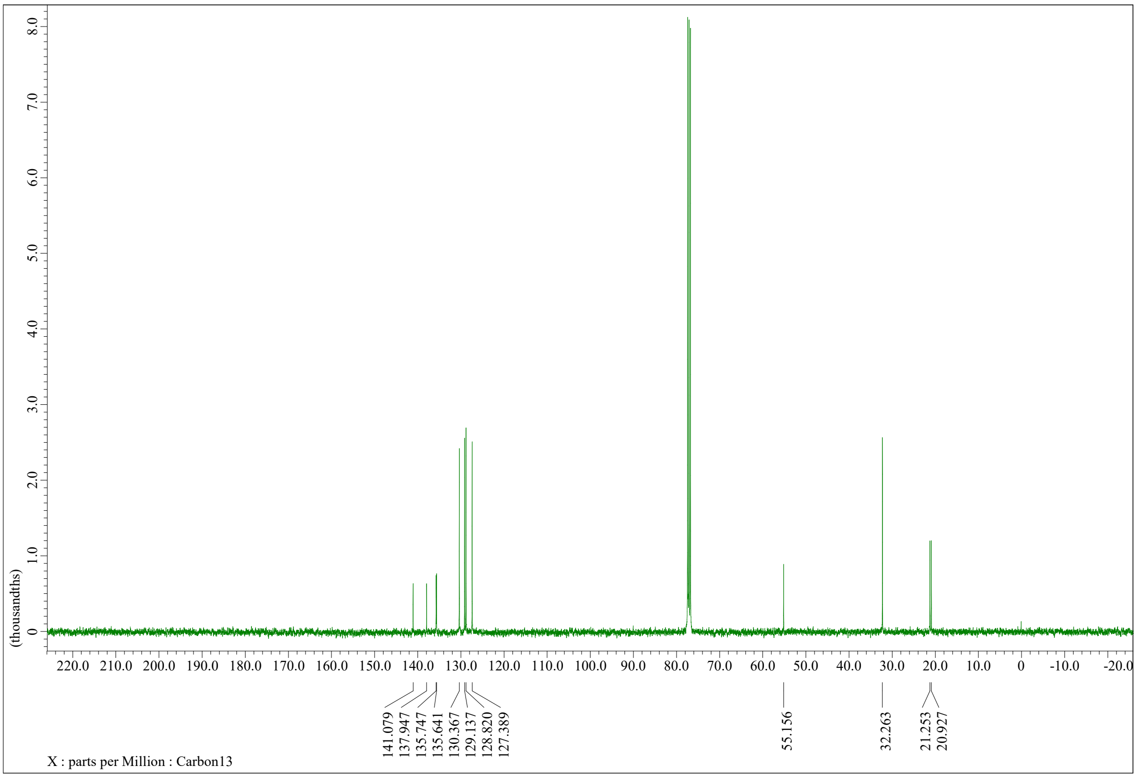

2c: ^{1}H NMR (400 MHz, $CDCl_3$)

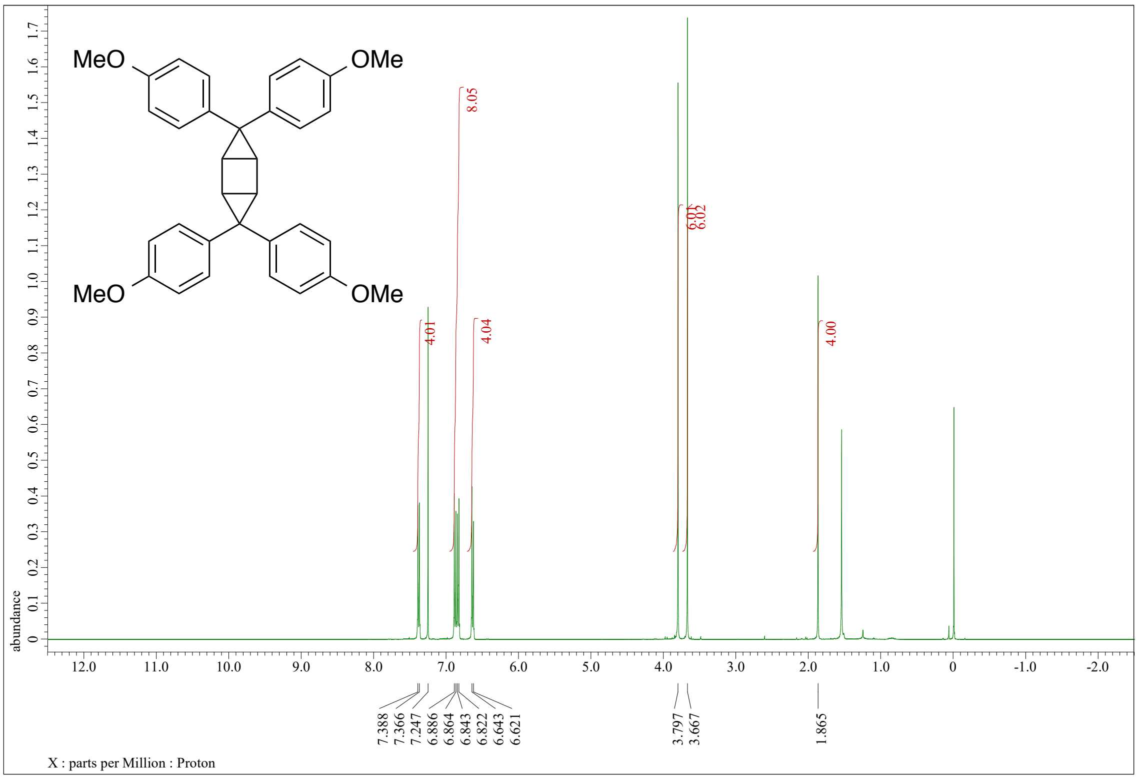

2c: $^{13}C\{^{1}H\}$ NMR (100 MHz, $CDCl_3$)

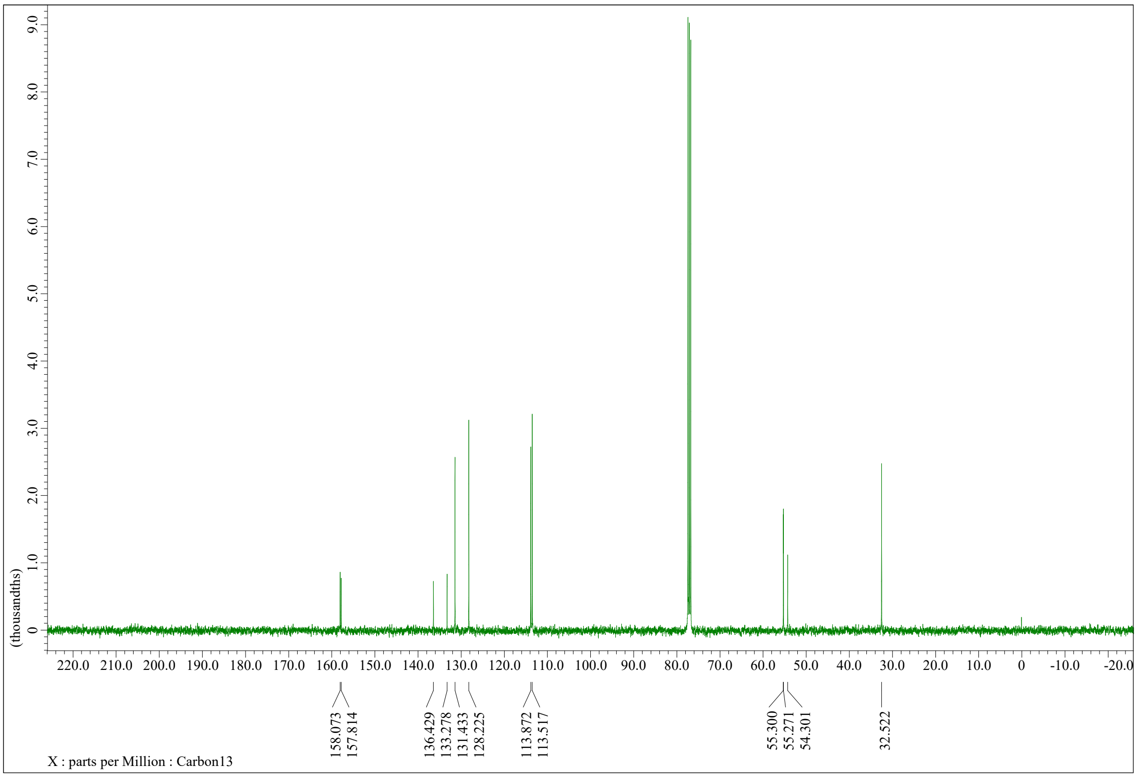

2d: ^{1}H NMR (400 MHz, $CDCl_3$)

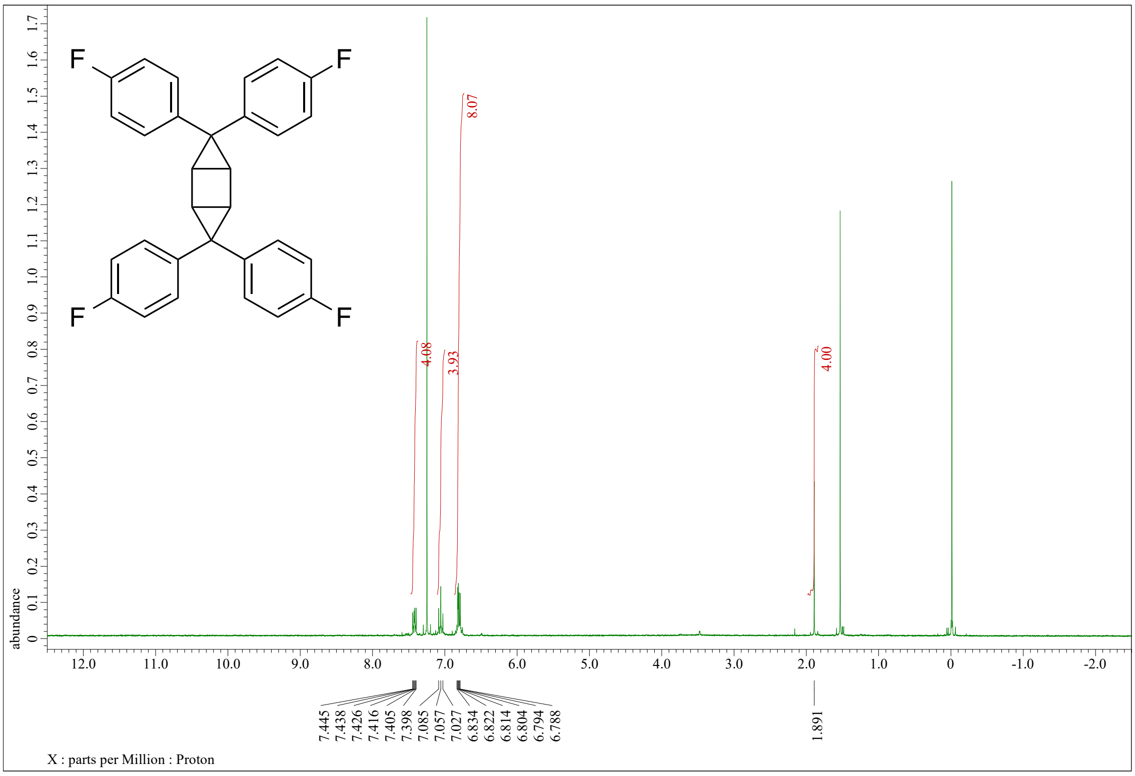

2d: $^{13}C\{^{1}H\}$ NMR (100 MHz, $CDCl_3$)

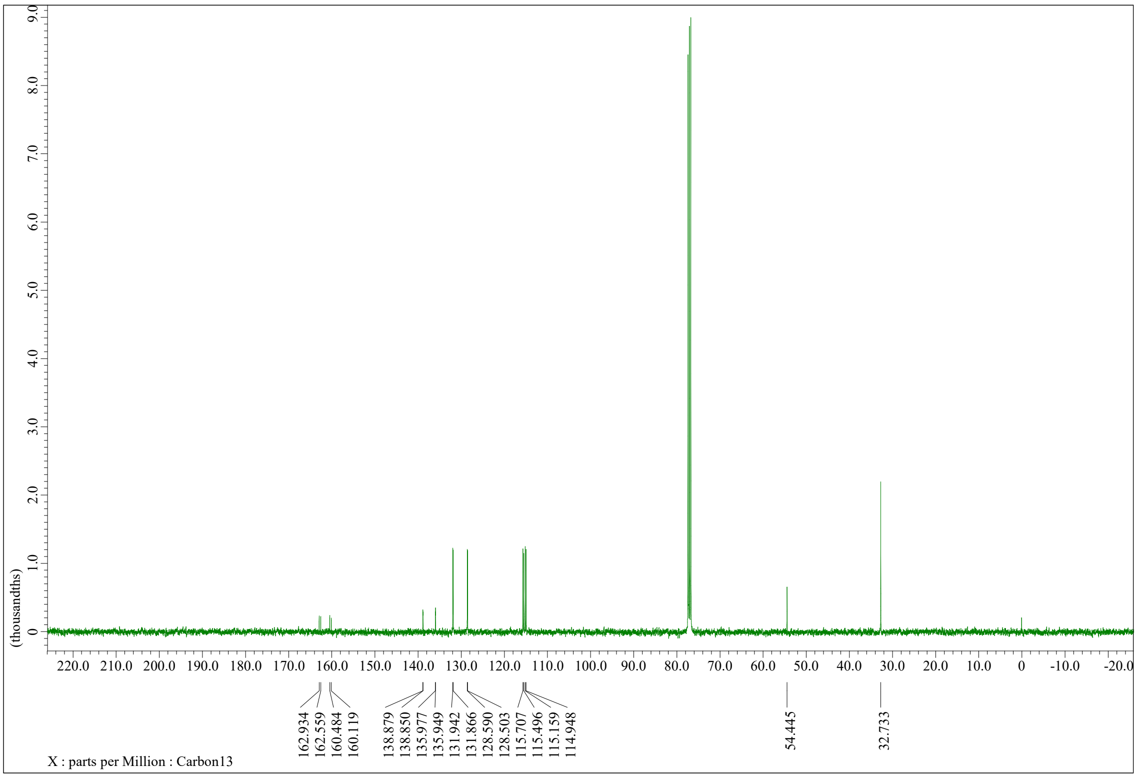

2d: ^{19}F NMR (375 MHz, $CDCl_3$)

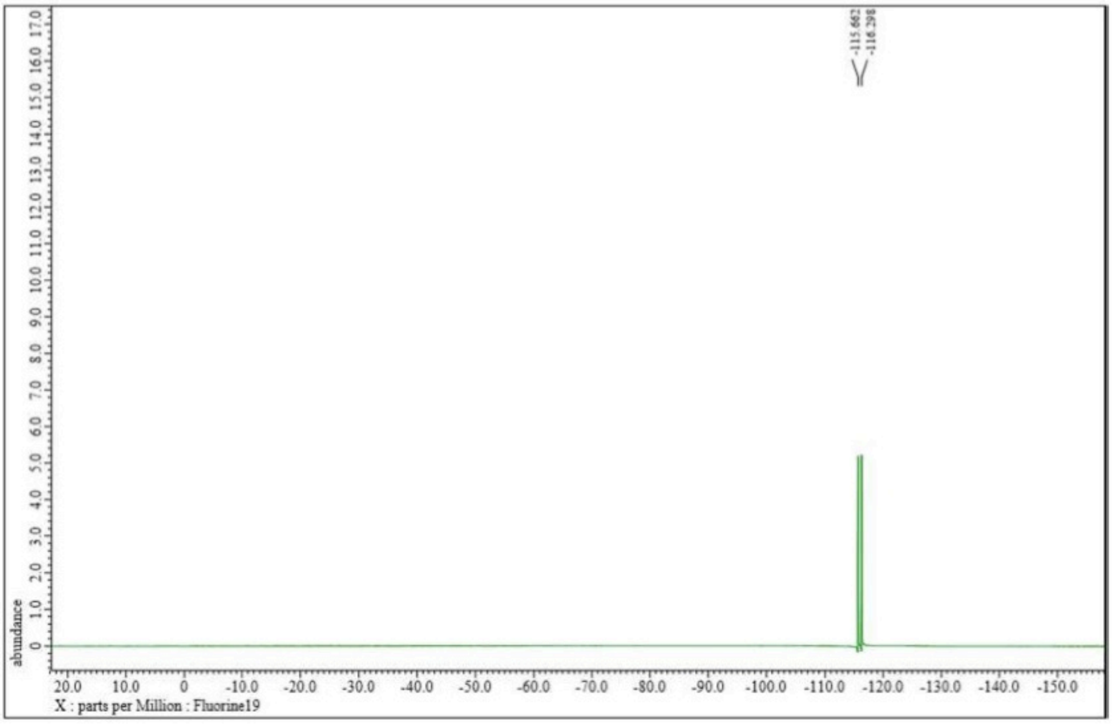

2e: ^{1}H NMR (400 MHz, $CDCl_3$)

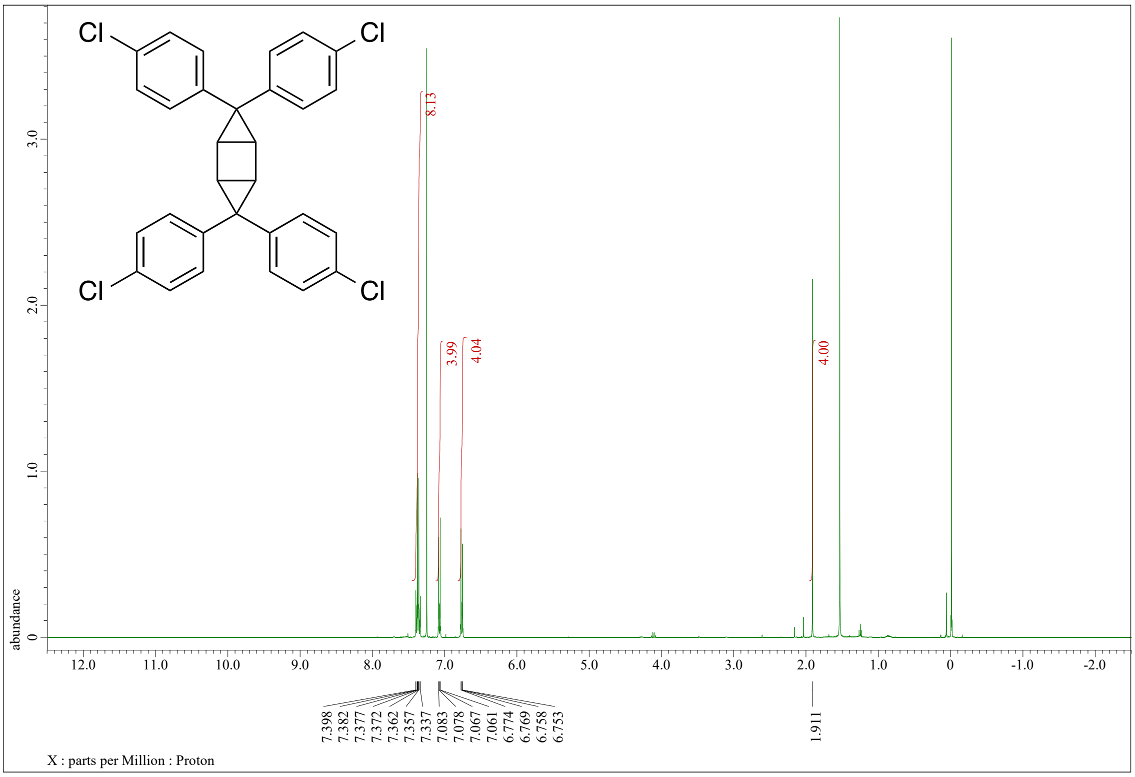

2e: $^{13}C\{^{1}H\}$ NMR (100 MHz, $CDCl_3$)

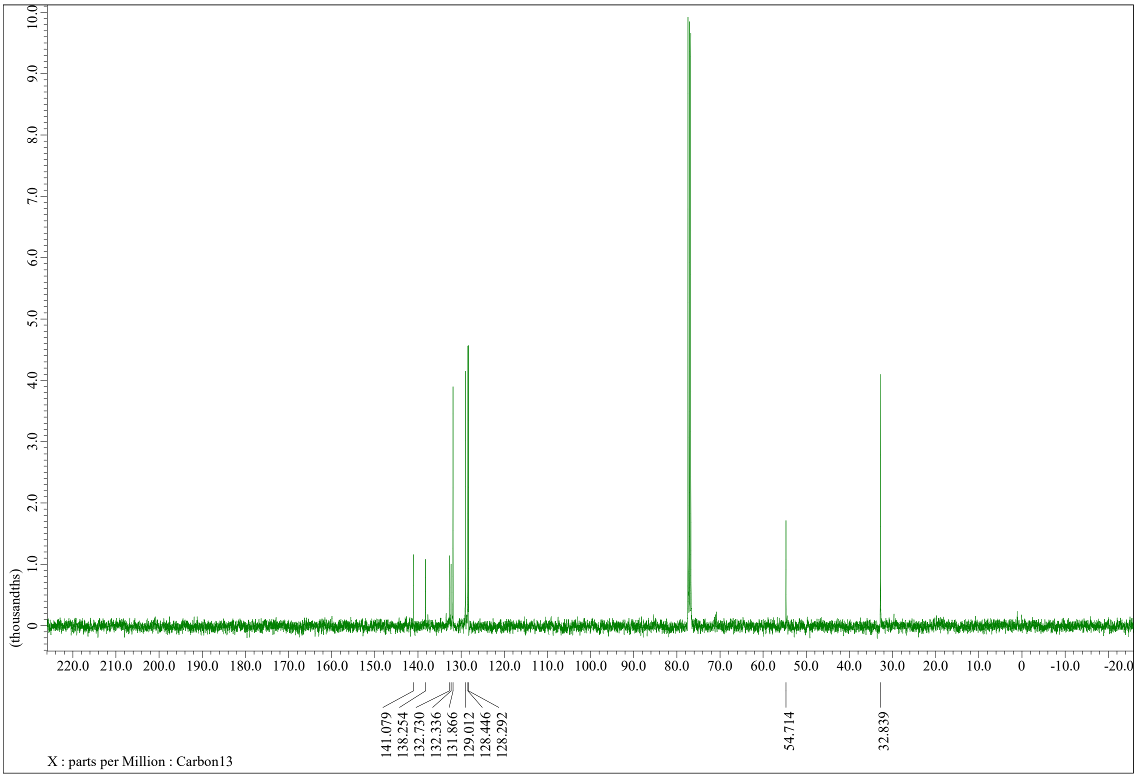

2f: ^{1}H NMR (400 MHz, $CDCl_3$)

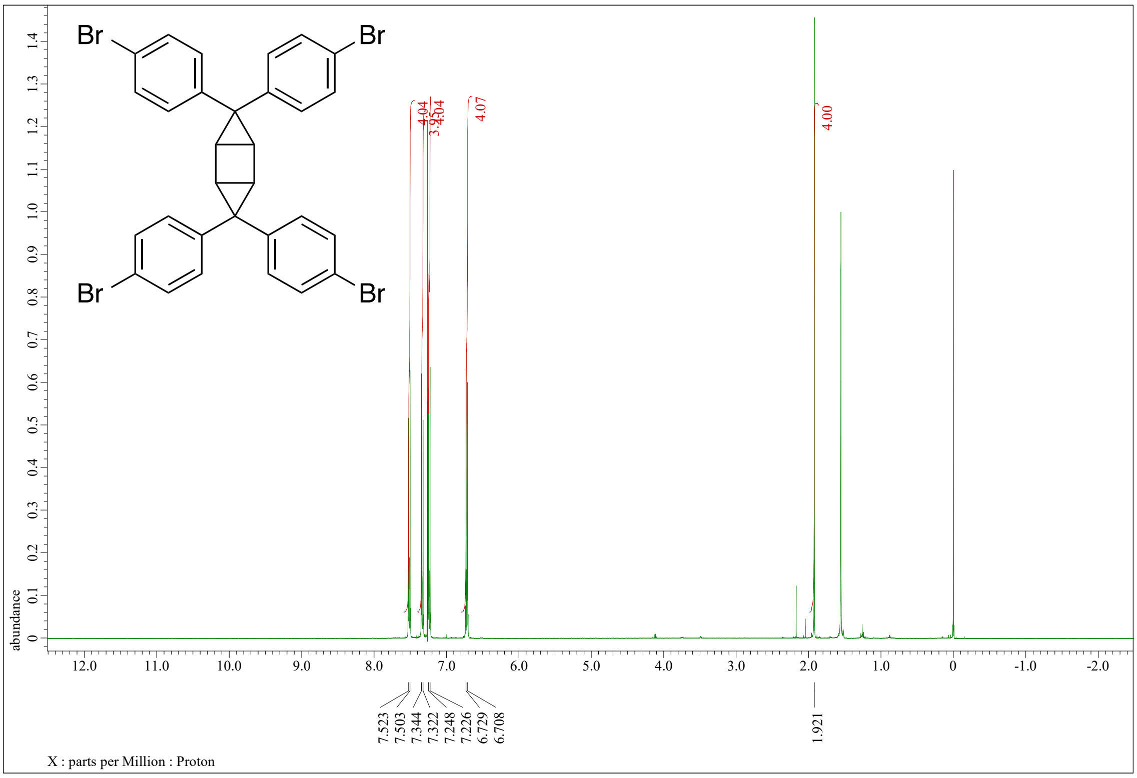

2f: ^{13}C{^{1}H} NMR (100 MHz, $CDCl_3$)

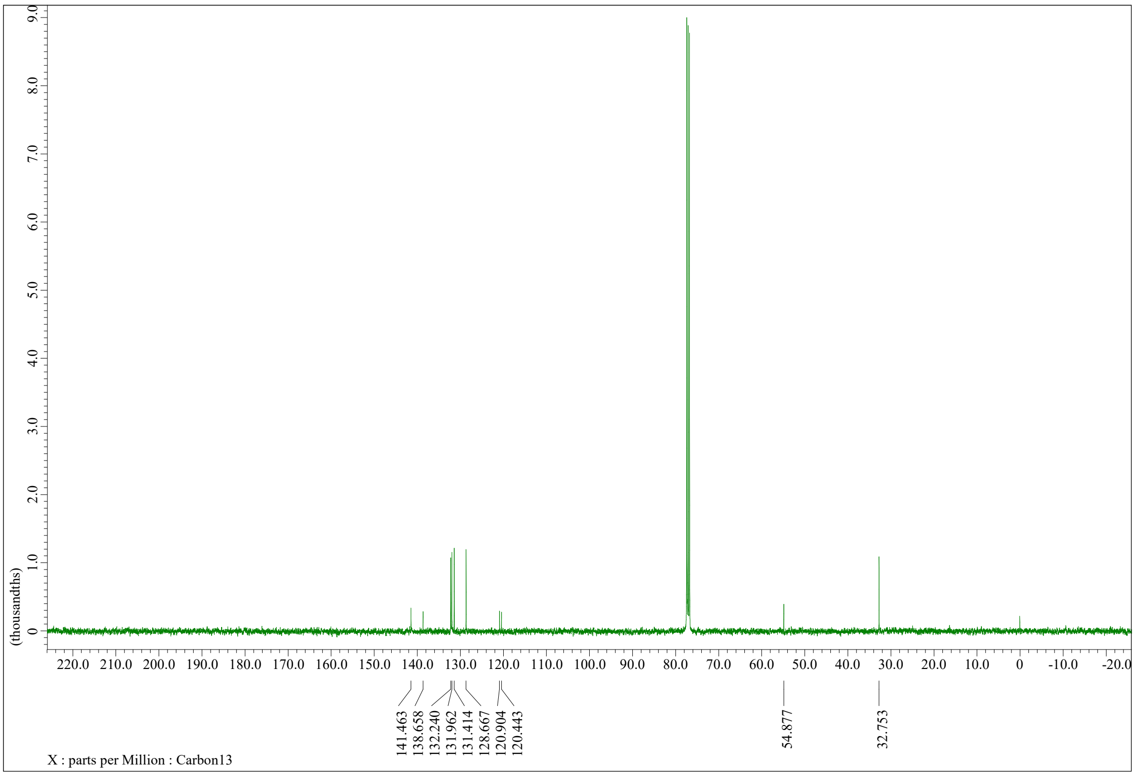

2g: ^{1}H NMR (400 MHz, $CDCl_3$)

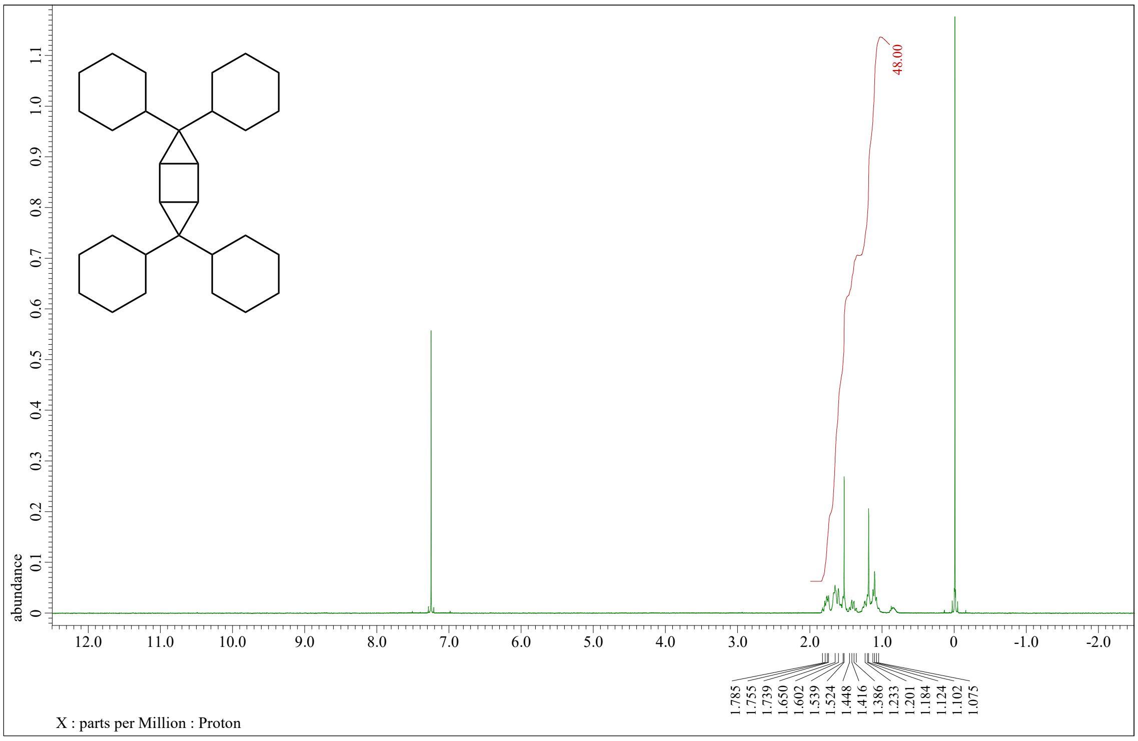

2g: $^{13}C\{^{1}H\}$ NMR (100 MHz, $CDCl_3$)

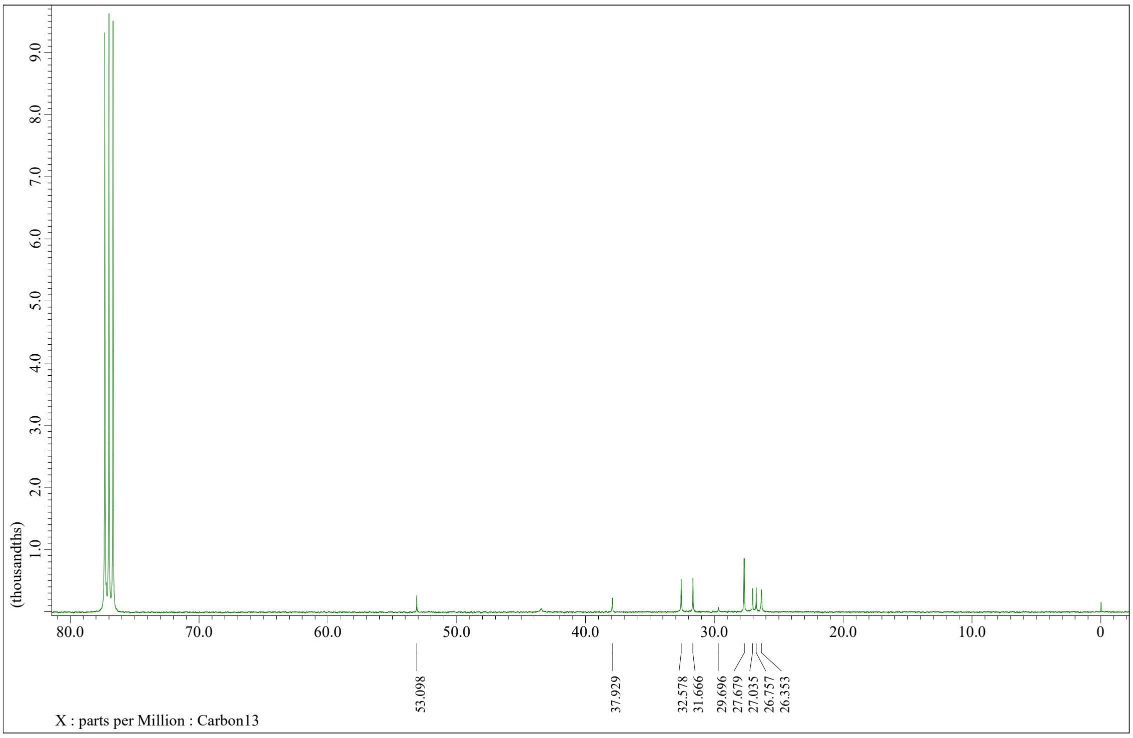

2h: ^{1}H NMR (400 MHz, $CDCl_3$)

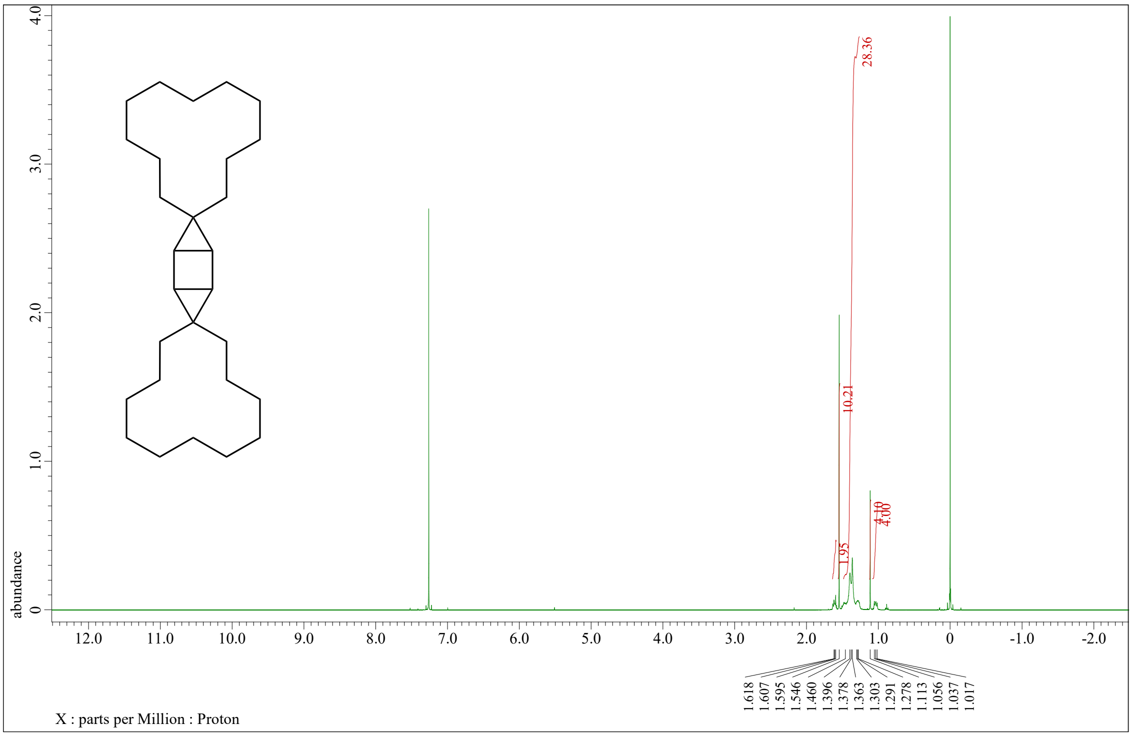

2h: ^{13}C{^{1}H} NMR (100 MHz, $CDCl_3$)

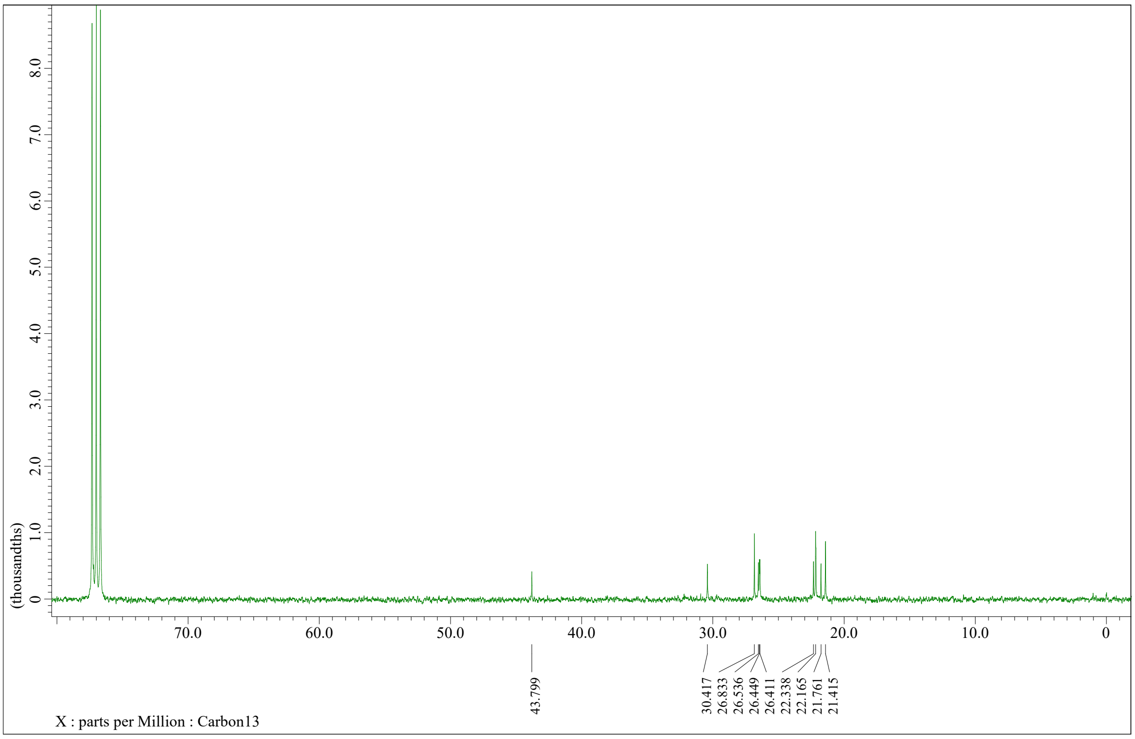

2i: ^{1}H NMR (400 MHz, CDCl$_3$)

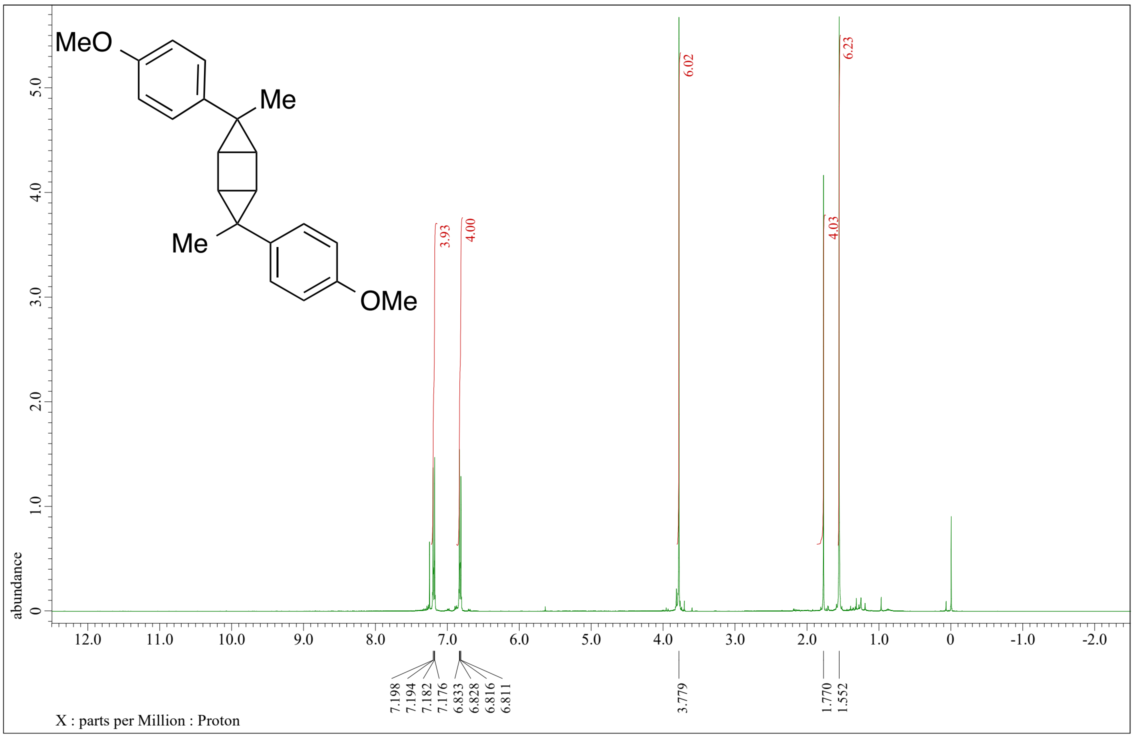

2i: ^{13}C{^{1}H} NMR (100 MHz, CDCl$_3$)

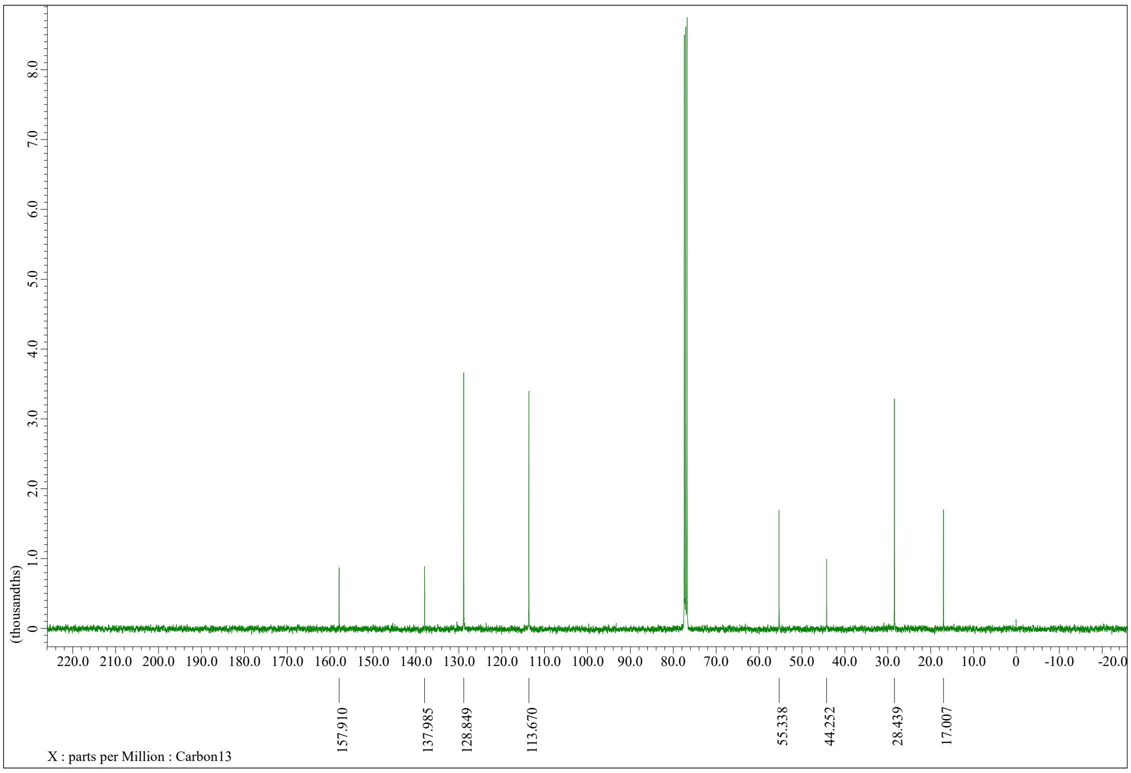

2j: ^{1}H NMR (400 MHz, $CDCl_3$)

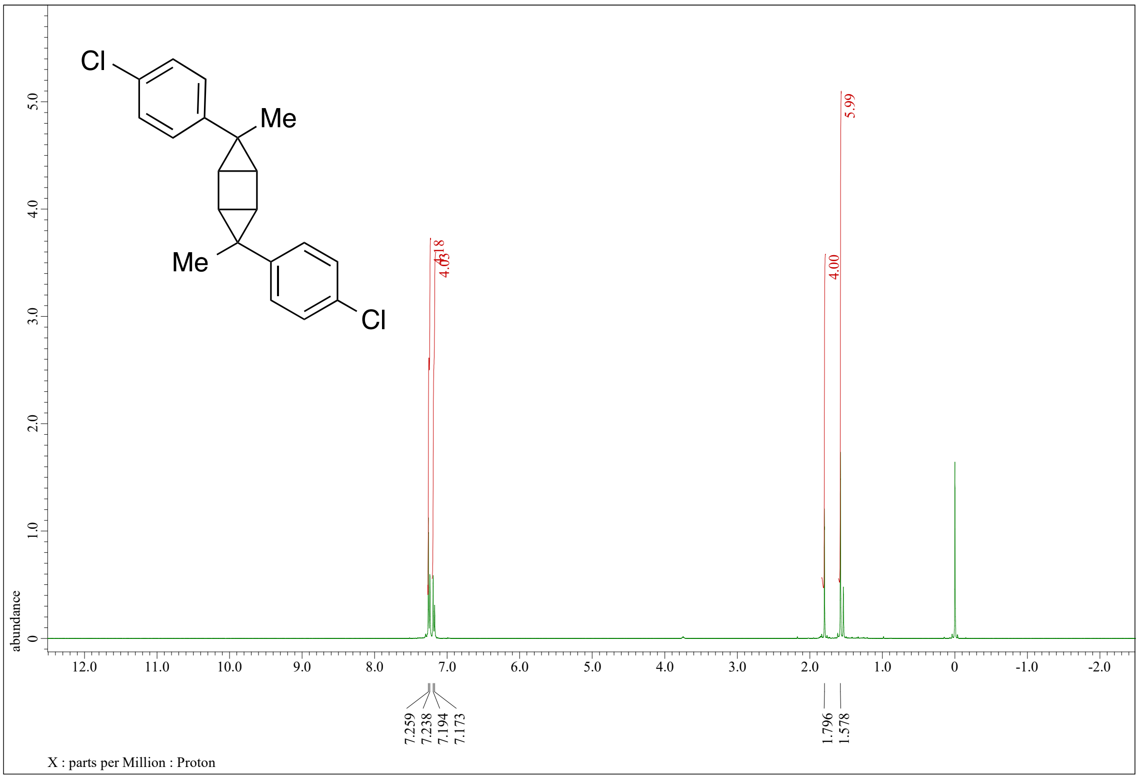

2j: ^{13}C{^{1}H} NMR (100 MHz, $CDCl_3$)

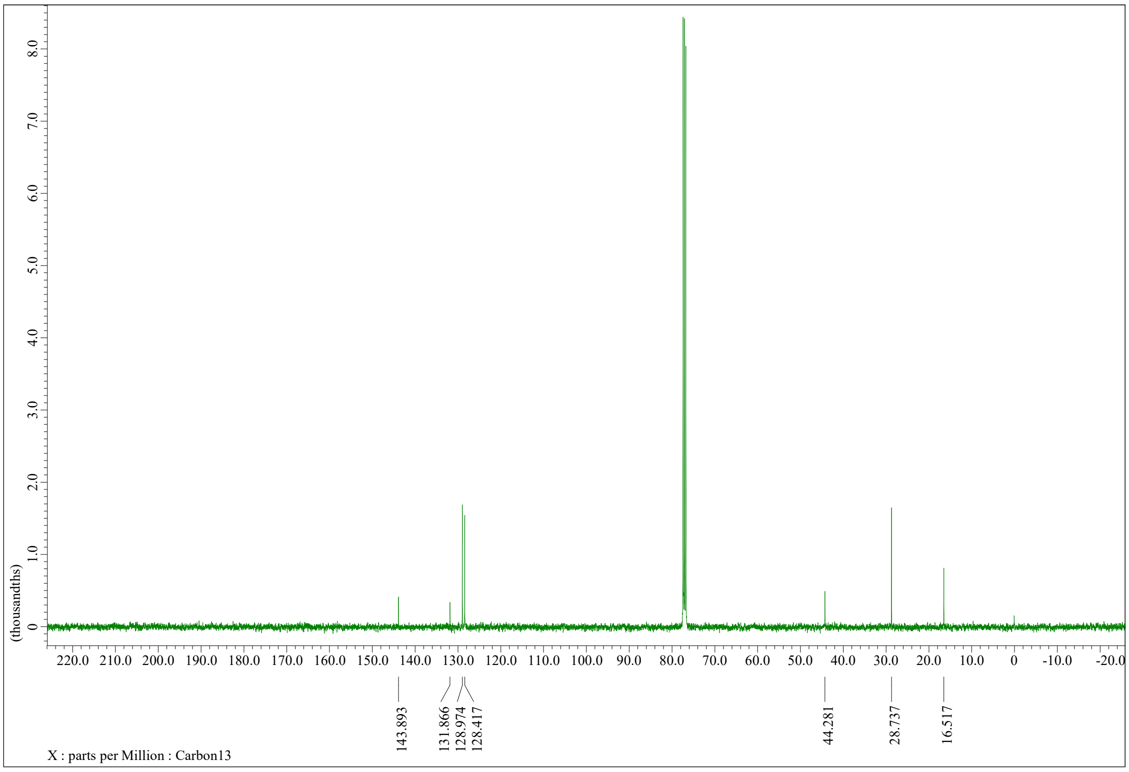

2k: ¹H NMR (400 MHz, $CDCl_3$)

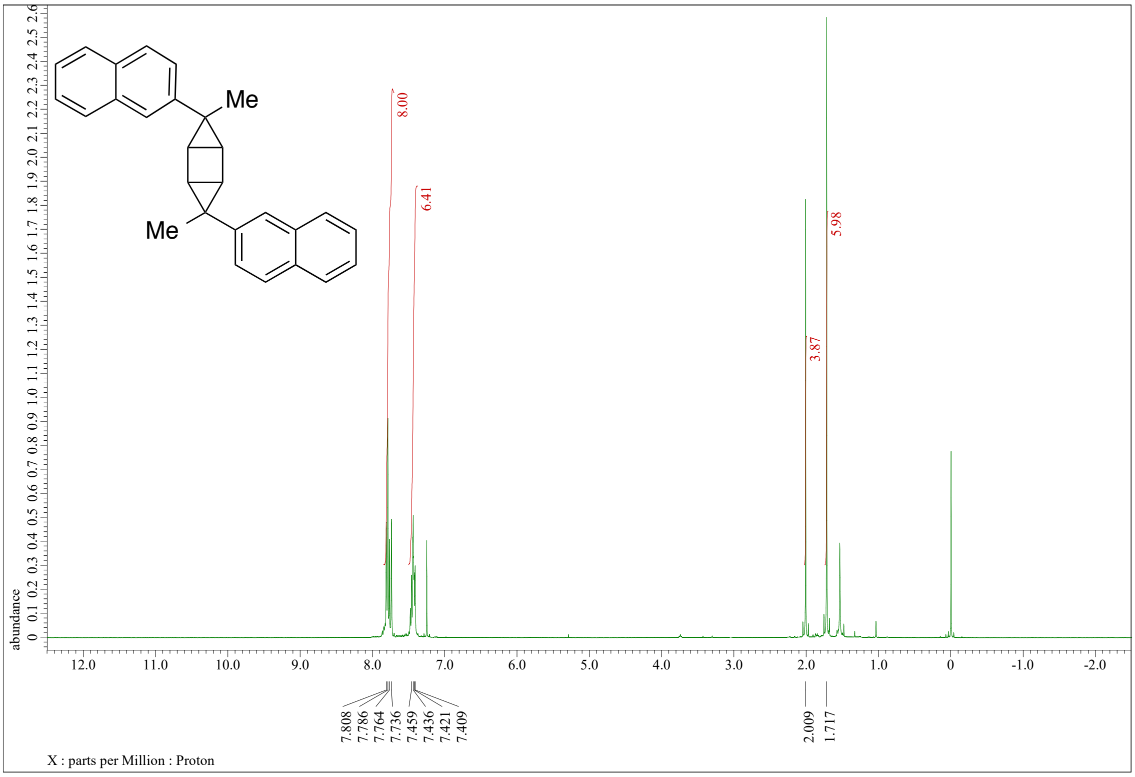

2k: $^{13}C\{^1H\}$ NMR (100 MHz, $CDCl_3$)

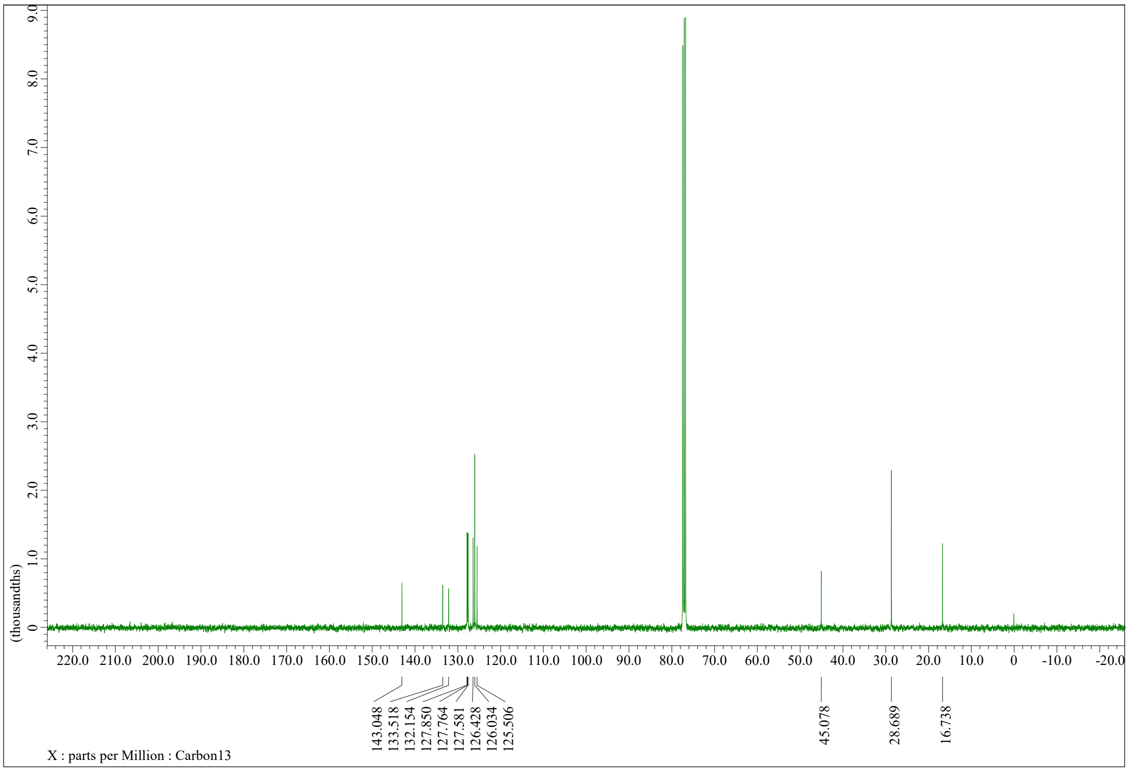

Predicted stereochemistry of isolated 2i–k and 3i–k

Figure S1. Proposed stereochemistry of compound **2**

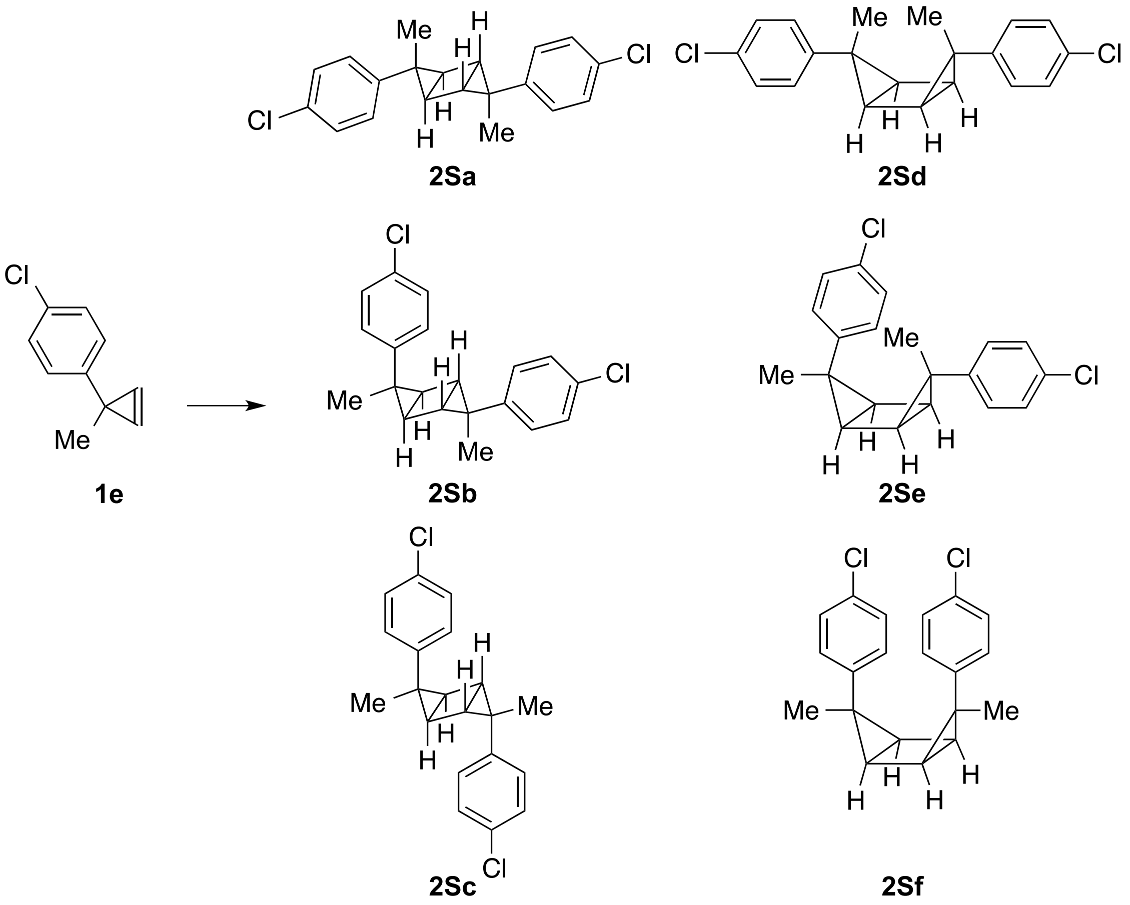

The reaction of cyclopropenes **1e** would give chair-like conformation **2Sa**–**c** and boat-like conformation **2Sd**–**f**. The steric repulsion in boat-like conformation would inhibit the generation of **2Sd**–**f**. Accordingly, the comparison between experimental NMR spectra and predicted NMR spectra are described as followings.

Figure S2. ^{1}H NMR spectra of **2**

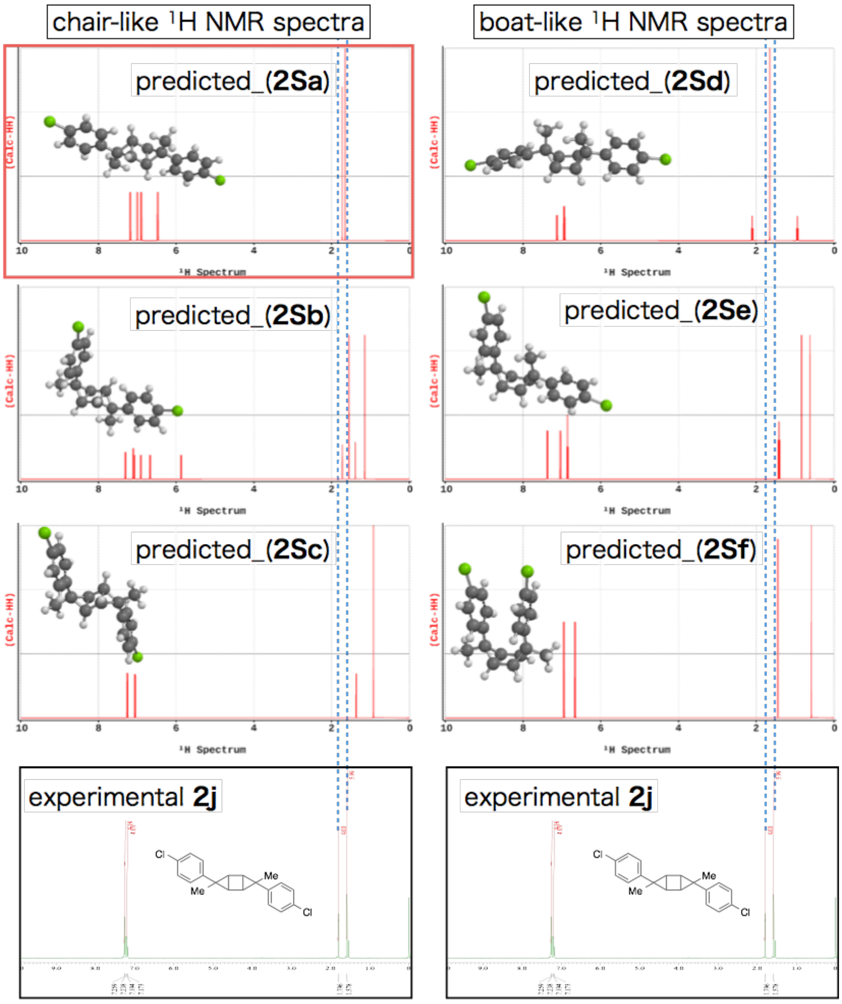

The predicted ^{1}H NMR spectra of **2Sa–f** and experimental spectra of **2j** are shown in Figure S2 (B3LYP/6-31G*). Typically, the relative energy of boat-like **2Sd**, **2Se**, and **2Sf** are ca. 30 kcal/mol higher than that of **2Sa**, **2Sb**, and **2Sc**; thus, **2Sa**, **2Sb**, and **2Sc** may include the appropriate stereochemistry of products. The predicted spectrum of optimized geometries **2Sa–f** indicates that the experimental ^{1}H NMR spectrum would be similar to that of **2Sa**. The peaks attributed to C–H of cyclobutane ring and CH_3 groups seem to be around 1.5–2.0 ppm due to the anisotropic effect from aryl groups on both C–H of cyclobutane ring and CH_3 groups.

Figure S3. ^{13}C NMR spectra of **3**

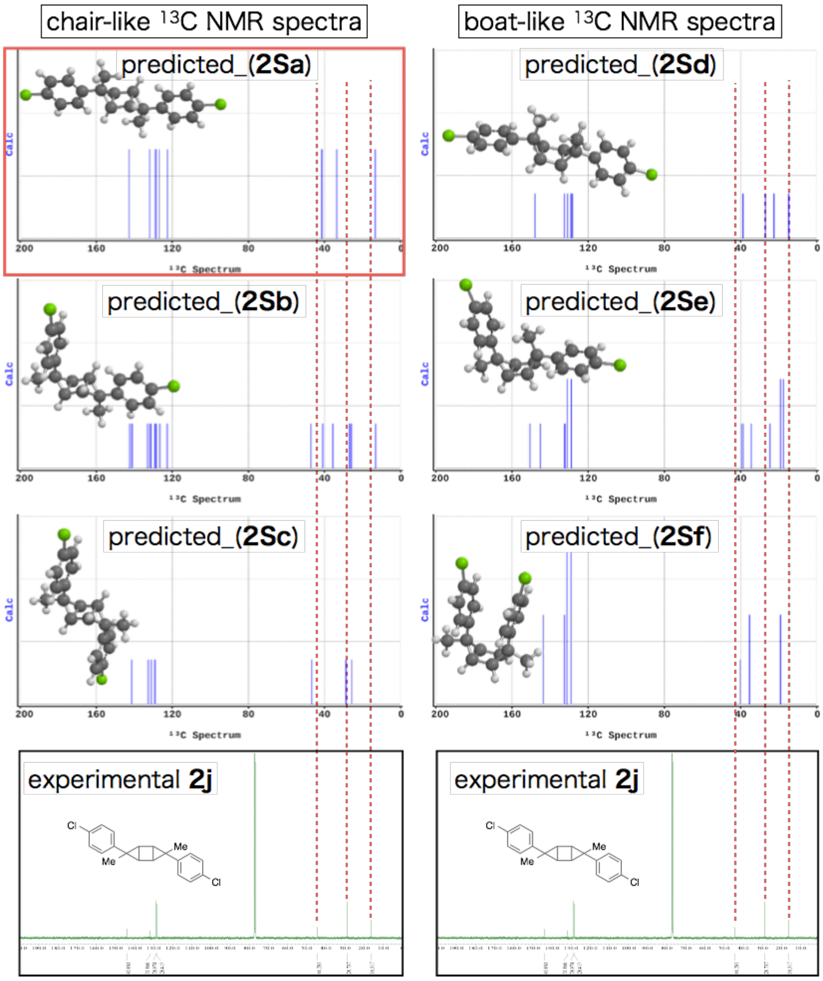

The experimental ^{13}C NMR spectrum is also similar to that of predicted_(**2Sa**) (Figure S3). Therefore, experimental ^{1}H NMR and ^{13}C NMR analyses and prediction indicates that the stereochemistry of **2i**–**k** seems to be *trans* and chair-like conformation. Although the experimental and predicted spectra are clear evidences for the isolation of *trans*-isomers, the generation of *cis*-isomers could be considered. The *cis*-isomers might be removed via the purification by silica gel column chromatography.

In this context, the thermal ring opening-reaction of *trans*-**2i**–**k** would give *trans*-**3i**–**k**. Since the stereo centers in compound **2** do not participate in the C–C bond dissociation and formation, there is no room for the isomerization of stereochemistry. Typically, *trans*-1,4-cyclohexadienes are solid, but *cis*-1,4-cyclohexadienes via the Birch reduction of *p*-terphenyl are oil at room temperature.[S1]

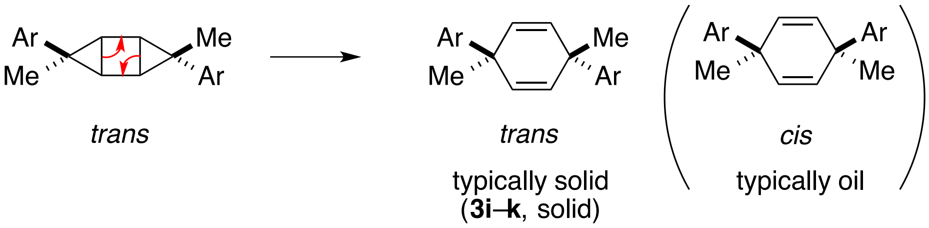

3a: ^{1}H NMR (400 MHz, CDCl$_3$)

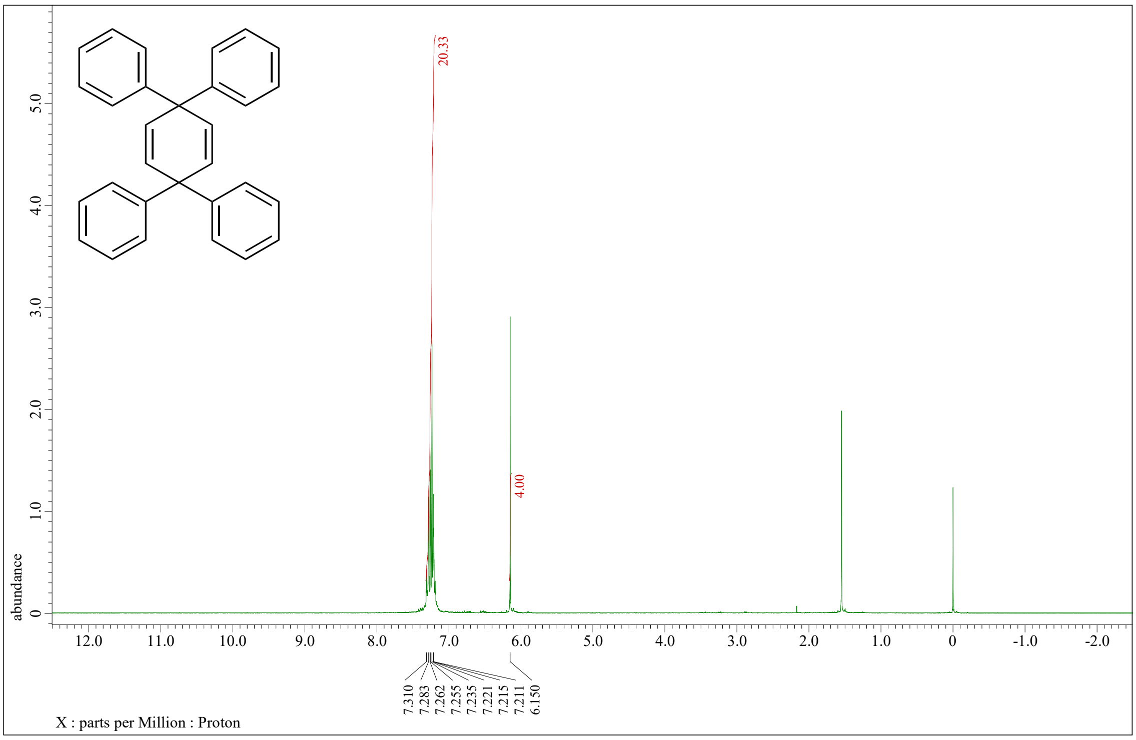

3a: ^{13}C{^{1}H} NMR (100 MHz, CDCl$_3$)

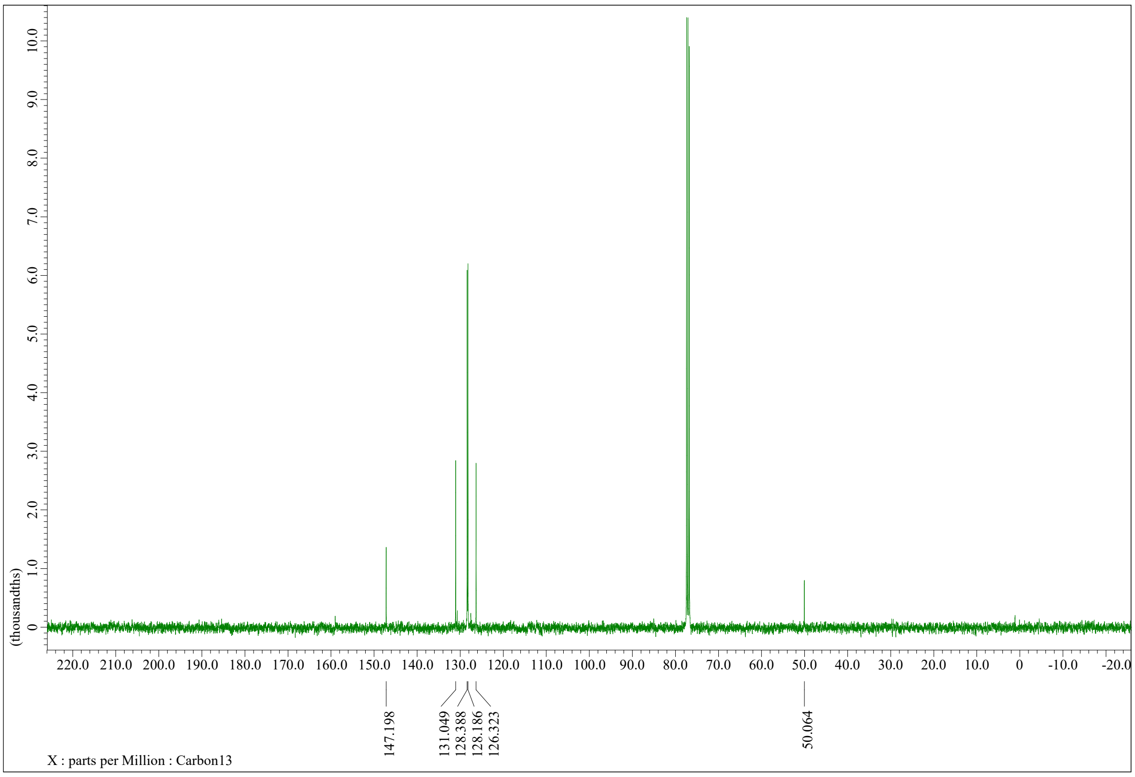

3b: ^{1}H NMR (400 MHz, $CDCl_3$)

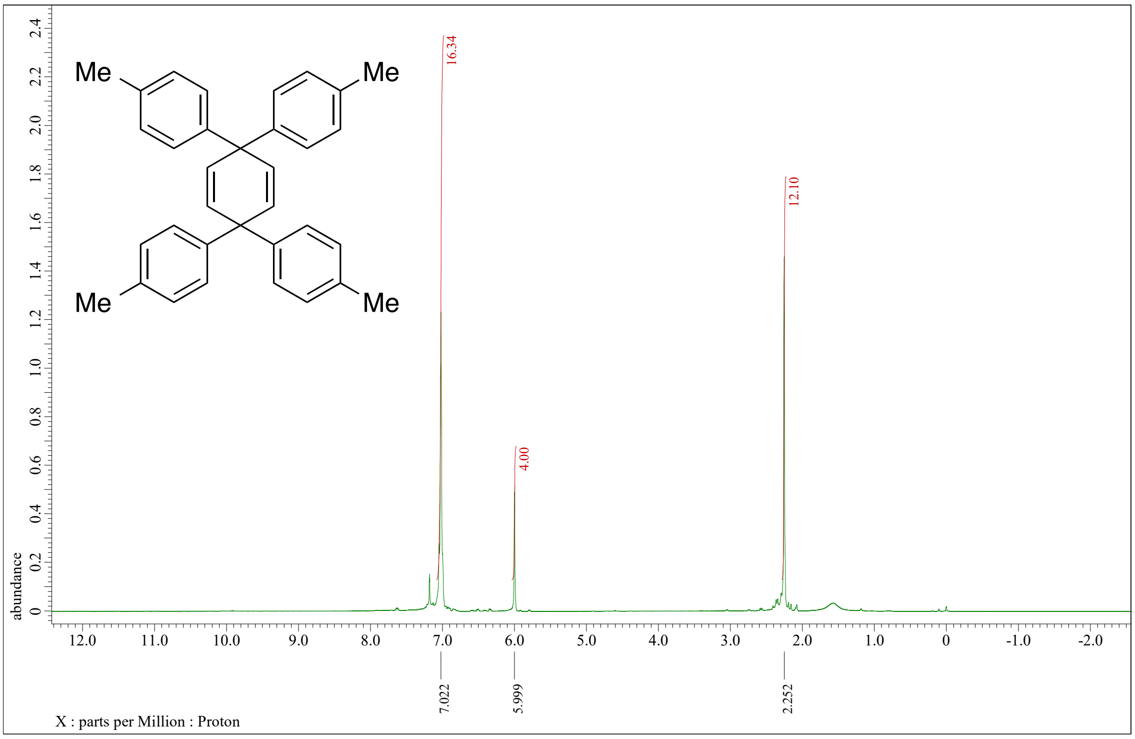

3b: $^{13}C\{^{1}H\}$ NMR (100 MHz, $CDCl_3$)

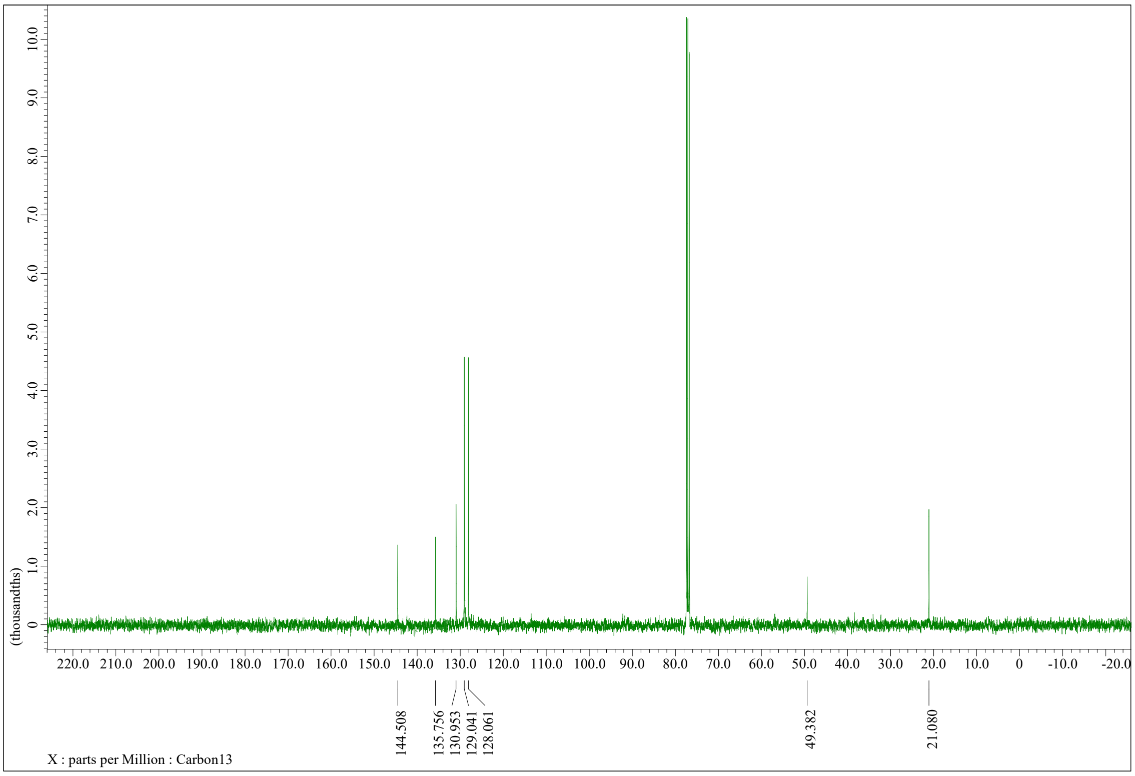

3c: ^{1}H NMR (400 MHz, $CDCl_3$)

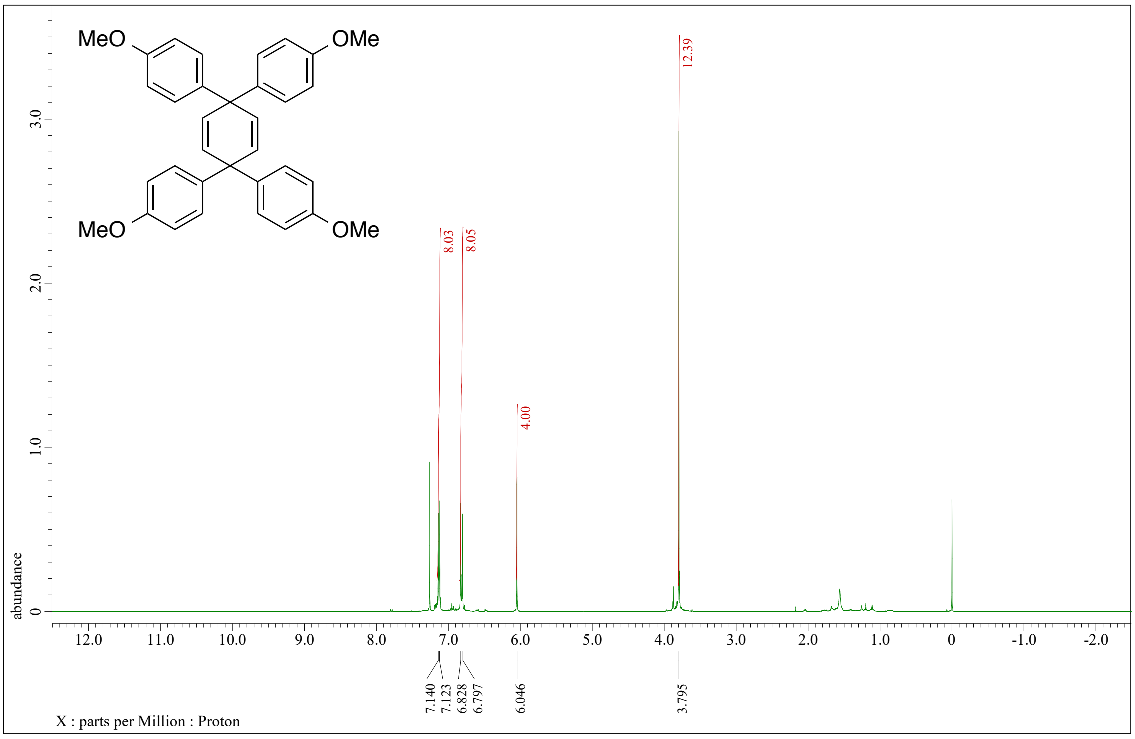

3c: $^{13}C\{^{1}H\}$ NMR (100 MHz, $CDCl_3$)

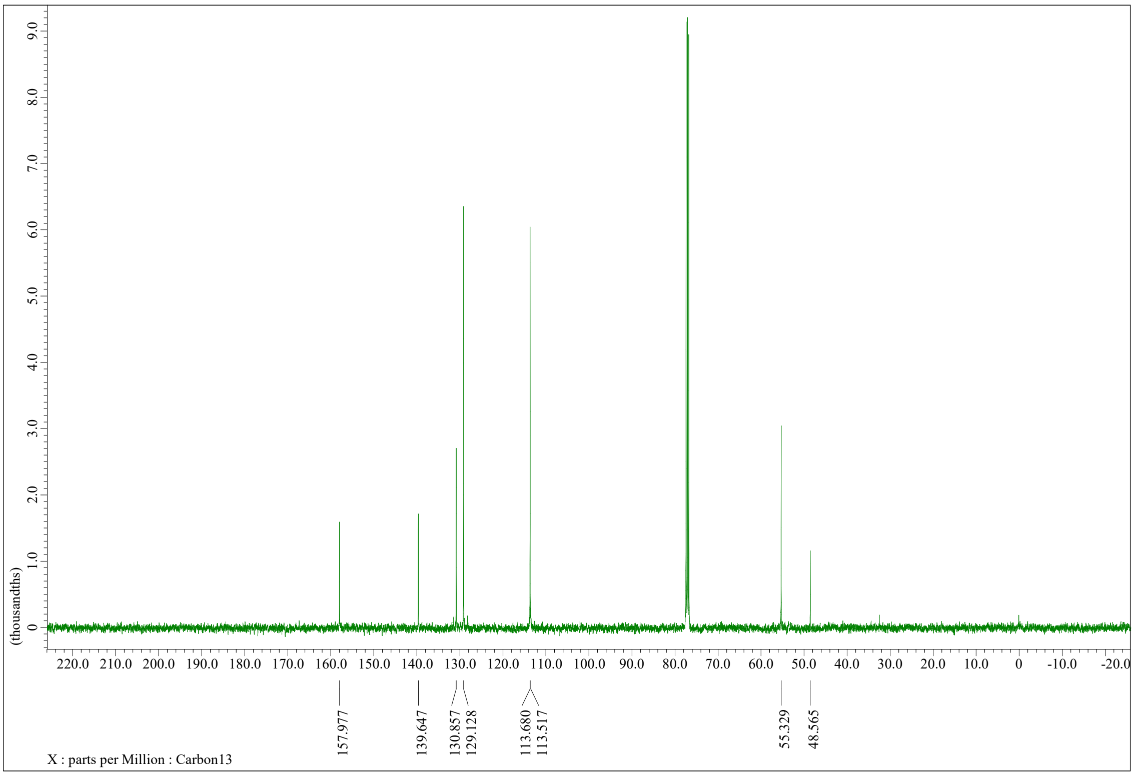

3d: ^{1}H NMR (400 MHz, $CDCl_3$)

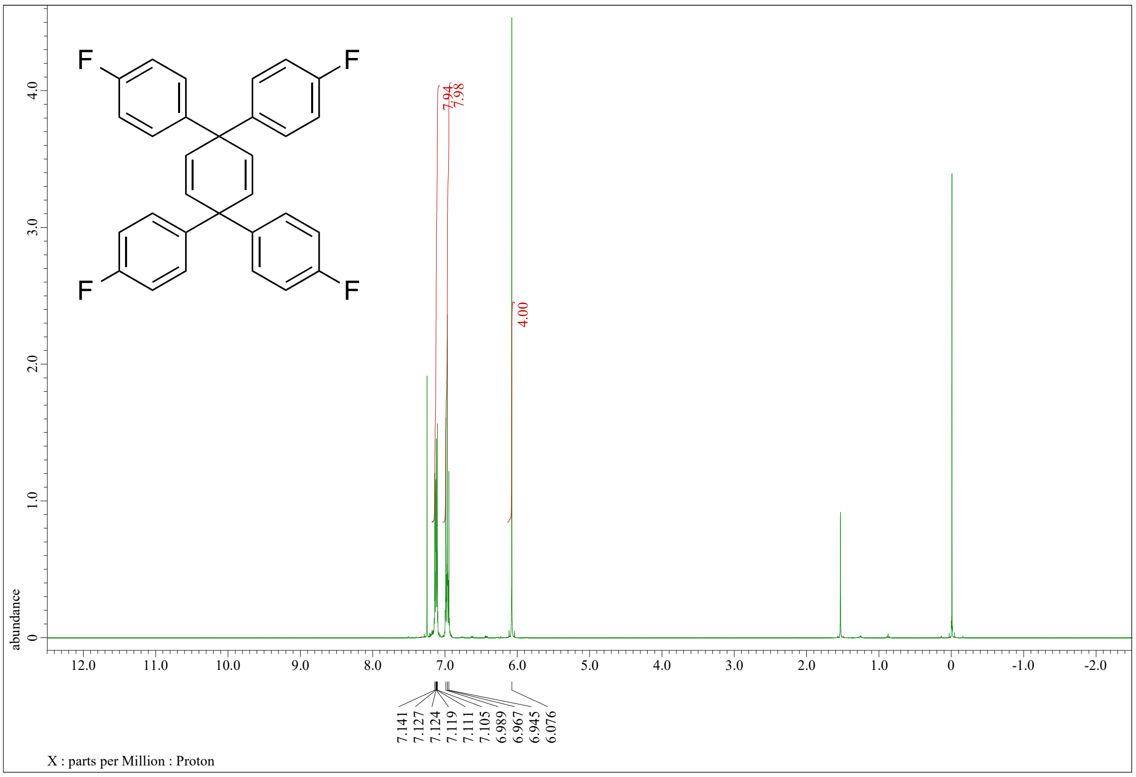

3d: $^{13}C\{^{1}H\}$ NMR (100 MHz, $CDCl_3$)

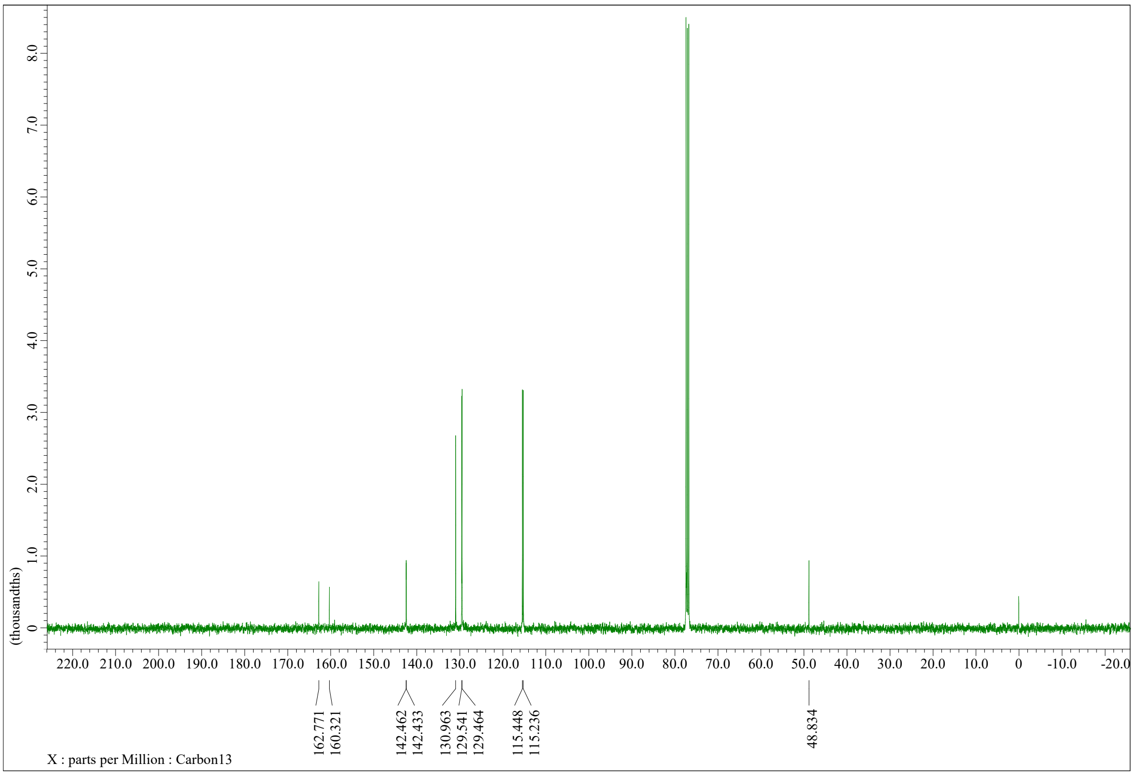

3d: ^{19}F NMR (375 MHz, $CDCl_3$)

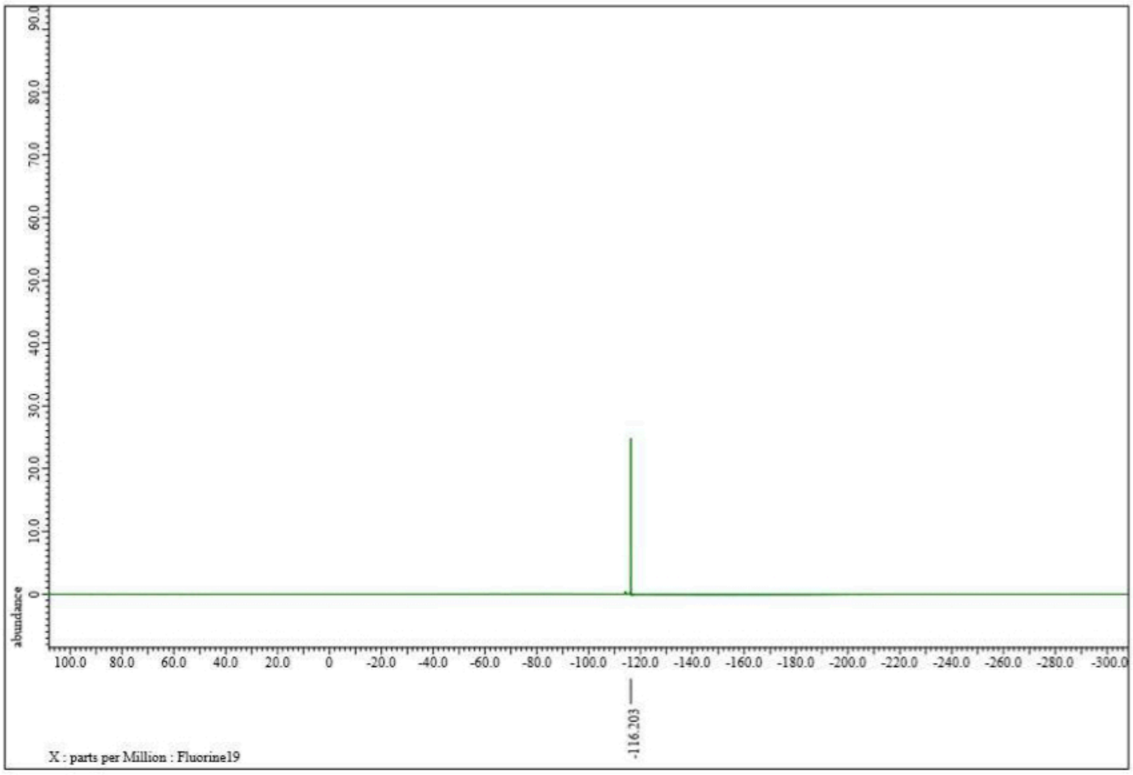

3e: ^{1}H NMR (400 MHz, $CDCl_3$)

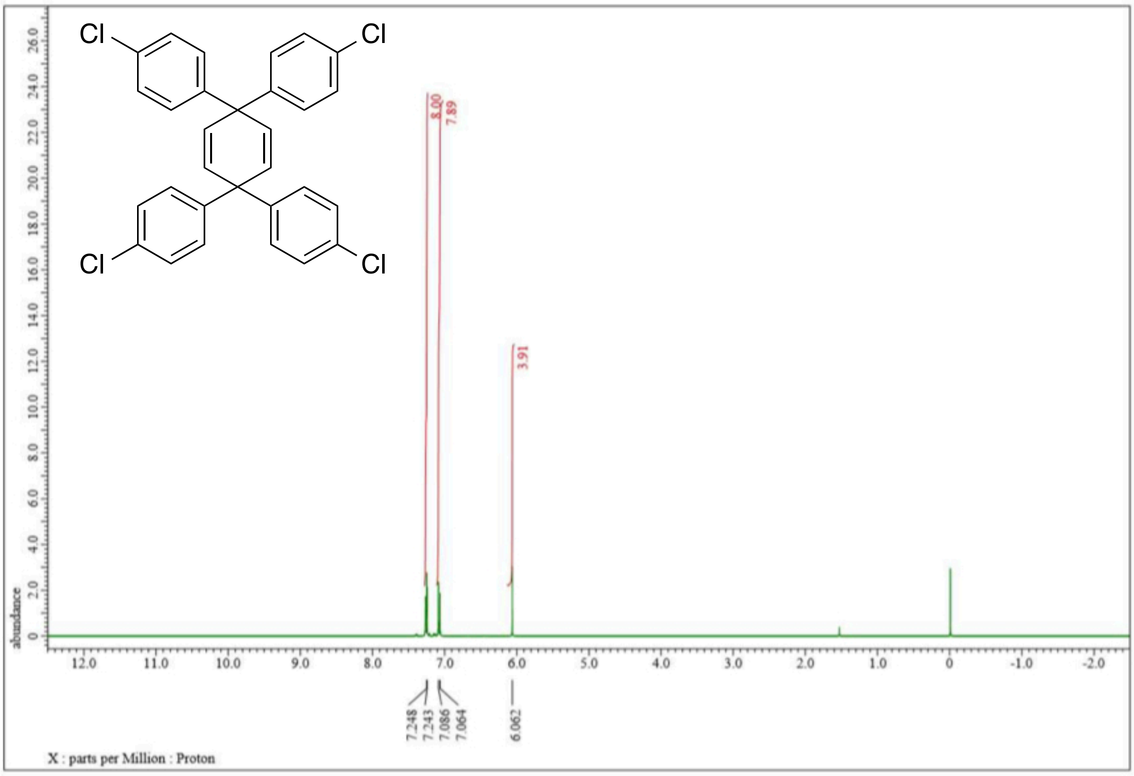

3e: ^{13}C{^{1}H} NMR (100 MHz, $CDCl_3$)

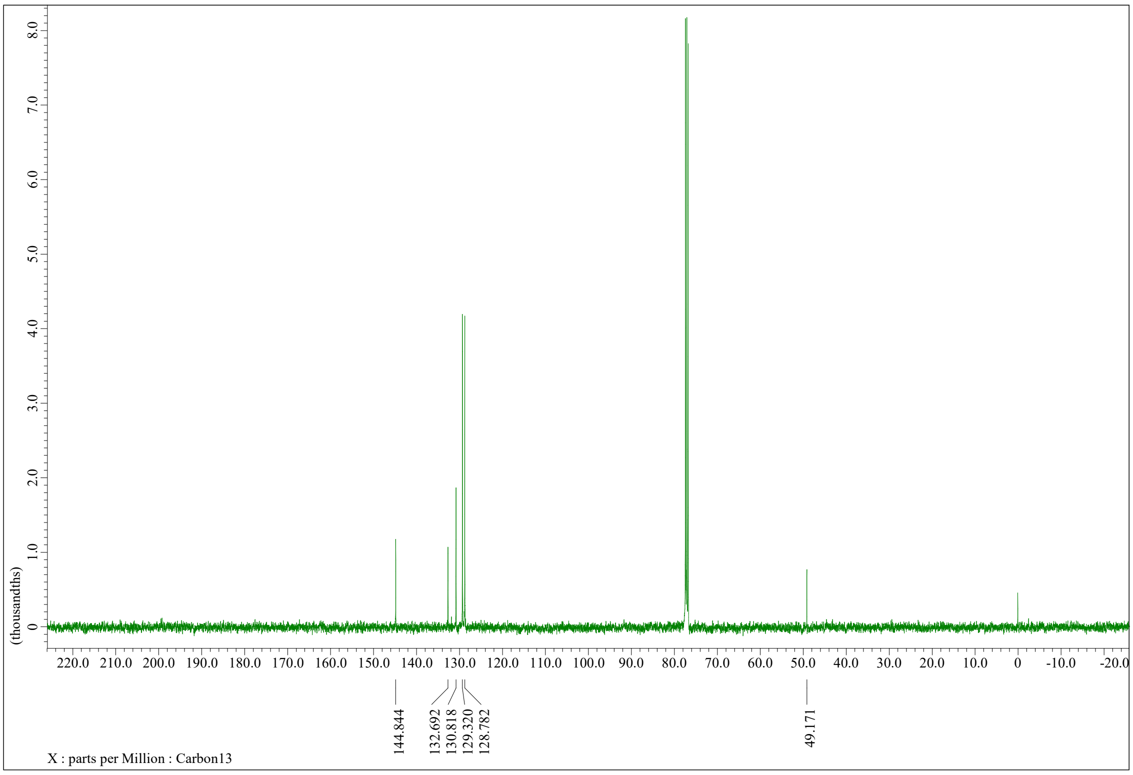

3f: ^{1}H NMR (400 MHz, $CDCl_3$)

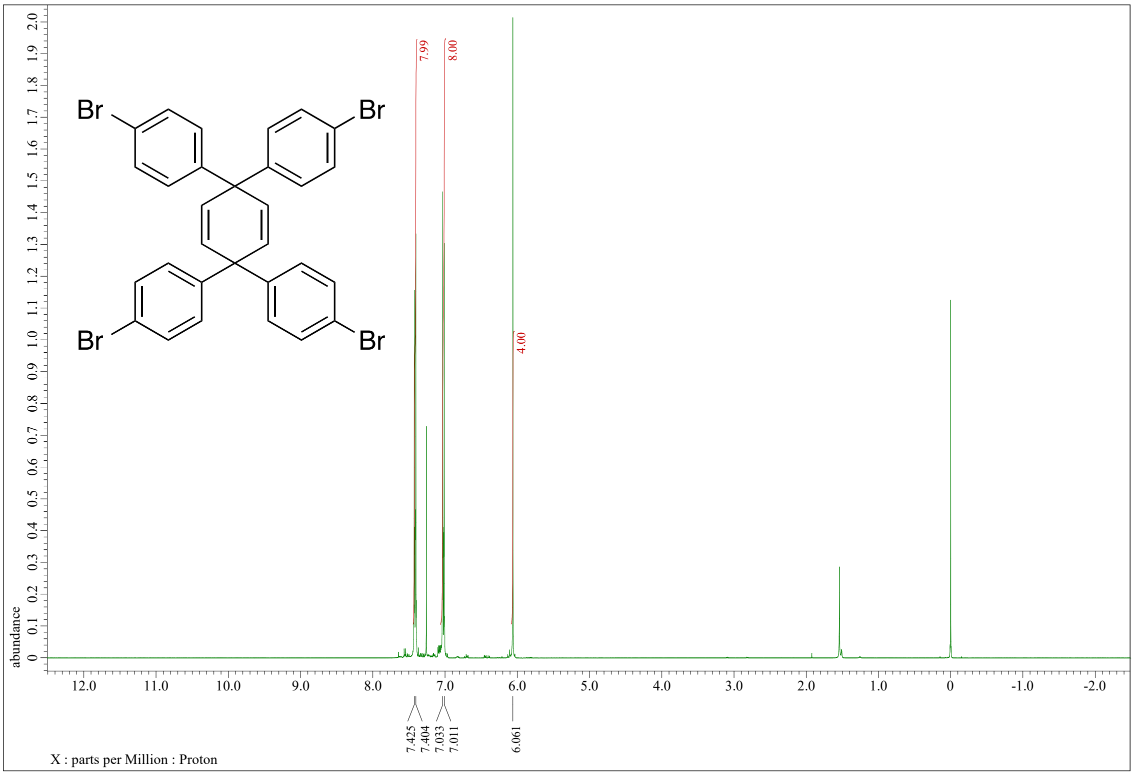

3f: ^{13}C{^{1}H} NMR (100 MHz, $CDCl_3$)

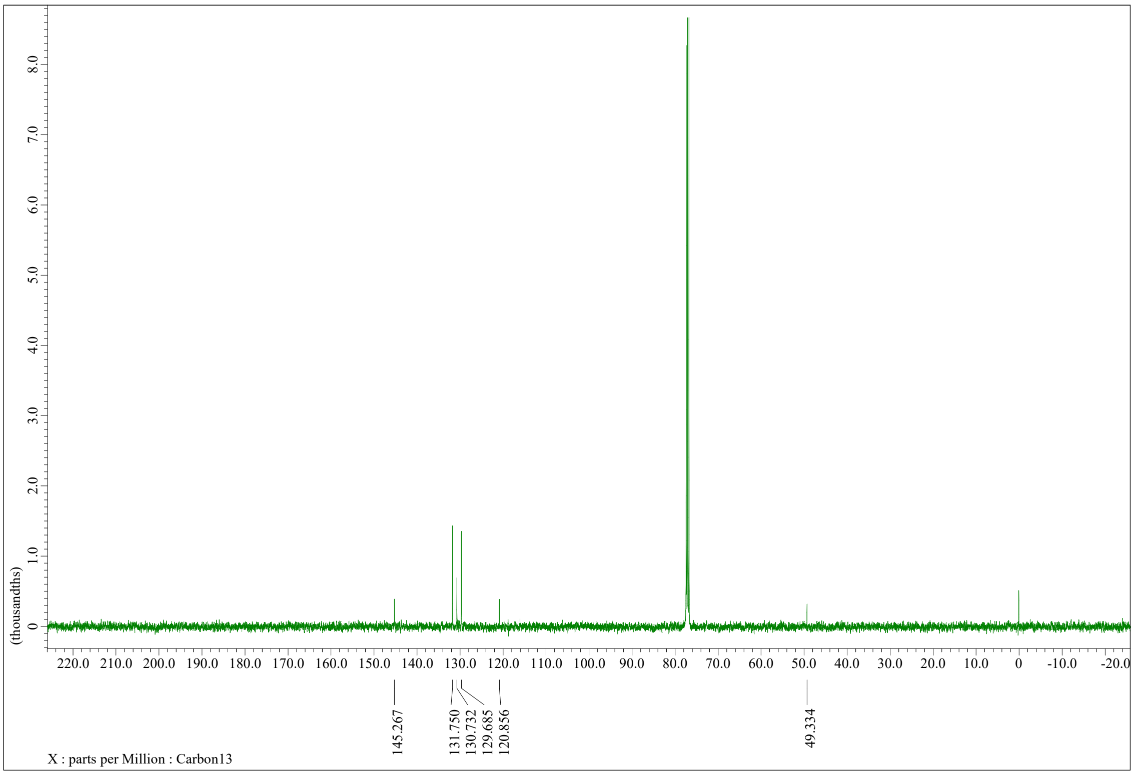

3g: ^{1}H NMR (400 MHz, $CDCl_3$)

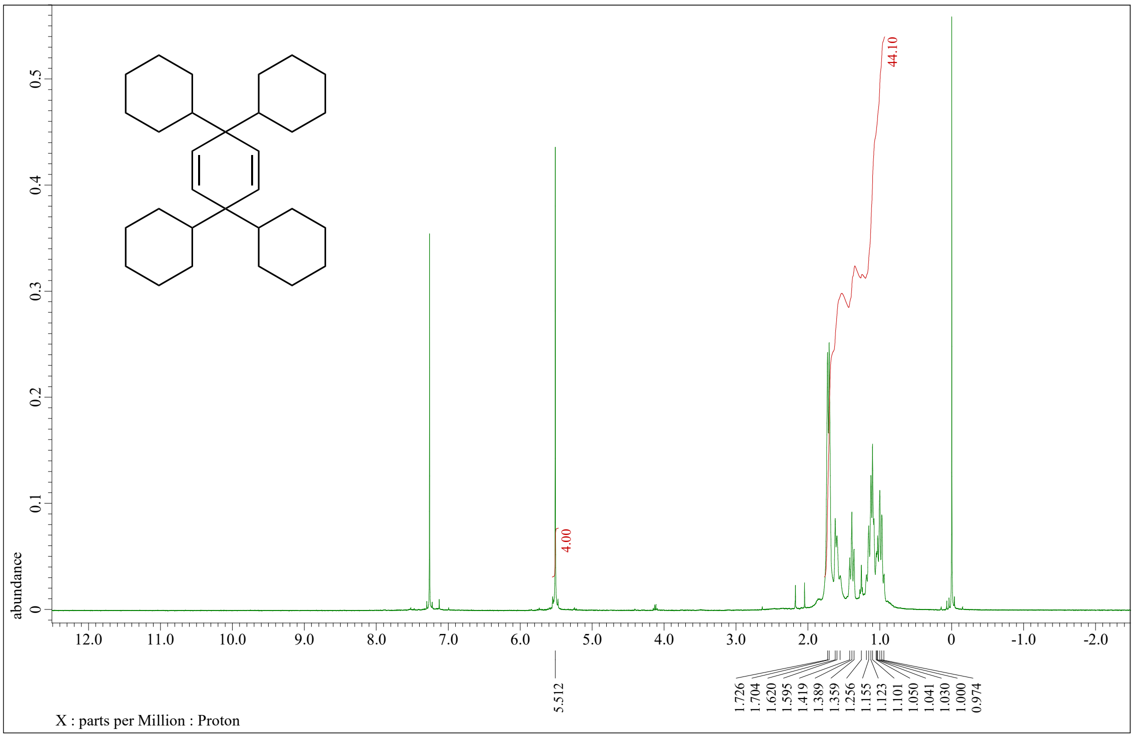

3g: ^{13}C{^{1}H} NMR (100 MHz, $CDCl_3$)

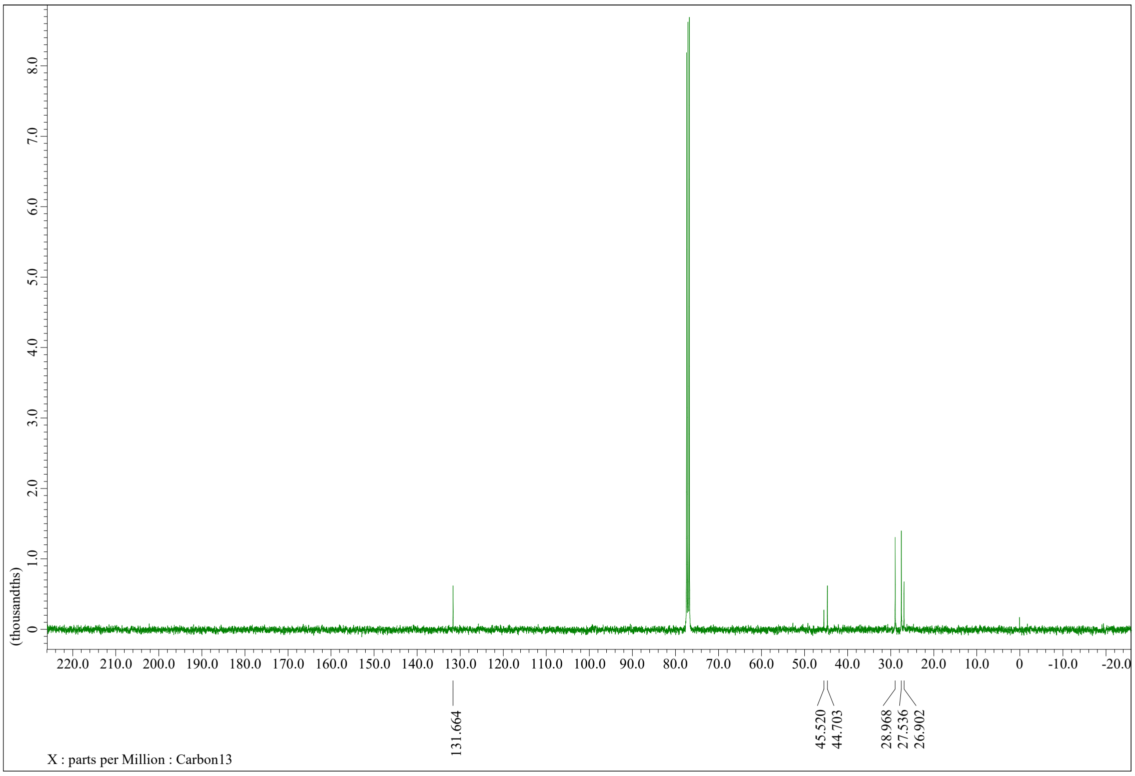

3h: ^{1}H NMR (400 MHz, $CDCl_3$)

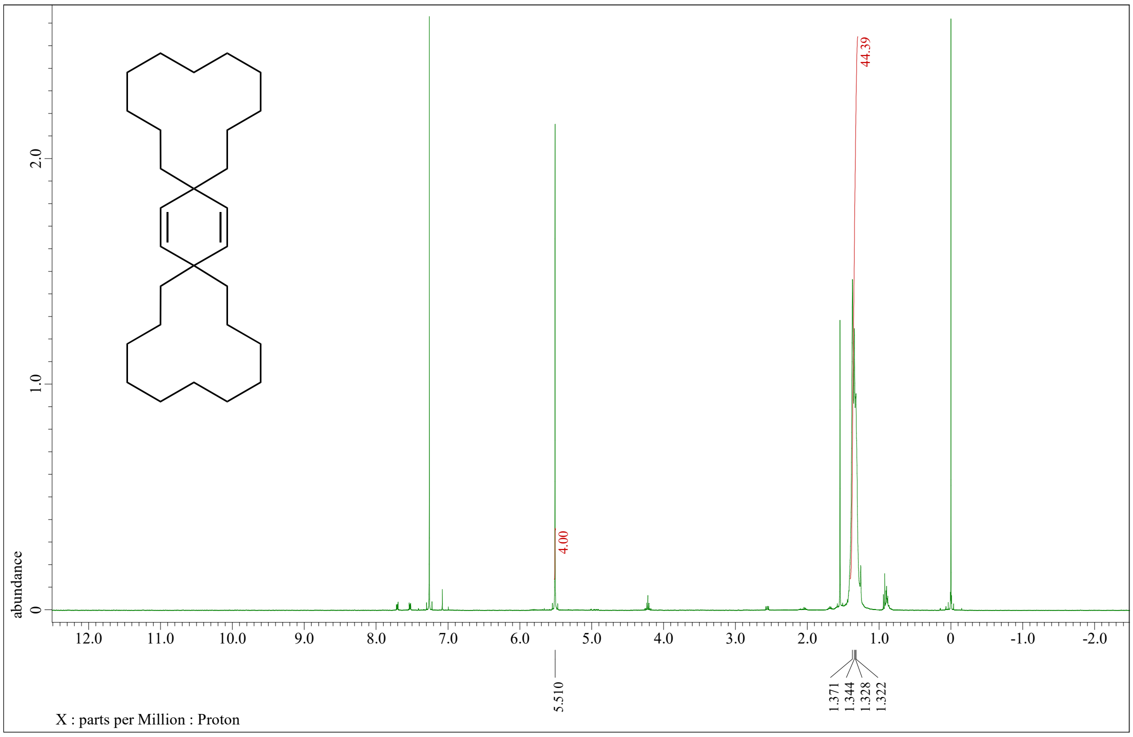

3h: $^{13}C\{^{1}H\}$ NMR (100 MHz, $CDCl_3$)

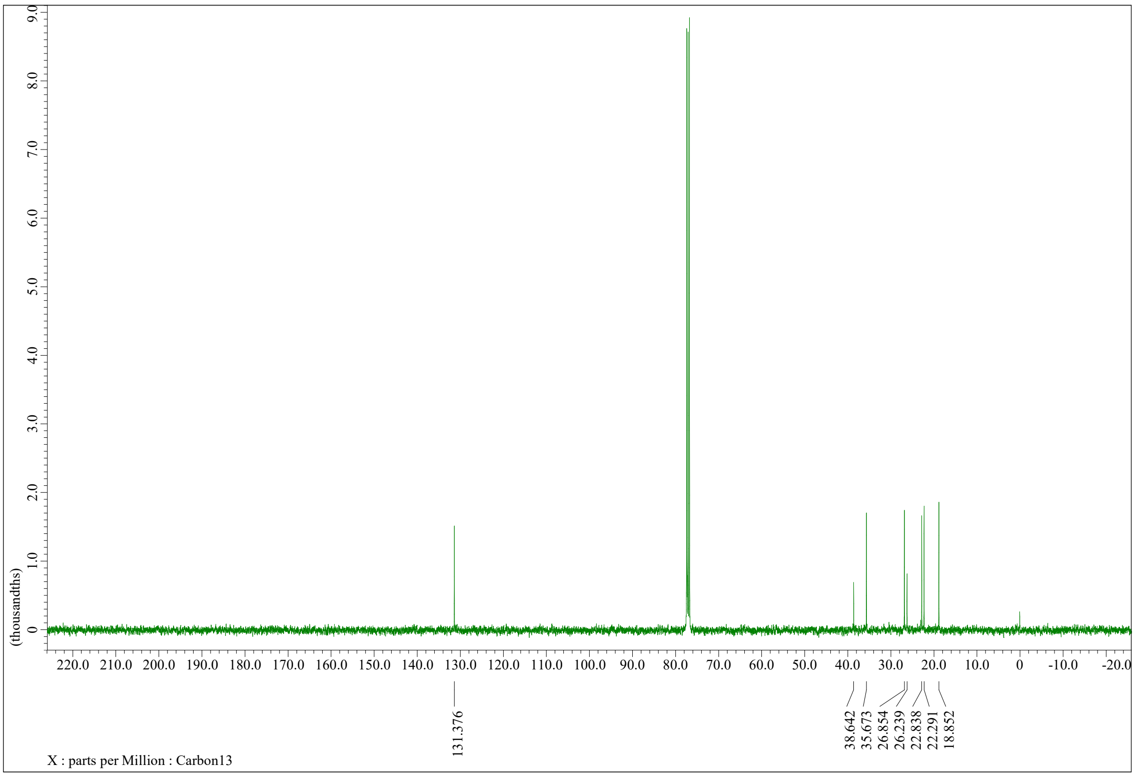

3i: ^{1}H NMR (400 MHz, CDCl$_3$)

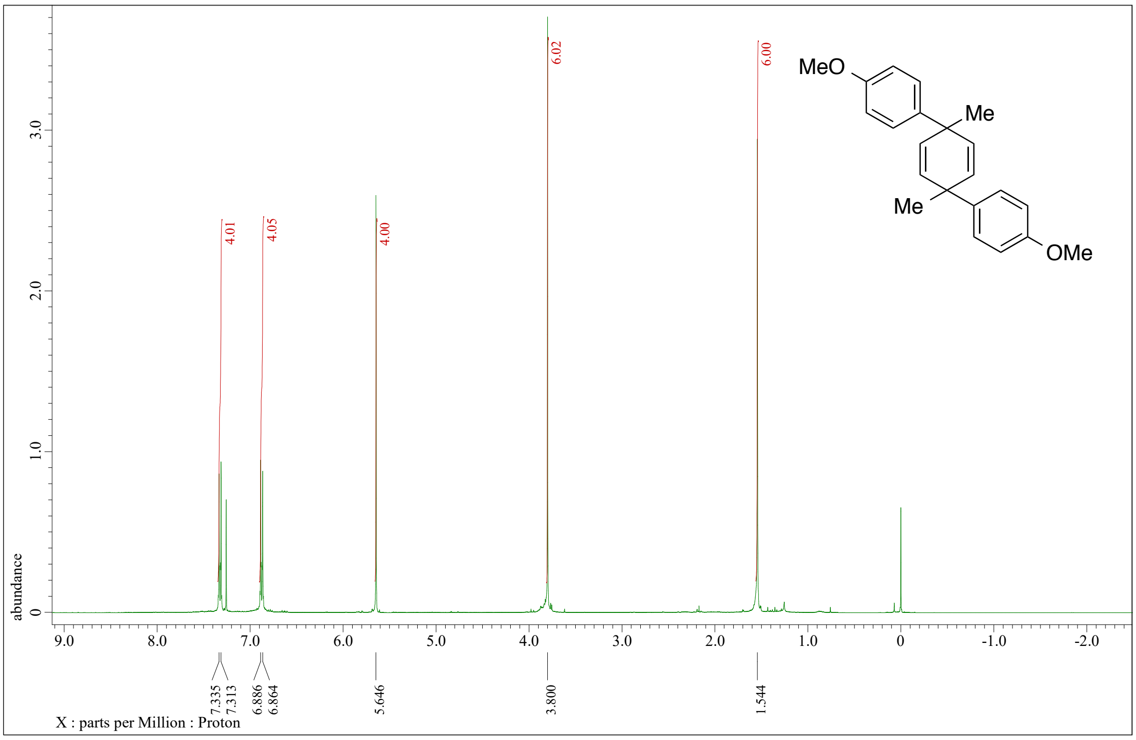

3i: ^{13}C{^{1}H} NMR (100 MHz, CDCl$_3$)

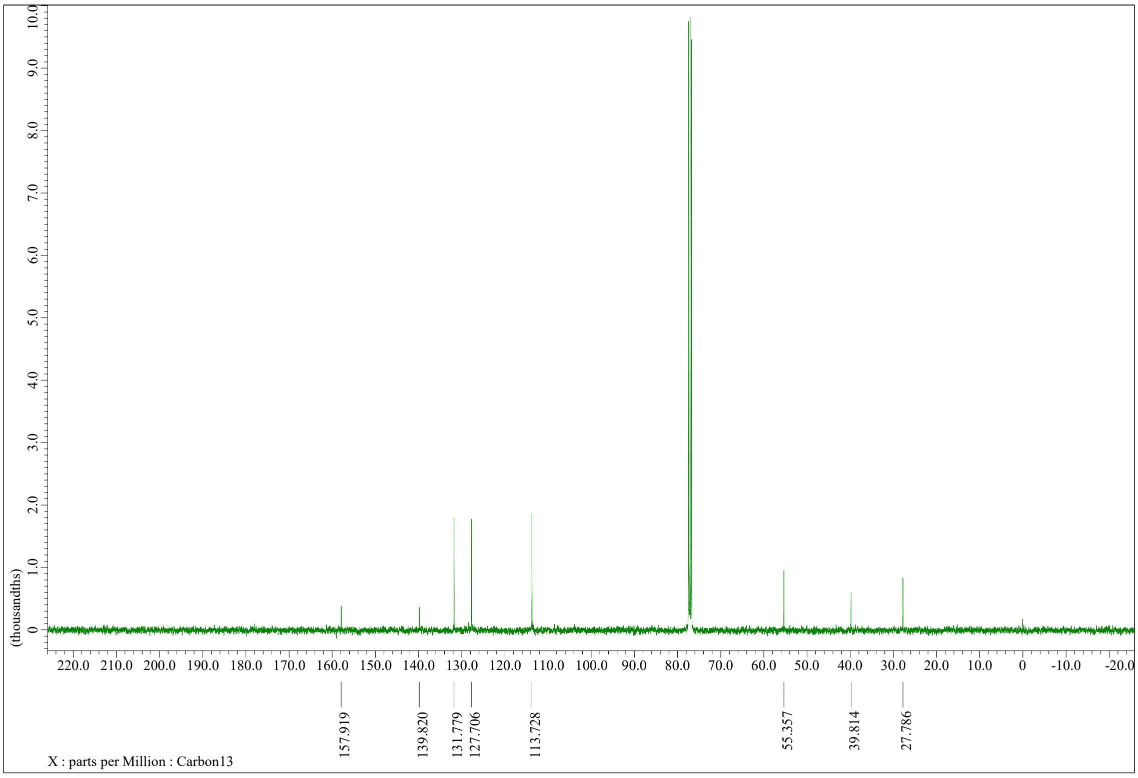

3j: ^{1}H NMR (400 MHz, $CDCl_3$)

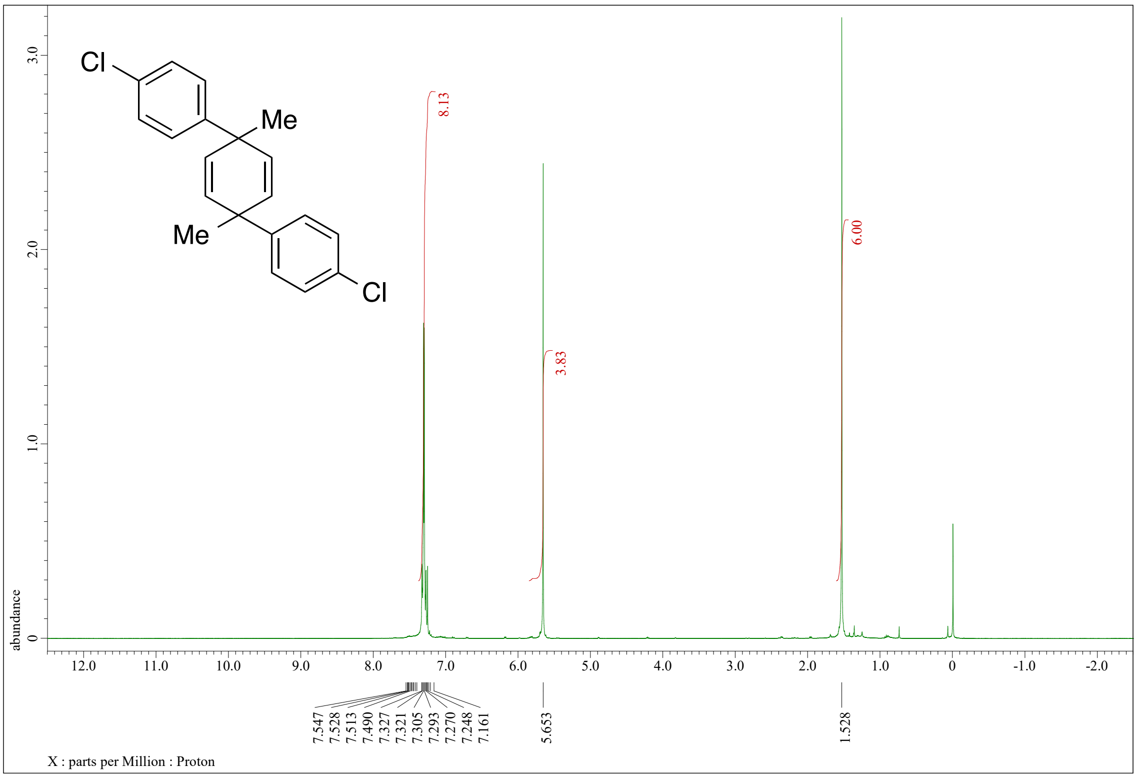

3j: ^{13}C{^{1}H} NMR (100 MHz, $CDCl_3$)

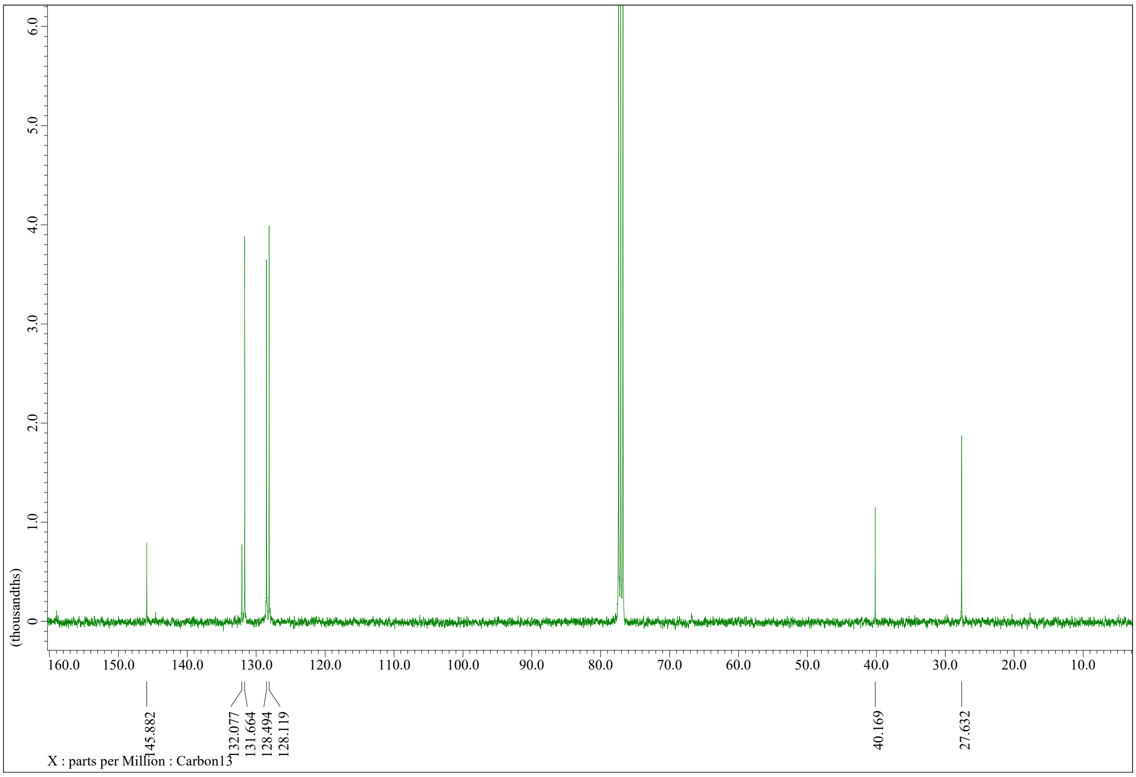

3k: ^{1}H NMR (400 MHz, $CDCl_3$)

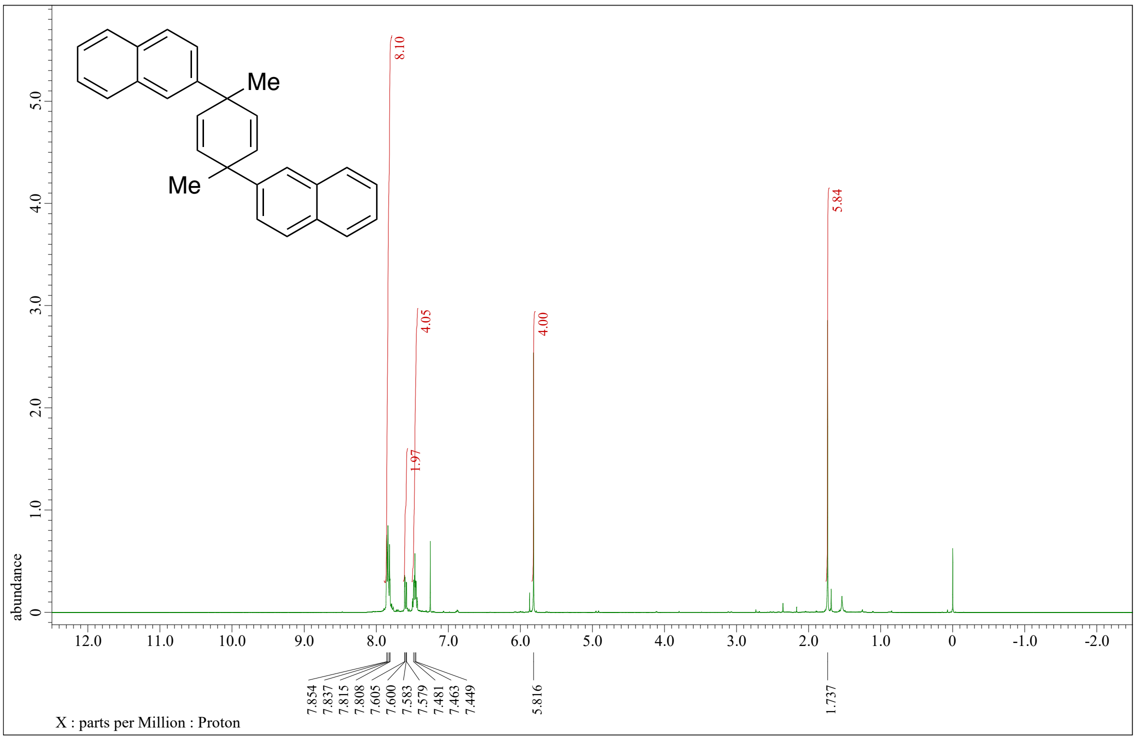

3k: ^{13}C{^{1}H} NMR (100 MHz, $CDCl_3$)

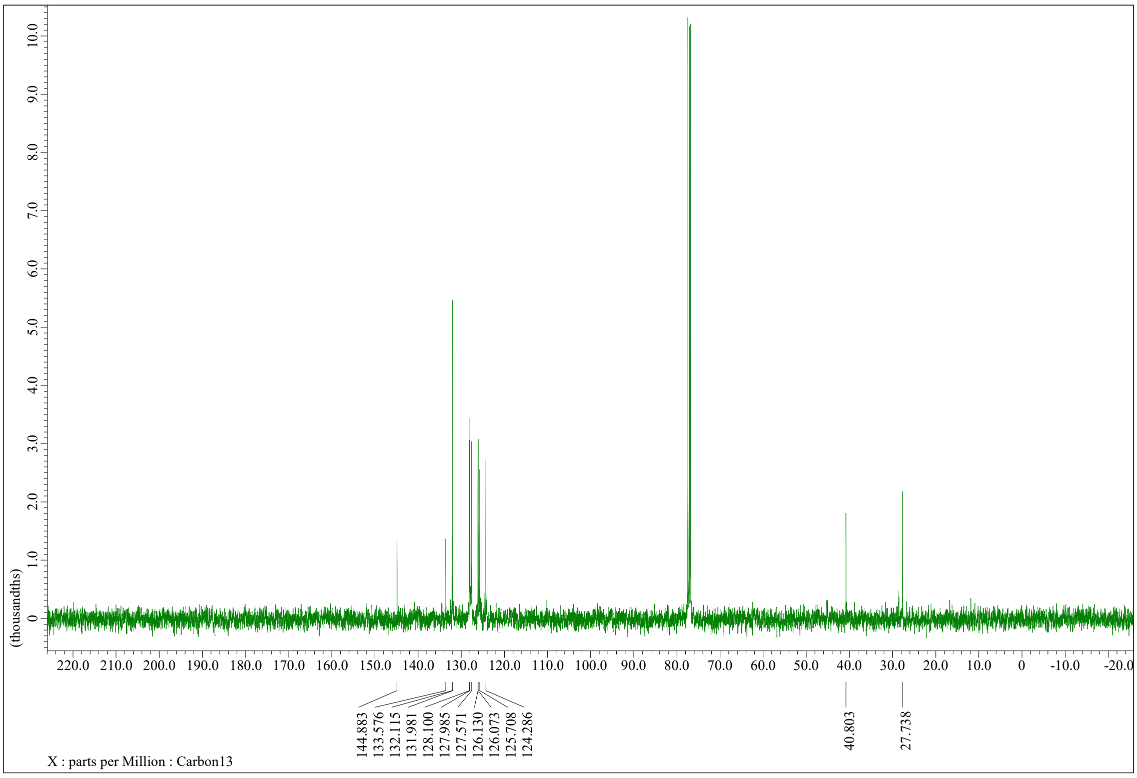

4a: ^{1}H NMR (400 MHz, $CDCl_3$)

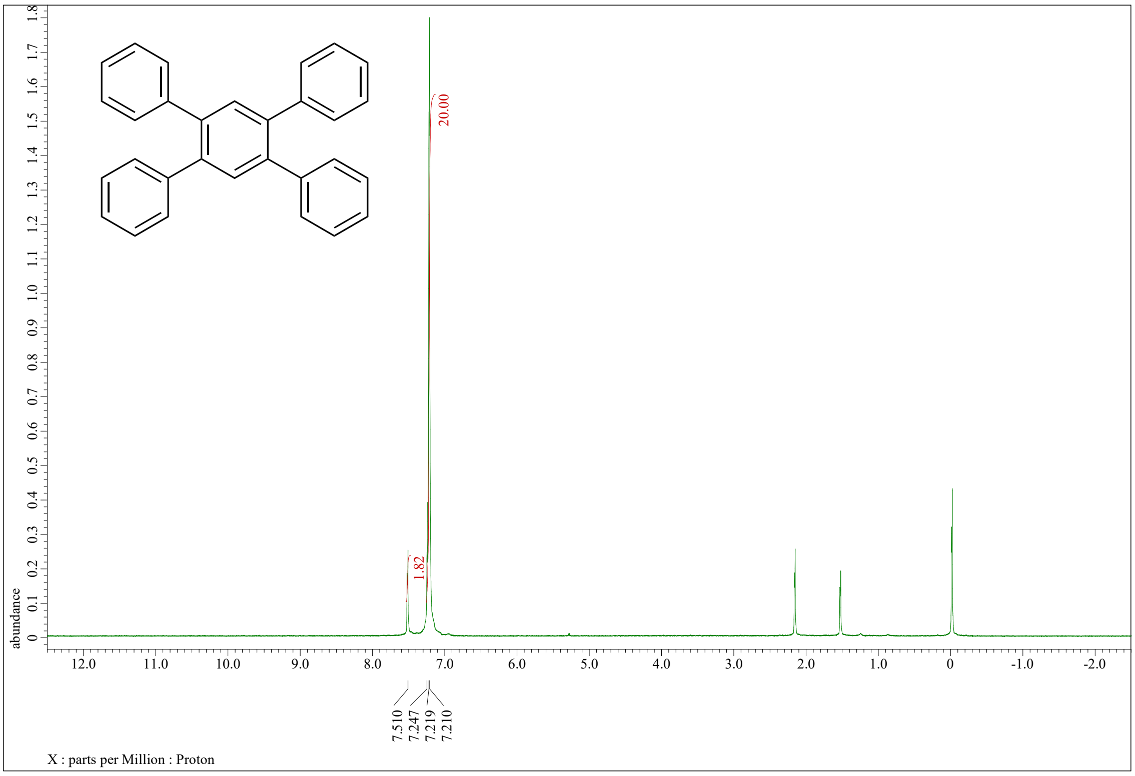

4a: $^{13}C\{^{1}H\}$ NMR (100 MHz, $CDCl_3$)

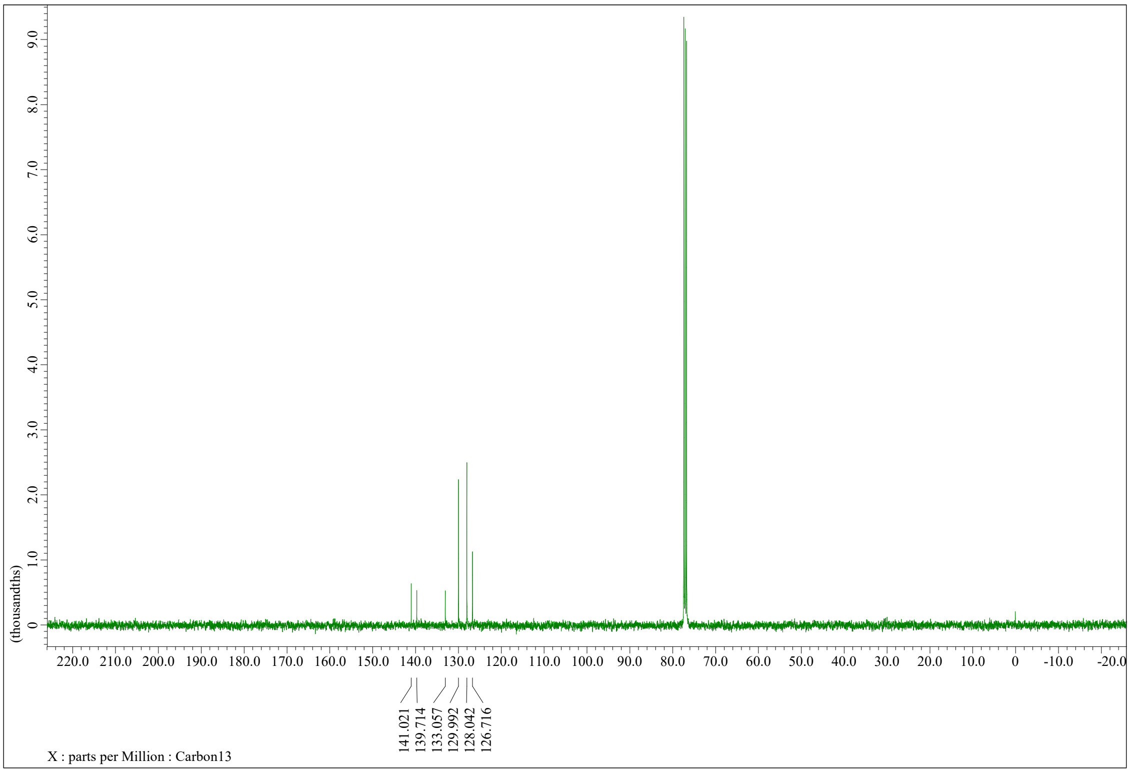

4b: ^{1}H NMR (400 MHz, $CDCl_3$)

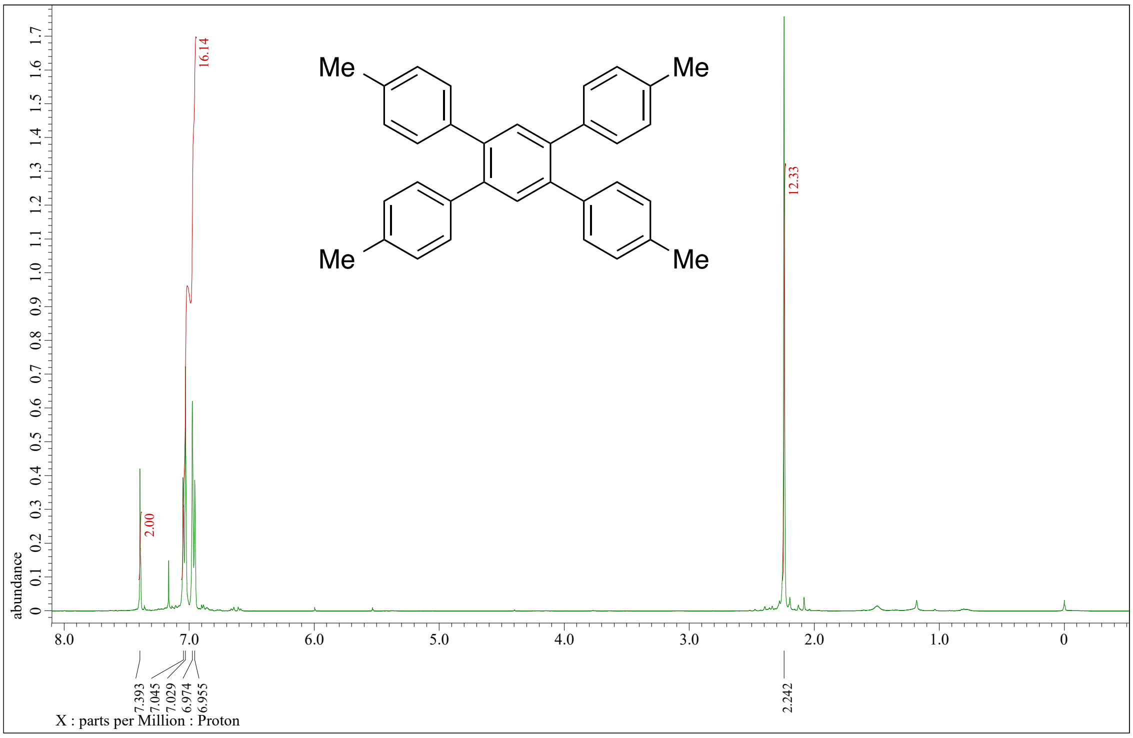

4b: ^{13}C{^{1}H} NMR (100 MHz, $CDCl_3$)

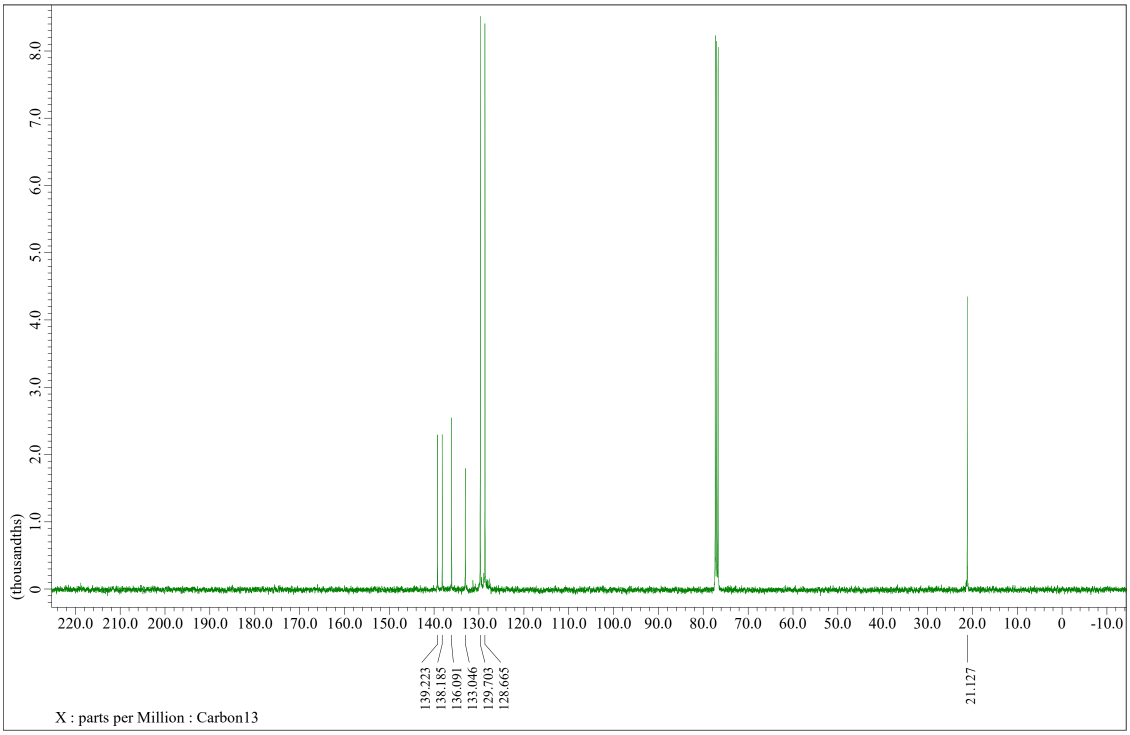

4c: ^{1}H NMR (400 MHz, $CDCl_3$)

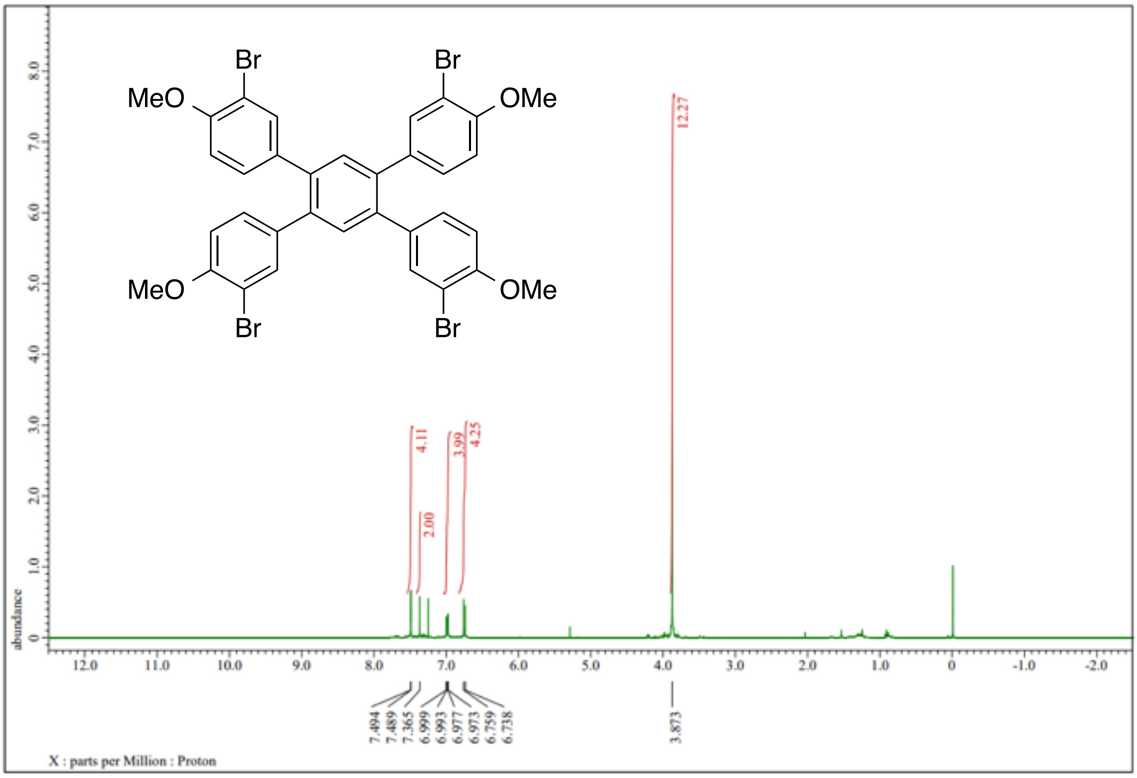

4c: $^{13}C\{^{1}H\}$ NMR (100 MHz, $CDCl_3$)

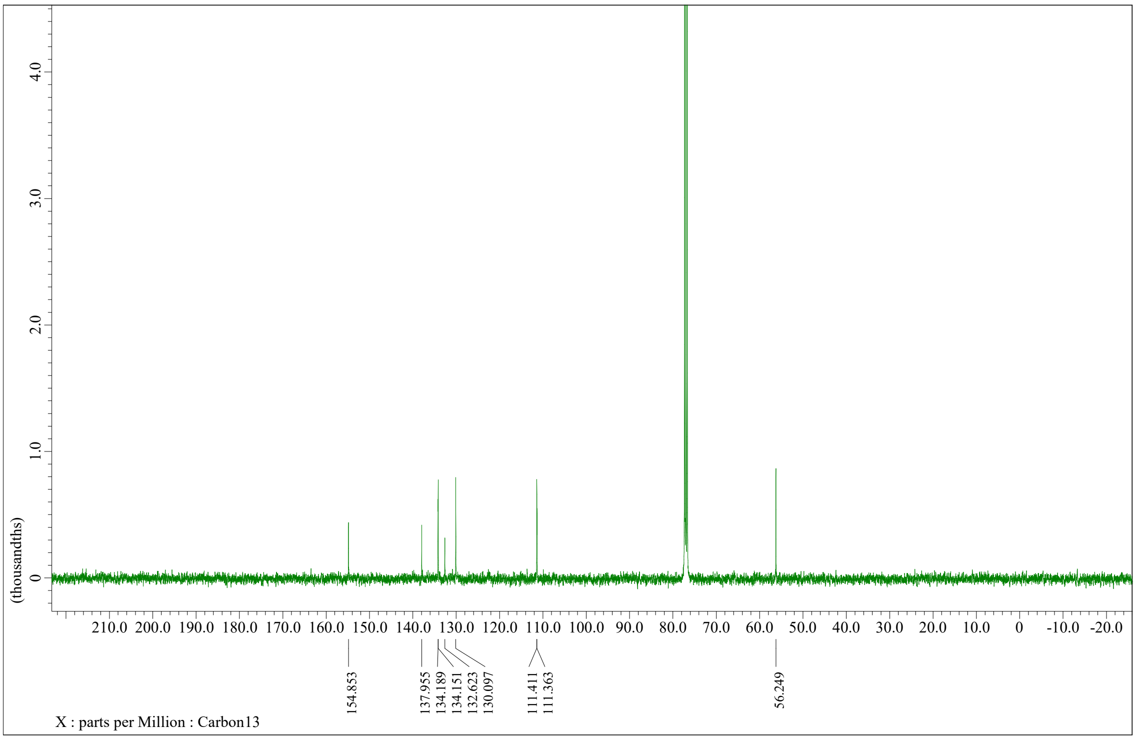

4d: ^{1}H NMR (400 MHz, $CDCl_3$)

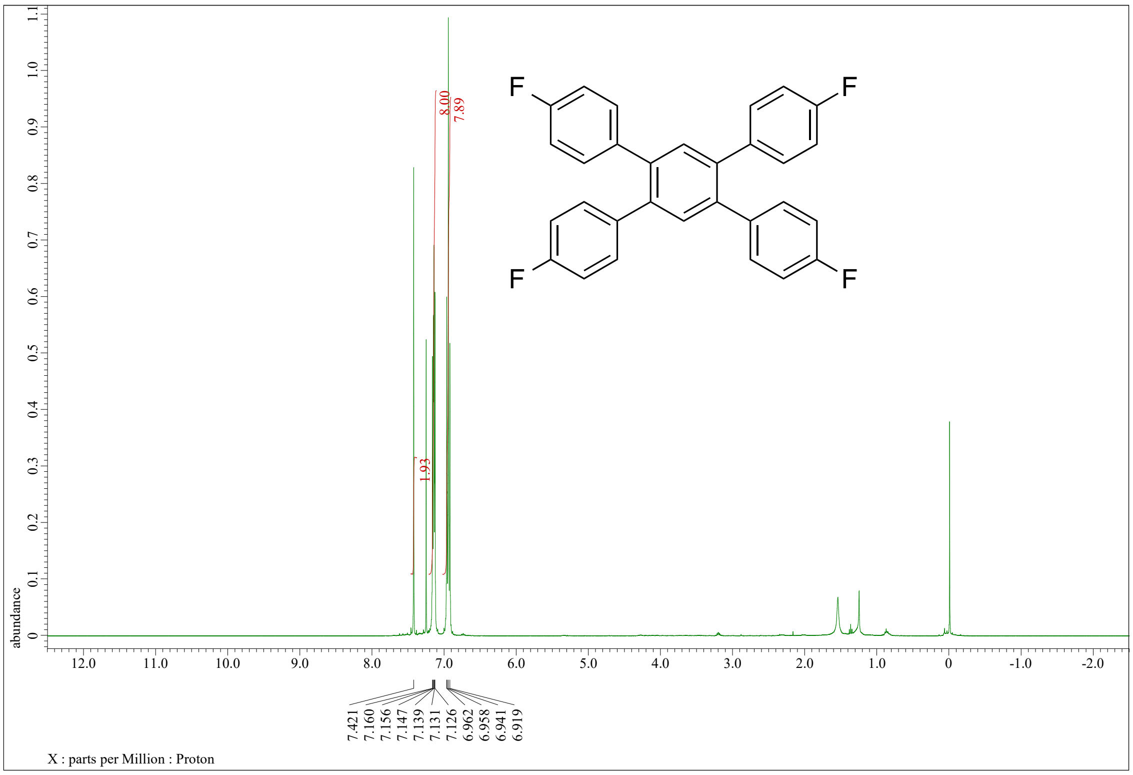

4d: $^{13}C\{^{1}H\}$ NMR (100 MHz, $CDCl_3$)

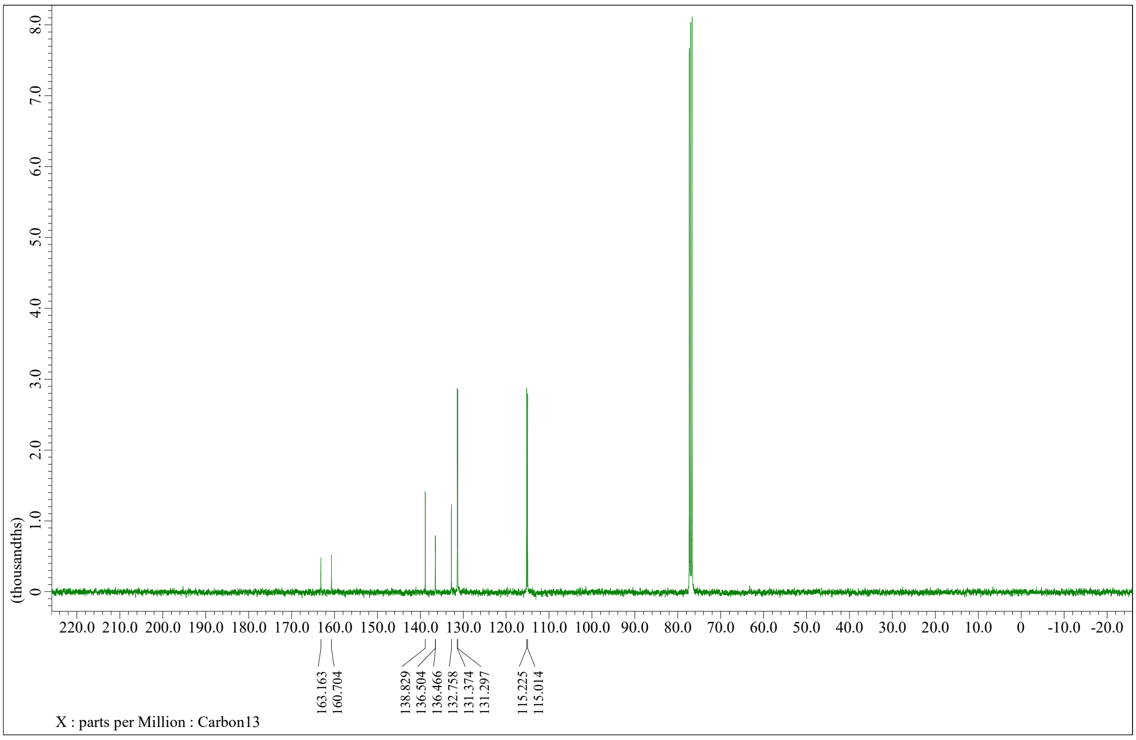

4d: ^{19}F NMR (375 MHz, $CDCl_3$)

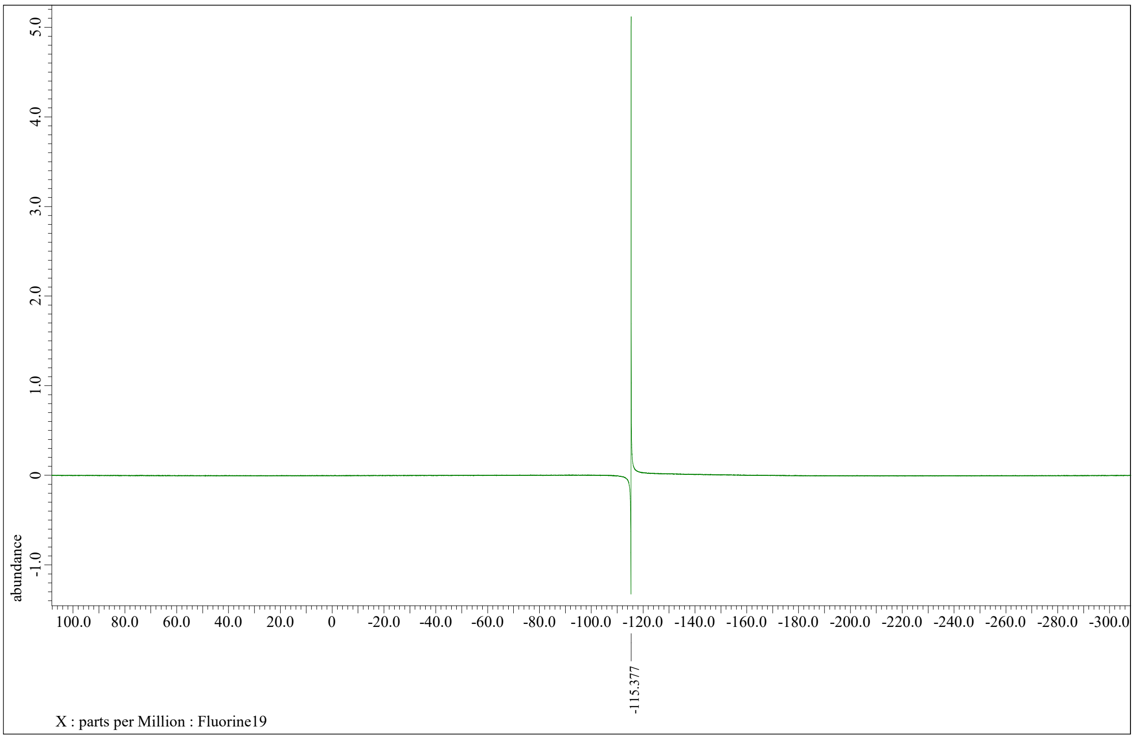

4e: ^{1}H NMR (400 MHz, $CDCl_3$)

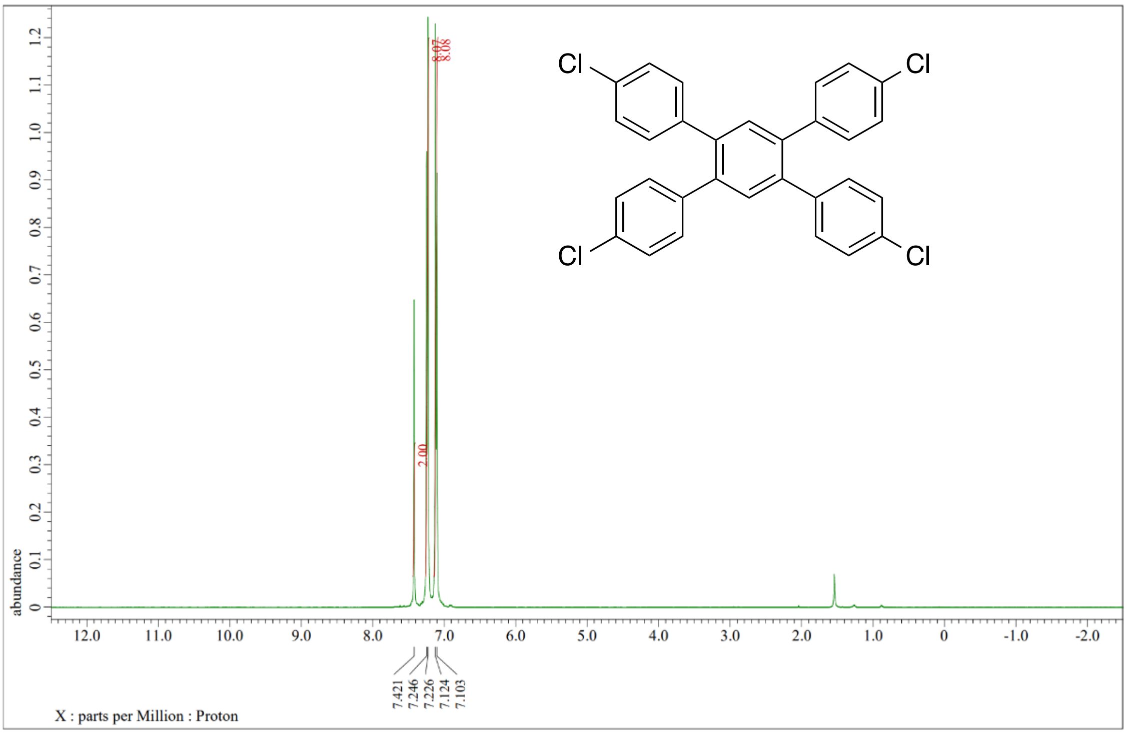

4e: $^{13}C\{^{1}H\}$ NMR (100 MHz, $CDCl_3$)

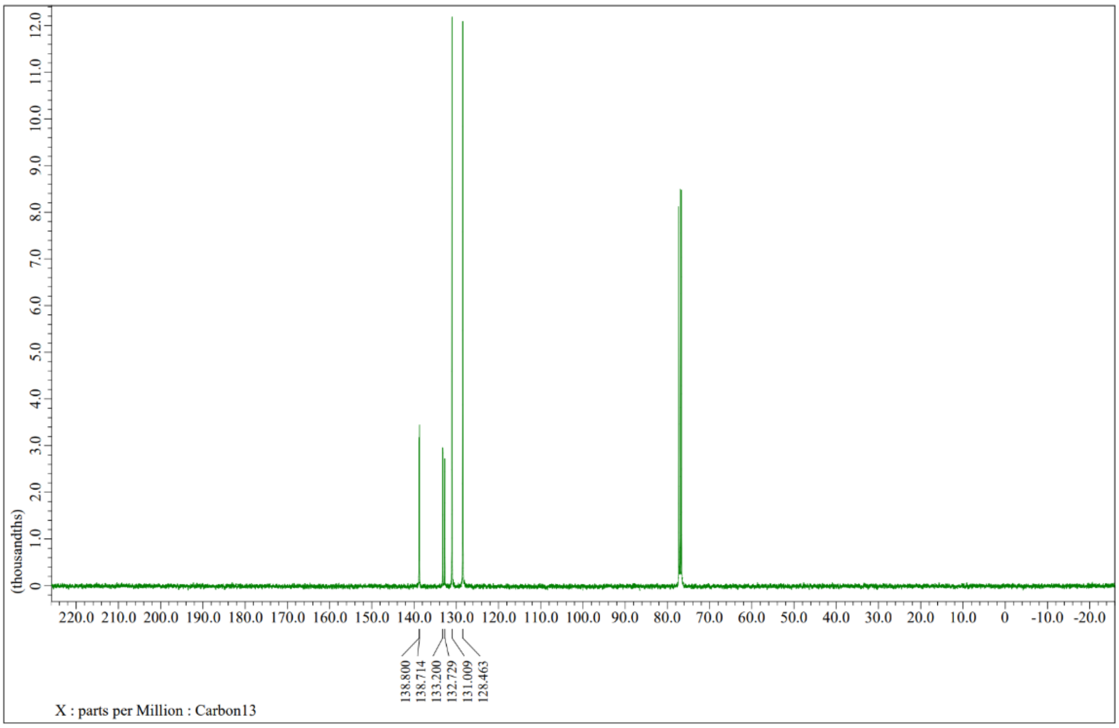

4f: ^{1}H NMR (400 MHz, $CDCl_3$)

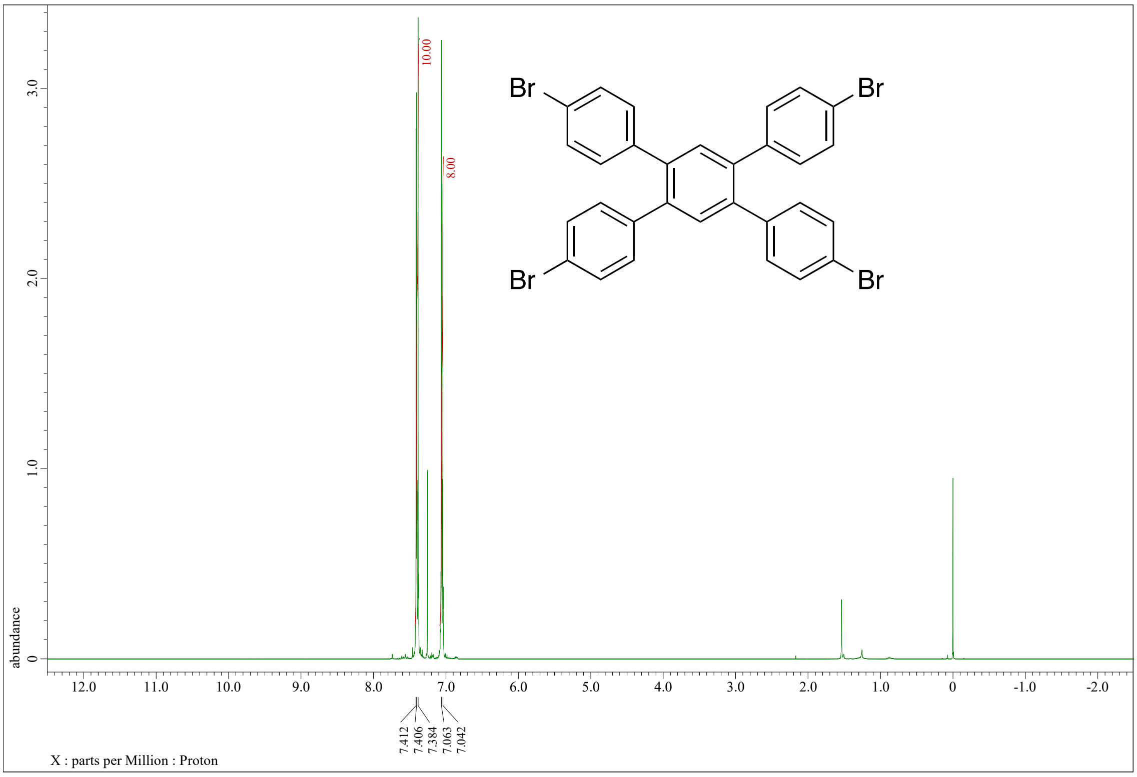

4f: ^{13}C{^{1}H} NMR (100 MHz, $CDCl_3$)

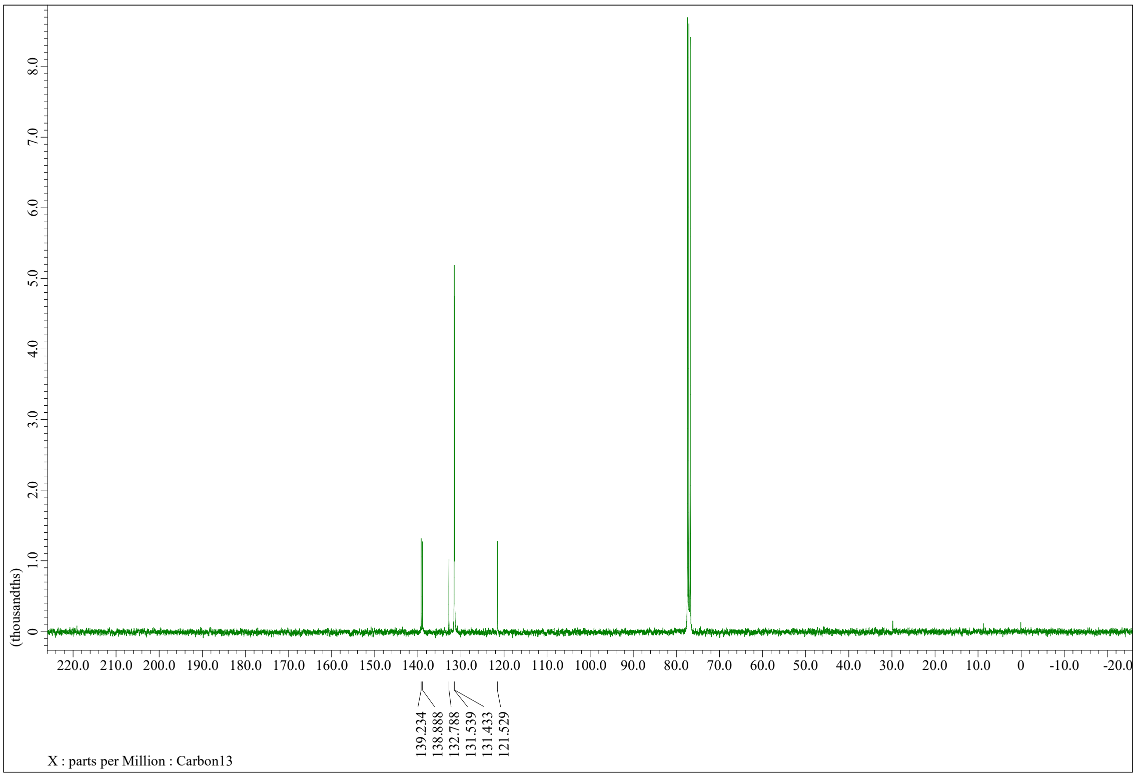

Reference:

[S1] Harvey, R. G.; Lindow, D. F.; Rabideau, P. W. Metal-Ammonia Reduction. XIII. Regiospecificity of Reduction and Reductive Methylation in the Terphenyl Series, *J. Am. Chem. Soc.* **1972**, *94*, 5412–5420.
